# Clinical Trial: A Multicentre Randomised Controlled Trial of Carvedilol Versus Variceal Band Ligation in Primary Prevention of Variceal Bleeding in Liver Cirrhosis (CALIBRE Trial)

**DOI:** 10.1111/apt.70080

**Published:** 2025-04-16

**Authors:** Dhiraj Tripathi, Kelly Handley, Lisa Holden, Zainab Abdali, Sue Jowett, Jonathan Mathers, Christopher Poyner, Paul Richardson, James Ferguson, Ian Rowe, CALIBRE trial collaborative group, Ashis Mukhopadhya, Jennifer Cathcart, Pauline Dundas, Oluyemi Solanke, Siby Joseph, Cyril Sieberhagen, Melanie Harrison, Claire Burston, Gavin Wright, Princess Gabiana, Aimee Williams, Bushena Miyesa, John Ramage, Hayley Cousins, Rachel Bryant, Sim L. Lim, Janisha Patel, Mel Penacerrada, Rachel Lorusso, Rory McCorry, Allison Lloyd, Heather Lawther, Safa Al‐Shamma, Anna Maria Wilce, Sulleman Moreea, Carol Firth, Robert Przemioslo, Charlotte Cranfield, Joanna Leithead, Gwilym Webb, Victoria Snowdon, Aarani Mahalingam, Abigail Ford, Thomas Pembroke, Sarah Harrhy, Fiona Greaves, Matthew Foxton, Carina Bautista, Esther Unitt, Susan Dale, Andrew Austin, Catherine Addleton, Sharan Shetty, Rizwan Mahmood, Clare Allcock, Karen Reid, Michael Miller, Anne Elliott, Leanne Cosgrove, Shona Murray, Francisco Porra Pérez, Melanie Kent, Marie Callaghan, Dina Mansour, Ann Wilson, Adrian Stanley, Alexis Duncan, Lauren Walker, Coral Hollywood, Paula Hiltout, Phil Berry, Jessica Cordle, Andrew King, Laurence Hopkins, Faye Moore, Mirriam Sangombe, Lynsey Corless, Tania Nurun, Julie Wilcox, Bethia Featherstone, Ameet Dhar, Maria Lanoria, Gary Hahn, Brian Hogan, Mark McPhail, Matthew M. Farrant, Nicola Griffiths, Ane Zamalloa, Victoria Appleby, Mark Aldersley, Rebecca Bishop, Louise Clark, Farooq Khan, Ka‐Kit Li, Olivia Watchorn, John Hutchinson, Patricia Kane, Julie Burton, Stephanie Lupton, Jacqui Bartholomew, Emma‐Jane Stoner, Steven Masson, Sarah Hogg, Simon Rushbrook, Vanessa Martin, Tom Lee, Jane Luke, Angela Green, Stephen Ryder, Preethi Shibu, Jeremy Cobbold, Loren Smith, Lou Holland, Eleni Rountenko, Matthew Cramp, Alex Parkinson, Richard Aspinall, Emily Jones, Jincy Daniels, Anu Andrews, Hyder Hussaini, Lisa Trembath, Fiona Hammonds, Ben Hudson, Rob James, Raj Mookerjee, Christine Eastgate, Gloria Ferrariz, Abigail Brownlie, Dai Samuel, Bethan Deacon, Imran Patanwala, Edward Britton, Callum Wood, Martina Lofthouse, Giovanna Bretland, Vikram Sharma, Louise Payaniandy, James Hand, Graham Branagan, Aqueel Jamil, Alpha Anthony, Louise Bell, Ahmed Abu‐Shanab, Samia Hussain, Manjinder Gohel, Charlie Millson, Kerry Elliott, Tania Neale, Laura Harrison, Barbara Hoeroldt, Ihteshamul Haq, Angela Liaros, Holly Caborn, Ulrich Thalheimer, Kate Winstance, Darren Craig, Julie Tregonning, Janisha Patel, Helen Stone, Lijamol Joseph, Jisha Jacob, Joanne Topping, Peter Murphy, Jill Effard, Sarah Hughes, Criscel Jan Pelaez, Rohit Sinha, Jenny Henderson, Jagdish Nagaraj, Chin Lye Ch’Ng, Caradog Thomas, Elaine Brinkworth, Moby Joseph, Andrew Claridge, James Neale, Camille Walling, Maggie Ryder, Jacinta Ugoji, Neil Rajoriya, Emma Burke, Nishant Gautam, Julie Fletcher, Richard Keld, Philip Paterson, Christopher Corbett, Marie Green, Siobhan Sutton, Naomi Gott, Peter Hayes, Peter Devine, Khaled Ahmed, Kate Brailsford, Peter Brocklehurst, Margaret Grant, Gemma Slinn, Matthew Soden, Rebecca Amos‐Hirst, Hannah Bensoussane, Catherine Moakes, Bhushan Chhajed, Paul Riley, Neil Winkles, Lazaros Andronis, Christel McMullan

**Affiliations:** ^1^ Liver Unit University Hospitals Birmingham NHS Foundation Trust, Birmingham Health Partners Birmingham UK; ^2^ Immunology and Immunotherapy, College of Medicine and Health University of Birmingham, Birmingham Health Partners Birmingham UK; ^3^ Birmingham Clinical Trials Unit University of Birmingham Birmingham UK; ^4^ Health Economics Unit, Department of Applied Health Sciences University of Birmingham Birmingham UK; ^5^ Applied Health Sciences School of Health Sciences College of Medicine and Health University of Birmingham Birmingham UK; ^6^ School of Social Policy and Society University of Birmingham Birmingham UK; ^7^ The Royal Liverpool Hospital NHS Foundation Trust Liverpool UK; ^8^ Leeds institute for Medical Research, University of Leeds Leeds UK; ^9^ Leeds Liver Unit Leeds Teaching Hospitals NHS Trust Leeds UK

**Keywords:** gastrointestinal bleeding, liver cirrhosis, oesophageal varices, portal hypertension

## Abstract

**Background:**

The superior efficacy of non‐selective beta‐blockers (NSBB) compared with variceal band ligation (VBL) in the primary prevention of variceal bleeding is uncertain.

**Aim:**

To compare carvedilol versus VBL for primary prevention of variceal bleeding.

**Methods:**

CALIBRE was an investigator‐initiated, multicentre, randomised, controlled, open‐label trial. Participants were randomly assigned to 12.5 mg carvedilol once daily or VBL. Inclusion criteria were cirrhosis and medium to large oesophageal varices that had not bled. The primary outcome is any variceal bleeding within 1 year of randomisation. Secondary outcomes include survival, other complications of cirrhosis, quality of life, cost‐effectiveness and adverse events. Recruitment closed early, mainly due to the impact of the pandemic.

**Results:**

265 participants (10% of the intended sample size) from 52 sites were randomised to carvedilol (*n* = 133) or VBL (*n* = 132) between 22 January 2019 and 31 August 2022. 5/133 participants (3.8%) in the carvedilol arm vs. 10/132 participants (7.6%) in the VBL arm experienced variceal bleeding (risk ratio 0.50 (95% confidence interval [CI]; 0.17–1.41); risk difference − 0.038 (95% CI; −0.094—0.017)). Serious adverse events occurred in one participant in each treatment arm, with no treatment‐related deaths. Of the secondary outcomes, there were no statistically significant differences. Carvedilol was cheaper and resulted in slightly more quality‐adjusted life years than VBL.

**Conclusions:**

The early terminated and, thus, underpowered CALIBRE trial showed no difference between **c**arvedilol and VBL in the primary prevention of variceal bleeding in patients with cirrhosis and medium‐to large‐sized oesophageal varices. No untoward safety concerns were noted.

**Trial Registration:**

ISRCTN73887615

## Graphical abstract

There is no evidence of a difference between carvedilol 12.5 mg daily and VBL in the primary prevention of variceal bleeding in patients with cirrhosis and medium‐to large‐sized oesophageal varices. Carvedilol has no untoward safety concerns, is well tolerated, cost‐saving and potentially cost‐effective and is the preferred first‐line primary prevention option.

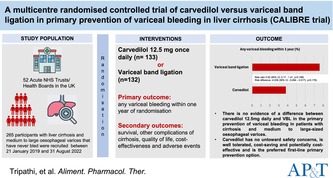

## 1 Introduction

Liver‐related deaths have increased by over 400% since 1970, and the number of hospital admissions for liver disease has escalated by half over the last decade. In patients with cirrhosis, varices develop at a rate of 5% per year, with a 10‐year cumulative incidence of 44% [1]. Variceal bleeding is a major complication of cirrhosis, with 3000 annual hospital admissions in England, an inpatient mortality of 15% and 1‐year mortality of up to 40% [2]. Hospital admission for variceal bleeding has a considerable impact on healthcare resources. Therefore, reducing the risk of the first variceal bleed (primary prevention) is an important clinical and economic goal.

Non‐selective beta‐blockers (NSBB) and variceal band ligation (VBL) are options for primary prevention. The NSBB carvedilol has a greater effect on portal pressure than propranolol or nadolol, as it is also a vasodilator due to alpha‐1 receptor antagonism, which reduces intrahepatic resistance [3]. Moreover, carvedilol can be effective even in propranolol non‐responders [4]. Carvedilol is also well tolerated. However, caution is advised in patients with decompensated liver disease due to increased bioavailability and increased risk of systemic hypotension [5]. Therefore, carvedilol was selected for this trial.

There has been an evolving evidence base comparing NSBB versus VBL in primary prevention. The first trial from the UK of 152 patients showed significantly reduced bleeding in the carvedilol arm (10% vs. 23%, relative hazard 0.41; 95% confidence interval [CI] 0.19–0.96), with no apparent effect on survival [6]. The second trial from Pakistan of 168 patients showed no differences in bleeding or mortality [7]. A Cochrane review and meta‐analysis of 19 trials (1504 patients) showed VBL had a greater effect in primary prevention compared with NSBB (risk ratio [RR], 0.67; 95% CI, 0.46–0.98) with no effect on survival [8]. However, the overall quality of evidence was low to moderate. The beneficial effects of VBL were not evident when only high‐quality studies were analysed. In another meta‐analysis, NSBB has more adverse events than VBL [9]. The uncertainty in the evidence at the time was reflected in conflicting recommendations for using NSBB or VBL as first‐line therapy in primary prevention in two UK guidelines [1, 10].

Data on cost‐effectiveness in primary prevention are available from just one publication [11]. This suggested that beta‐blockers reduced overall costs compared with VBL. There are no cost‐effectiveness studies alongside a RCT comparing carvedilol with VBL. There is also a lack of data on health‐related quality of life.

The primary aim of this study is to compare carvedilol versus VBL in preventing any variceal bleeding within 1 year of randomisation in patients with cirrhosis and medium to large oesophageal varices that have never bled. Other objectives include the effect of carvedilol and VBL on survival, development of other complications of cirrhosis and adverse events. The study also explores cost‐effectiveness, patient preference and the use of alternative or cross‐over therapies.

## 2 Methods

### 2.1 Study Design

CALIBRE is an investigator‐initiated, multicentre, pragmatic, randomised controlled, open‐label trial with an internal pilot. Sixty sites in UK Acute NHS Trusts/Health Boards participated, with 52 sites recruiting patients. An internal pilot commenced in January 2019, and after meeting progression criteria, recruitment continued until March 2020. A qualitative study exploring patient perception of the interventions was conducted during the pilot and published elsewhere [12]. Due to the impact of the COVID‐19 pandemic, recruitment was suspended in April 2020 after 207 participants were randomised. Following protocol revisions to enable key trial activities to be done remotely, the trial resumed between January 2022 and August 2022, recruiting 58 participants. However, poor recruitment reflected the impact of the pandemic on endoscopy services, an interim recommendation from the British Society of Gastroenterology to prescribe empirical NSBB without endoscopy and evidence supporting NSBB to prevent decompensation in patients with clinically significant portal hypertension (CSPH) [13, 14]. Therefore, following a recommendation by the TSC, the trial was closed to recruitment in August 2022, but follow‐up of all recruited participants continued [15, 16]. The trial was approved by a National Health Service (NHS) Research Ethics Committee (REC, reference number 18/NE/0296) and prospectively registered at ISCTRN (ISRCTN73887615) and EudraCT (2018‐002488‐24).

### 2.2 Participants

Participants were identified after they were referred for surveillance endoscopy, where consent for the trial could be obtained before the procedure. Full details of the consent process are available in the protocol [15, 16]. All participants gave written informed consent, either in person or by telephone, the latter in the presence of a witness. The inclusion criteria were cirrhosis and medium varices (Grade II varices that do not flatten on air insufflation and do not occlude the lumen) or large varices (Grade III varices which are larger than Grade II varices and occupy the whole lumen) that have never bled, as defined in the BSG guidelines [1]. Patients with portal vein thrombosis of any grade could be included. Patients on propranolol, carvedilol, or nadolol for primary prevention or those who have had band ligation were excluded. At baseline, a full history and examination were performed along with standard of care blood workup, office blood pressure and pulse. AUDIT‐C score was calculated where appropriate. Liver stiffness measurements (LSM) were done using Vibration Controlled Transient Elastography (VCTE) where available and appropriate. Only LSM values with interquartile range (IQR) ≤ 30% and successful readings ≥ 60% were included. A Quality‐of‐Life questionnaire (EQ‐5D‐5L) was administered to all participants. Detailed inclusion and exclusion criteria are illustrated in the protocol [15, 16].

All trial activities were recorded on paper Case Report Forms (CRFs) and transcribed to an electronic database.

### 2.3 Randomisation

Randomisation was provided by a secure 24‐h online randomisation system at Birmingham Clinical Trials Unit (BCTU), supplemented by a telephone randomisation service. After participant eligibility was confirmed and informed consent received, participants were randomised in a 1:1 ratio to either treatment with 12.5 mg carvedilol once daily or VBL. Both treatments were started on the same day as randomisation or as soon as possible thereafter. A minimisation algorithm was used to ensure balance in the treatment allocation over the following variables: presence or absence of hepatic decompensation (ascites or encephalopathy), size of the largest varix (Grade II or Grade III), age of the patient at randomisation (18–50, 51–70, > 70) and presence or absence of alcohol‐related liver disease.

### 2.4 Procedures

Participants in the carvedilol arm were prescribed carvedilol 12.5 mg once daily, administered orally. They were seen in a 4‐week follow‐up clinic to assess for short‐term adverse events (see below) and tolerance [1]. The process for treatment modifications in the event of intolerance is detailed in the protocol [15]. These participants were not offered routine endoscopic surveillance per standard of care [1]. Participants in the VBL arm had the procedure performed as per the BSG guidelines [1]. At the clinician's discretion, participants who were intolerant of either carvedilol or VBL could be crossed over to the other treatment at any point.

All patients were followed up in the clinic at 6 and 12 months following randomisation as per standard of care. Trial‐specific tasks at each visit are detailed in the protocol [15]. Participants were asked about adherence to their trial medication at each follow‐up visit, and adherence to VBL was recorded in the CRFs.

### 2.5 Outcomes

The primary outcome is any variceal bleeding within 1 year of randomisation. The first variceal bleed is defined as hematemesis and/or melena with either (1) endoscopic evidence of variceal bleeding or stigmata of recent haemorrhage and at least a 2 g/L reduction in haemoglobin within 24 h of admission or (2) massive upper gastrointestinal bleeding leading to death. The definition includes bleeding from banding ulceration [1]. Secondary outcomes include survival, other complications of cirrhosis, quality of life, cost‐effectiveness and adverse events. Further details are in the protocol [15].

The collection and reporting of Adverse Events (AEs) were in accordance with the Medicines for Human Use (Clinical Trials) Regulations 2004 and subsequent amendments. The protocol provides further details of safety reporting [6, 15, 16].

### 2.6 Statistical Analysis

The sample size calculation was based on published data from both a Cochrane review and meta‐analysis of variceal banding versus beta‐blockers [8] and the data from the first UK RCT of carvedilol published in this disease area [6]. The Cochrane meta‐analysis reported an overall 1‐year variceal bleeding rate of 12% in the variceal banding ligation group. The 1‐year bleeding rate was chosen for the primary outcome as Kaplan–Meier curves suggest that most variceal bleeding occurs in the first year after treatment [6]. To detect a 33% proportional difference in variceal bleeding rates (i.e., from 12% to 8%, a 4% absolute difference) between groups using a 2‐sided test for comparison of proportions with a 1:1 allocation ratio, 90% power, and a type I error rate of 5% (i.e., *α* = 0.05) requires 2362 participants (1181 per group). It was thought that a 33% proportional difference between the two treatment arms would be the minimum required to change clinical practice. Assuming and adjusting for a 10% attrition/loss to follow‐up rate (based on the similar patient population [6], which is thus a conservative estimate due to our shorter duration of follow‐up) increases the required sample size to 2630 participants in total (1315 per group).

All primary analyses (primary and secondary outcomes including safety outcomes) were by intention‐to‐treat (ITT). Participants were analysed in the intervention group to which they were randomised, and all participants were included whether or not they received the allocated intervention. The analysis population for the safety form comprised only those patients randomised to carvedilol.

Planned subgroup analyses were limited to the same variables used in the minimisation algorithm and also limited to the primary outcome. Tests for statistical heterogeneity would be performed before any examination of effect estimates within subgroups. The results of any subgroup analyses should be treated with caution and will be used for hypothesis generation only.

Every attempt was made to collect complete follow‐up data on all study participants to minimise missing data. Participants with missing primary outcome data were included in the primary analysis in the first instance. Sensitivity analyses included (1) an analysis of the adherent population, i.e., those participants who were adherent to their randomised allocation, and (2) a complete‐case analysis, that is, those participants who provided complete primary outcome data. Any sensitivity analyses will not, irrespective of their differences, supplant the planned primary analyses.

The objective of the trial is to test the superiority of one intervention to another. The null hypothesis is that there is no difference in the 1‐year variceal bleeding rate between the intervention groups. The alternative hypothesis is that there is a difference between the groups. For binary (i.e., yes/no) outcomes, unadjusted risk ratios and risk differences with 95% CIs were estimated from a log‐binomial model. Where possible, models were adjusted for the minimisation of variables. Time‐to‐event outcomes were analysed using standard survival analysis methods. Kaplan–Meier survival curves were constructed for visual presentation of time‐to‐event comparisons. Cox proportional hazard models were fitted to obtain unadjusted treatment effects, expressed as hazard ratios with 95% CIs. Continuous outcomes were reported using means and standard deviations, and adjusted mean differences and 95% CIs were estimated using linear regression models. Initially, the analysis models included adjustments for the minimisation variables. However, due to the early stopping of the trial, an insufficient number of participants/events were available, leading to non‐convergence of the adjusted models. Therefore, unadjusted models have been reported for all outcomes. A full statistical analysis plan is available in Appendix 2.

Analyses were carried out using SAS version 9.4 or Stata 18. An independent Data Monitoring Committee and a Trial Steering Committee were convened, which met annually.

A within‐trial economic evaluation was conducted from a National Health Service (NHS) perspective to calculate the incremental cost per variceal bleed avoided and cost per QALY gained. Patient‐level data on healthcare resource use was collected at 6 and 12 months on the interventions, primary and secondary care contacts, and unit costs applied. Quality‐adjusted life years (QALYs) were calculated for each participant using EQ‐5D‐5L responses at baseline, 6 months, and 12 months. Due to the level of missing EQ‐5D‐5L data, a complete‐case analysis was undertaken. More detailed health economics methods and a separate Health Economics Analysis Plan (HEAP) are available in Appendix 3.

### 2.7 Role of the Funding Source

The funder of the study had no role in study design, data collection, data analysis, data interpretation or writing of the report.

### 2.8 Patient and Public Involvement (PPI) Statement

A Birmingham Liver and Gastrointestinal PPI Group member leads PPI in protocol design. He contributed to clinical aspects and consent pathways and was consulted for protocol amendments. JF assisted him in this role.

There was patient representation in the Trial Steering Committee to support trial oversight.

## 3 Results

**FIGURE 1:**
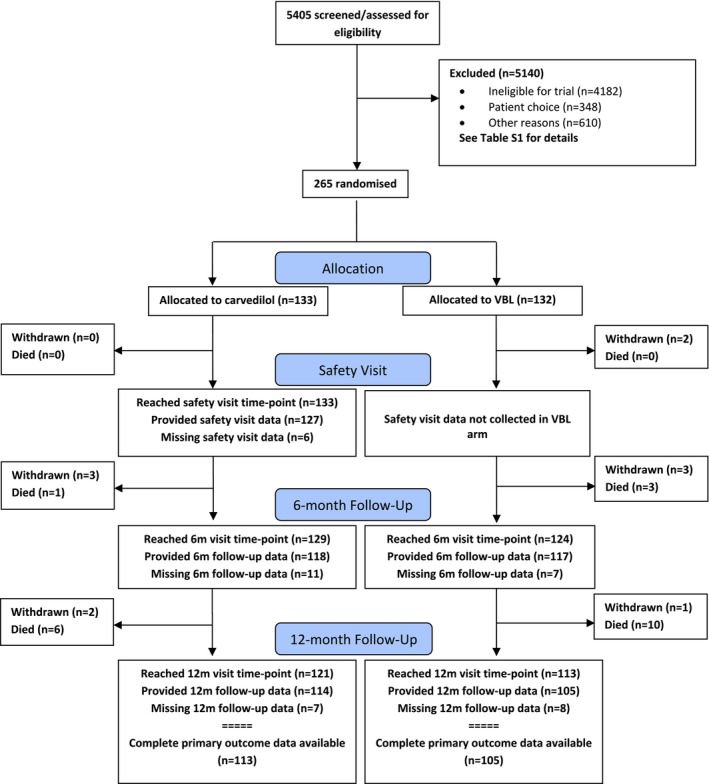
Consort flow diagram. Carvedilol, carvedilol 12.5 mg once daily orally. VBL, variceal band ligation as recommended in BSG guidelines.

Between January 21, 2019, and August 31, 2022, the trial recruited 265 participants from 52 sites in the United Kingdom. As detailed earlier, recruitment was suspended between March 2020 and December 2022 due to the COVID‐19 pandemic. From a total of 5403 participants screened, 133 were allocated to carvedilol and 132 to VBL (Figure 1). Following randomisation, 5 and 6 participants in the carvedilol and VBL arms withdrew consent of their own choice during follow‐up, respectively. Form completion rates for the 12‐month CRF were 114/133 (86%) for those allocated to carvedilol and 105/132 (80%) for VBL. The participants' baseline and clinical characteristics were similar in the two arms (Tables 1 and 2, Appendix 1, Section 5). Overall, 68.3% of participants were male, with a mean age of 59.6 ± 12.2 years, a median Child‐Pugh Score of 5 (IQR 5.6) and a median MELD score of 8 (IQR 7.10). 53.6% had alcohol‐related liver disease. Most participants had compensated liver disease (75.8%) and grade II oesophageal varices (93.2%), with 7.2% having gastric varices. Of the participants with decompensation at baseline, 75% in the carvedilol arm and 72% in the VBL arm had ascites, with 22% in the carvedilol arm and 6% in the VBL arm having hepatic encephalopathy (Table 1). For participants with alcohol‐related liver disease at randomisation, there was a significant increase in those abstaining for 12 months or more during follow‐up from 39% to 62% at baseline and 12 months, respectively, and reduced alcohol consumption amongst those not abstaining (Tables S12–S14). The mean LSM was 29.5 ± 16.9 kPa (*n* = 28 participants with valid data).

**TABLE 1:** Baseline characteristics.

		Carvedilol (*n* = 133)	VBL (*n* = 132)	Overall (*n* = 265)
*Randomisation and minimisation variables*				
Sex, *n* (%)	Male	89 (66.9%)	92 (69.7%)	181 (68.3%)
Age, years	Mean (SD)	59.7 (12.6)	59.5 (12.0)	59.6 (12.2)
Age category, *n* (%)	18–50	31 (23.3%)	28 (21.2%)	59 (22.3%)
	51–70	75 (56.4%)	79 (59.9%)	154 (58.1%)
	71+	27 (20.3%)	25 (18.9%)	52 (19.6%)
Hepatic decompensation, *n* (%)		32 (24.1%)	32 (24.2%)	64 (24.2%)
Alcohol‐related liver disease, *n* (%)		62 (46.6%)	61 (46.2%)	123 (46.4%)
Size of largest varix, *n* (%)	Grade II	123 (92.5%)	124 (93.9%)	247 (93.2%)
	Grade III	10 (7.5%)	9 (6.1%)	18 (6.8%)
Time from randomisation to treatment (days) Median [IQR]		0^a^	27.5 [9, 52]^b^	—
*Aetiology of cirrhosis*				
		(*n* = 116)	(*n* = 119)	(*n* = 235)
Alcohol‐related liver disease, *n* (%)		57 (49.1%)	58 (48.7%)	115 (48.9%)
Non‐alcoholic fatty liver disease, *n* (%)		43 (37.1%)	43 (36.1%)	86 (36.6%)
Primary sclerosing cholangitis, *n* (%)		2 (1.7%)	5 (4.2%)	7 (3.0%)
Primary biliary cholangitis, *n* (%)		7 (6.0%)	6 (5.0%)	13 (5.5%)
Hepatitis B, *n* (%)		1 (0.9%)	1 (0.8%)	2 (0.9%)
Hepatitis C, *n* (%)		9 (7.8%)	6 (5.0%)	15 (6.4%)
Sustained virologic response	No	*1*	*1*	*2*
	Yes	*8*	*4*	*12*
	Unknown	*0*	*1*	*1*
Autoimmune hepatitis, *n* (%)		8 (6.9%)	5 (4.2%)	13 (5.5%)
Other, *n* (%)		10 (8.6%)	12 (10.1%)	22 (9.4%)
Derived scores				
MELD score	*N*	93	94	187
	Median [IQR]	8 [7, 10]	8 [7, 10]	8 [7, 10]
Child‐Pugh	*N*	89	91	180
Score	Median [IQR]	5 [5, 6]	5 [5, 6]	5 [5, 6]
Stage	Grade A	70 (78.7%)	73 (80.2%)	143 (79.4)
	Grade B	19 (21.3%)	18 (19.8%)	37 (20.6%)
*Vital signs, and anthropometric data*				
		(*n* = 133)	(*n* = 131)	(*n* = 264)
Systolic BP, mmHg	*N*	129	129	258
	Mean (SD)	133 (18)	134 (20)	134 (19)
Diastolic BP, mmHg	*N*	129	129	258
	Mean (SD)	76 (13)	76 (12)	76 (13)
Pulse, beats/min	*N*	129	129	258
	Mean (SD)	81 (14)	83 (15)	82 (14)
BMI, kg/m^2^	*N*	127	122	249
	Mean (SD)	29.2 (5.5)	29.7 (7.0)	29.4 (6.3)
*Fibroscan*				
		(*n* = 133)	(*n* = 131)	(*n* = 264)
Fibroscan data available, *n* (%)		14 (10.5%)	14 (10.7%)	28 (10.6%)
Probe type, *n* (%)	Medium	7 (50.0%)	9 (64.3%)	16 (57.1%)
	Extra large	4 (28.6%)	3 (21.4%)	7 (25.0%)
	Unknown	3 (21.4%)	2 (14.3%)	5 (17.9%)
Liver stiffness measurement (kPa)	*N*	14	14	28
	Mean (SD)	25.5 (14.0)	33.6 (18.9)	29.5 (16.9)

^a^ Time from randomisation to carvedilol prescription (*n* = 119).

^b^ Time from randomisation to first endoscopy (*n* = 108).

**TABLE 2:** Baseline blood test results split by treatment group and overall.

		Carvedilol (*n* = 133)	VBL (*n* = 132)	Overall (*n* = 265)
*Blood test results*				
Sodium (mmol/L)	*N*	132	131	263
	Median [IQR]	139 [137, 141]	139 [136, 141]	139 [137, 141]
INR	*N*	101	97	198
	Median [IQR]	1.2 [1.1, 1.3]	1.2 [1.1, 1.3]	1.2 [1.1, 1.3]
Albumin (g/L)	*N*	132	129	261
	Median [IQR]	38 [33, 42]	36 [32, 40]	37 [32, 41]
Creatinine (μmol/L)	*N*	131	131	262
	Median [IQR]	67 [57, 79]	69 [57, 80]	67 [57, 80]
Bilirubin (μmol/L)	*N*	130	131	261
	Median [IQR]	21 [13, 30]	21 [11, 35]	21 [12, 33]
Alkaline phosphatase (U/L)^a^	*N*	116	118	234
	Median [IQR]	125 [94, 163]	132 [101, 183]	127 [96, 175]
eGFR (mL/min)^a^	*N*	106	112	218
	Median [IQR]	90 [60, 90]	90 [60, 90]	90 [60, 90]
Platelets (U/L)^a^	*N*	113	112	225
	Median [IQR]	112 [83, 142]	109 [82, 145]	112 [82, 143]
Ferritin (ug/L)^b^	*N*	14	13	27
	Median [IQR]	82 [35, 238]	107 [37, 192]	93 [35, 225]
AST (U/L)^b^	*N*	15	14	29
	Median [IQR]	50 [28, 74]	57 [47, 69]	56 [39, 69]
ALT (U/L)^b^	*N*	30	27	57
	Median [IQR]	34 [18, 55]	34 [17, 56]	34 [18, 55]
GGT (U/L)^b^	*N*	15	17	32
	Median [IQR]	216 [159, 350]	253 [123, 411]	222 [135, 381]

^a^ Added on version 2 of baseline form.

^b^ Added on version 4 of baseline form.

In the carvedilol arm, 5 out of 133 participants (3.8%) experienced variceal bleeding compared with 10 out of 132 (7.6%) participants in the VBL arm (in two participants, bleeding was related to VBL). Using an unadjusted log‐binomial model, this represents a risk ratio of 0.50 (95% CI; 0.17–1.41, *p* = 0.189) and a risk difference of −0.038 (95% CI; −0.094—0.017, *p* = 0.178) with no significant difference between the treatment arms. Sensitivity analysis of the primary outcome included an analysis of the adherent population (Appendix 1, Section 6) which was consistent with the overall result—1/85 (1.2%) in the carvedilol arm and 3/61 (4.9%) in the VBL arm experienced variceal bleeding (risk ratio 0.24 (95% CI: 0.03, 2.25), *p* = 0.211; risk difference − 0.037 (−0.096, 0.022), *p* = 0.213). An additional sensitivity analysis considered only those participants who provided complete primary outcome data—5/113 (4.4%) in the carvedilol arm and 10/105 (9.5%) in the VBL arm (risk ratio 0.46 (95% CI: 0.16, 1.31), *p* = 0.149, risk difference − 0.051 (−0.119, 0.017), *p* = 0.140). Due to the long pause in recruitment because of COVID‐19, it was decided to investigate the primary outcome in the first recruitment period (Jan 2019 and Mar 2020) and the second recruitment period (Jan 2022 and Aug 2022). However, the small number of events meant that no obvious pattern was seen.

**FIGURE 2:**
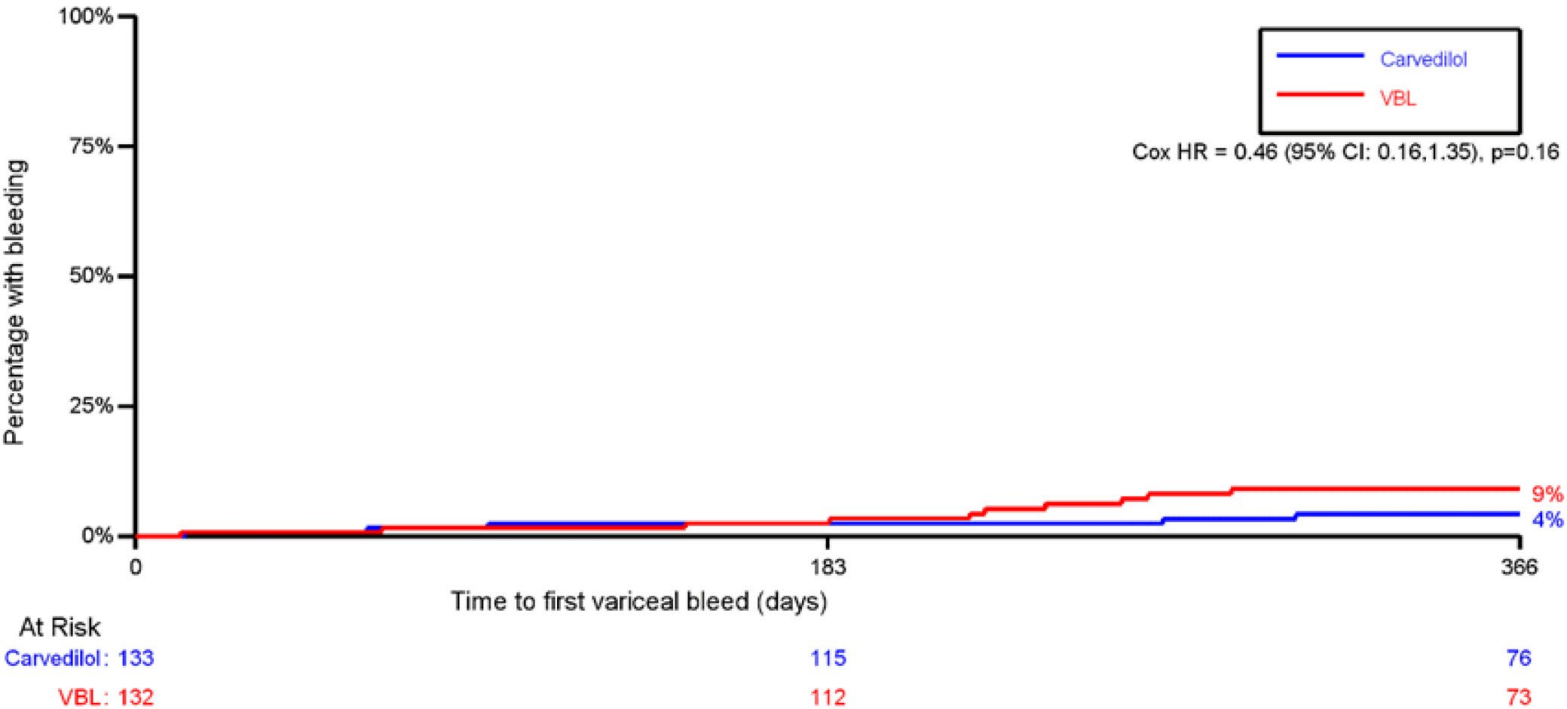
Kaplan Meier: time to first variceal bleed. HR, hazard ratio; VBL, variceal band ligation.

An analysis of secondary outcome measures includes time to first variceal bleed. There was no significant difference between carvedilol and VBL (3.8% vs. 7.6%, Cox Hazard Ratio 0.46 (95% CI; 0.16–1.35, *p* = 0.159)). See Table 3 and Figure 2. Likewise, there was no significant difference in 1‐year mortality (there were 8 liver‐related deaths, 1 cardiovascular mortality, and 12 cause unrecorded), transplant‐free survival, new‐onset ascites, spontaneous bacterial peritonitis, hepatorenal syndrome, hepatocellular carcinoma, or hepatic encephalopathy (see Table 3 and Appendix 1, Section 8). Exploratory analyses comparing overall hepatic decompensation (with two definitions) for compensated and all participants are provided in Appendix 1, Section 9.

**TABLE 3:** Primary and secondary clinical outcomes.

	Carvedilol (*n* = 133)	VBL (*n* = 132)		Estimate (95% CI)	*p*
*Primary outcome*					
Variceal bleeding within 1 year of randomisation^a^	5/133 (3.8%)	10/132 (7.6%)	Risk Ratio	0.50 (0.17, 1.41)	0.189
			Risk Difference	−0.038 (−0.094, 0.017)	0.178
*Secondary outcomes*					
Time to First Variceal Bleed	5/133 (3.8%)	10/132 (7.6%)	Cox HR	0.46 (0.16, 1.35)	
Mortality within 1 Year of Randomisation	8/121 (6.6%)	13/119 (10.9%)	Risk Ratio	0.61 (0.26, 1.41)	
			Risk Difference	−0.043 (−0.115, 0.028)	
	8/133 (6.0%)	13/132 (9.9%)	Cox HR	0.58 (0.24, 1.41)	
Transplant free survival	8/121 (6.6%)	13/119 (10.9%)	Risk Ratio	0.61 (0.26, 1.41)	
			Risk Difference	−0.043 (−0.115, 0.028)	
	8/133 (6.0%)	13/132 (9.9%)	Cox HR	0.58 (0.24, 1.40)	
New onset ascites	7/114 (6.1%)	11/108 (10.2%)	Risk Ratio	0.60 (0.24, 1.50)	
			Risk Difference	−0.040 (−0.113, 0.032)	
	7/133 (5.3%)	11/132 (8.3%)	Cox HR	0.61 (0.24, 1.57)	
Spontaneous bacterial peritonitis	2/114 (1.8%)	3/106 (2.8%)	Risk Ratio	0.62 (0.11, 3.64)	
			Risk Difference	−0.011 (−0.051, 0.029)	
	2/133 (1.5%)	3/132 (2.3%)	Cox HR	0.64 (0.11, 3.82)	
Hepatorenal syndrome	1/113 (0.9%)	0/105 (0%)	Risk Ratio	—	
			Risk Difference	—	
	1/133 (0.8%)	0/132 (0%)	Cox HR	—	
Hepatocellular carcinoma	2/114 (1.8%)	4/107 (3.7%)	Risk Ratio	0.47 (0.09, 2.51)	
			Risk Difference	−0.020 (−0.063, 0.023)	
	2/133 (1.5%)	4/132 (3.0%)	Cox HR	0.49 (0.09, 2.68)	
Hepatic encephalopathy	9/114 (7.9%)	8/106 (7.6%%)	Risk Ratio	1.05 (0.42, 2.61)	
			Risk Difference	0.004 (−0.067, 0.074)	
	9/133 (6.8%)	8/132 (6.1%)	Cox HR	1.08 (0.42, 2.79)	

*Note:* For the risk ratio, a point estimate below 1.0 favours the carvedilol group. For the risk difference, a point estimate below 0 favours the carvedilol group. The Cox model hazard ratio, 95% confidence interval, and *p*‐value are from the unadjusted model. A hazard ratio below 1.0 favours the carvedilol group. Participants are included in the risk ratio and risk difference models if they provide a complete 12‐month follow‐up. All participants are included in Cox model analyses as censoring can be incorporated to account for participants with less than complete follow‐up.

^a^ Sensitivity analysis of primary outcome only including patients with events or at least 305 days of data did not change the primary outcome 5/113 (4.4%) versus 10/105 (9.5%) for carvedilol and VBL respectively (risk ratio 0.46 (0.16, 1.31), *p* = 0.149; risk difference −0.051 (−0.119, 0.017), *p* = 0.140).

Table 4 presents details of the safety visit in the carvedilol arm. Compared with baseline, there was no evidence of a difference in systolic blood pressure (133 ± 18 mmHg vs. 129 ± 18 mmHg) and diastolic blood pressure (76 ± 13 mmHg vs. 74 ± 13 mmHg), but there was possible evidence of a reduction in the pulse (81 ± 14 mmHg vs. 72 ± 14). A total of 59 (46%) participants experienced adverse events. The dose of carvedilol was reduced due to adverse events in 25 (19.7%) participants, with the most common adverse events being dizziness and lethargy. Carvedilol was stopped due to adverse events in 14 (11%) participants, mainly due to lethargy, gastrointestinal upset, dizziness and shortness of breath. In 10 participants, alternative treatment was commenced, either VBL (*n* = 9) or propranolol (*n* = 1). In the VBL arm, dysphagia was noted in 12 (10.1%) of participants, and in the carvedilol arm, dyspnoea (11.5%) and gastrointestinal upset (9.8%) occurred but did not lead to treatment discontinuation. Two serious adverse effects deemed to be definitely related to treatment were noted in two participants. One participant with decompensated cirrhosis at baseline in the carvedilol arm experienced postural hypotension, and one participant with compensated cirrhosis at baseline in the VBL arm experienced pain and vomiting post‐banding.

**TABLE 4:** Carvedilol safety visit data (2–4 weeks).

	Carvedilol (*n* = 133)
	(*n* = 127)
GP visited in relation to liver cirrhosis/oesophageal varices, *n* (%)	10/122 (8.2%)
*Number of GP visits, n*	
1	7
2	1
3	1
4	
5	1
*Time from randomisation to carvedilol prescription, days* ^a^	
*N*	68
Median (IQR)	0 (0, 1)
*Systolic BP, mmHg*	
*N*	103
Mean (SD)	129 (18)
*Diastolic BP, mmHg*	
*N*	102
Mean (SD)	74 (13)
*Pulse, beats/min*	
*N*	103
Mean (SD)	72 (14)
Possible SAEs, *n* (%)	2/127 (1.6%)
*Adverse events, n (%)*	
Gastrointestinal upset	22/127 (17.3%)
Blurred vision	6/127 (4.7%)
Dizziness	25/127 (19.7%)
Lethargy	36/127 (28.3%)
Rash	6/127 (4.7%)
Headache	13/127 (10.2%)
Swelling in feet or hands	12/127 (9.4%)
URTI	2/127 (1.6%)
Shortness of breath	8/127 (6.3%)
Sexual dysfunction	1/127 (0.8%)
Other	29/127 (22.8%)
*Carvedilol dose split or reduced due to adverse events, n (%)*	25/127 (19.7%)
Gastrointestinal upset	3
Blurred vision	1
Dizziness	10
Lethargy	9
Rash	1
Headache	1
Swelling in feet or hands	3
URTI	
Shortness of breath	3
Sexual dysfunction	
Other	7
*Carvedilol stopped due to adverse events, n (%)*	14/127 (11.0%)
Gastrointestinal upset	5
Blurred vision	1
Dizziness	5
Lethargy	6
Rash	
Headache	1
*Swelling in feet or hands*	
URTI	1
Shortness of breath	4
Sexual dysfunction	
Other	8
Alternative treatment commenced, *n* (%)	10/126 (7.9%)
Propranolol	1
Variceal band ligation	9

*Note:* Participants randomised to carvedilol were followed up at 4 weeks post‐randomisation (protocol versions 1.0 and 2.0) or 2 weeks post‐randomisation (protocol versions 3.0 and 4.0) and assessed for any adverse events related to carvedilol.

^a^ Added on version 2 of safety form.

### 3.1 Economic Evaluation

Carvedilol was less costly, primarily due to low intervention costs, reduced need for surveillance endoscopies and slightly lower costs associated with managing liver complications. The results of the complete‐case cost‐utility analysis suggested that carvedilol was likely to be cost‐saving (mean difference, −£1053; 95% CI, −£2098 to £14.80) with a slight improvement in QALYs (0.010 [95% CI, −0.038 to 0.056]). Carvedilol had a 92% probability of being cost‐effective compared to VBL at the £20,000 per QALY threshold. The cost‐effectiveness analysis showed that carvedilol avoided slightly more bleeds and resulted in a cost‐saving of £1,608 (95% CI, −£3426 to £104). The findings remained consistent across different scenarios, with carvedilol continuing to be a cost‐saving option. Full details of the results are available in Appendix 1, Section 11.

## 4 Discussion

In participants with cirrhosis and medium to large oesophageal varices that have never bled, the CALIBRE trial, which was terminated early and thus underpowered, showed no difference between VBL and carvedilol in the primary prevention of variceal bleeding at 1 year. The results remained robust after analysis of the adherent participants, complete‐case analysis, and time to first variceal bleed analysis. There were no differences in any of the secondary outcome measures. Health economic analysis suggested that carvedilol may be cost‐saving with a marginal improvement in QALYs. There were no major safety concerns with both treatments. The results must be interpreted carefully, given that the trial did not recruit its intended sample size.

The evolving evidence base after the trial opened to recruitment was a factor in recruitment challenges, with a paradigm shift toward preventing hepatic decompensation in patients with CSPH as a clinical goal of NSBB therapy. The PREDESCI trial showed NSBB reduced the incidence of hepatic decompensation compared to placebo (16% vs. 27%; hazard ratio [HR] 0·51, 95% CI 0·26–0·97, *p* = 0·041) in compensated cirrhosis and CSPH based on hepatic venous pressure gradient (HVPG) estimation to NSBB [13]. These findings were supported by an individual patient data meta‐analysis, which showed that carvedilol reduced both decompensation (subdistribution HR [SHR] 0.506; 95% CI 0.289–0.887; *p* = 0.017) and mortality (SHR 0.417; 95% CI 0.194–0.896; *p* = 0.025) in patients with compensated cirrhosis with varices irrespective of their size or with HVPG ≥ 10 mmHg [14]. A recent individual patient data meta‐analysis showed no difference in variceal bleeding when comparing NSBB with endoscopic therapy [17]. However, in patients with compensated cirrhosis at baseline, there was a reduced mortality. Only two RCTs of carvedilol were included [6, 7]. A recent RCT found that combining banding and carvedilol treatment reduced first variceal bleeding and overall mortality compared to carvedilol alone in patients with Child‐Pugh stages A and B. [18] In contrast to CALIBRE, patients had more advanced disease, with a higher proportion of non‐alcoholic fatty liver disease and a significantly higher bleeding rate of 33% in the carvedilol arm. There were no data on quality of life.

This emerging evidence informed the recent update of NICE Cirrhosis guidelines, which has recommended carvedilol or propranolol as the first line in the primary prevention of variceal bleeding in patients with medium or large varices, with VBL recommended for patients unable to tolerate carvedilol or propranolol or if unable to take tablets regularly [19]. The primary prevention of decompensation with NSBB (carvedilol as the first choice, followed by propranolol) can be considered for patients with CSPH as defined by HVPG or the presence of varices [19]. International guidelines also support using NSBB to prevent the first decompensation in CSPH using invasive and/or non‐invasive criteria [20, 21, 22]. Recent data show that using non‐invasive criteria alone without endoscopy (LSM and platelet count) risks undertreating patients with varices [23]. In CALIBRE, although LSM were only available in a minority of patients, using the non‐invasive Baveno 7 criteria for CSPH (LSM ≥ 25 kPa) would have resulted in 60% of patients with medium to large varices not being treated with carvedilol. Where patients are not candidates for NSBB to prevent first decompensation (contraindication/intolerance), non‐invasive criteria guide endoscopic surveillance, and VBL is offered for medium to large varices. In decompensated cirrhosis with ascites, the international guidelines advise endoscopic surveillance and NSBB to be considered in patients with small varices and recommended for large varices.

In CALIBRE, individual decompensating events were analysed as secondary outcomes. There was no difference seen between the two treatment groups. A further exploratory analysis of the decompensation, which incorporated the individual events, was done (Appendix 1, Section 9). The BOPPP trial is being conducted in patients with cirrhosis and small varices and will provide more information in this regard [24].

In the carvedilol arm, 59 (46%) participants experienced recognised adverse events by the time of their safety visit, with 11% requiring discontinuation of carvedilol as a result. Since nearly 20% of participants in the carvedilol arm required the dose to be split or reduced due to adverse events, consideration should be given to starting carvedilol at 6.25 mg once a day to increase to 6.25 mg bd as tolerated, which aligns with current NICE guidance [19]. The safety profile is consistent with the literature. The use of carvedilol in portal hypertension remains off‐label, and recent NICE guidance has advised avoiding carvedilol in the setting of severe hepatic impairment, for example, those with large volume or refractory ascites [19].

Significant findings of this trial, not previously reported within an RCT, are the quality of life and health economic data. Existing data are limited to cost‐effectiveness analysis in the prevention of variceal bleeding outside of an RCT [10, 19]. Quality of life scores were similar in the two treatment arms during follow‐up, which suggests that despite the numerous side effects of carvedilol, there was no significant impact on quality of life. Health economic data show that carvedilol is cost‐saving and may be cost‐effective.

Previous qualitative research with patients alongside the CALIBRE pilot trial demonstrates no clear preference for NSBB or VBL [12]. Reflecting the trial data on adverse events and quality of life, patients allocated to carvedilol often noted side effects as a core aspect of their experience of treatment. Those allocated to VBL, but not carvedilol, tended to discuss the treatment positively, particularly as it was perceived to reassure patients that their varices had been treated effectively due to endoscopic follow‐up. The perceived effectiveness of treatments has been identified as a core component in the acceptability of healthcare interventions [25].

The main limitation of this trial is that it is underpowered, reaching 10% of the planned sample size for the reasons discussed earlier. The primary outcome is lower than in the previous UK trial [6]. This may reflect fewer patients with hepatic decompensation at baseline (24.2% vs. 59.2%) with shorter follow‐up of up to one versus 2 years, and a reduction in alcohol consumption during follow‐up. The small number of events and wide CIs could result in spare data bias. A combination of participants with compensated and decompensated patients at baseline is a potential limitation, but participants were stratified into these two stages of cirrhosis at baseline to facilitate analysis. Since most participants had compensated disease at baseline, we would advise caution in extrapolating the results to decompensated patients. Moreover, as the trial is UK‐based, generalisability would be limited in other healthcare systems. However, the trial remains one of the largest RCTs comparing carvedilol with VBL, with a sample size 70% higher than in the previous UK trial [6]. The adherence to treatment in the banding arm was low, with many patients crossing over to carvedilol, which may reflect the pandemic's impact on endoscopy services. However, analysis of the adherent population did not show any differences. The strengths include its multicentre pragmatic nature and completeness of data. The quality of life and health economic data contribute significantly to the literature.

In conclusion, the early terminated and, thus, underpowered CALIBRE trial found no difference between carvedilol 12.5 mg once daily and VBL in the primary prevention of variceal bleeding in participants with cirrhosis and medium to large‐size oesophageal varices. There were no untoward safety concerns, and both treatments were well tolerated, with similar effects on health‐related quality of life. Carvedilol is cost‐saving, potentially cost‐effective, and the preferred first‐line primary prevention option. Further large trials are recommended with longer follow‐up to investigate the role of carvedilol in the primary prevention of decompensation.

## Author Contributions

**Dhiraj Tripathi:** methodology, conceptualization, validation, investigation, funding acquisition, writing – review and editing, writing – original draft, supervision. **Kelly Handley:** conceptualization, methodology, software, formal analysis, validation, funding acquisition, project administration, writing – review and editing, writing – original draft, resources, supervision. **Lisa Holden:** methodology, conceptualization, software, data curation, formal analysis, project administration, resources, funding acquisition, writing – review and editing, writing – original draft. **Zainab Abdali:** methodology, software, data curation, validation, investigation, formal analysis, project administration, writing – review and editing, writing – original draft. **Sue Jowett:** conceptualization, methodology, software, data curation, supervision, formal analysis, validation, investigation, funding acquisition, resources, writing – review and editing, writing – original draft. **Jonathan Mathers:** conceptualization, methodology, software, data curation, investigation, validation, formal analysis, supervision, resources, funding acquisition, writing – review and editing, writing – original draft. **Chris Poyner:** conceptualization, methodology, software, data curation, formal analysis, validation, investigation, visualization, writing – review and editing, writing – original draft. **Paul Richardson:** conceptualization, methodology, investigation, funding acquisition, writing – review and editing. **James Ferguson:** writing – review and editing, conceptualization, methodology, investigation, funding acquisition. **Ian Rowe:** conceptualization, methodology, formal analysis, data curation, supervision, validation, investigation, funding acquisition, writing – review and editing, writing – original draft.

## CALIBRE Trial Collaborative Group

**Patient and public involvement** (Birmingham Liver Public and Patient Involvement Group, University Hospitals Birmingham NHS Foundation Trust, UK)**: Peter Devine:** conceptualisation, funding acquisition, methodology, project administration.

**Birmingham Clinical Trials Unit: Khaled Ahmed**: investigation, project administration. **Kate Brailsford**: data curation, project administration, resources, software, validation. **Peter Brocklehurst:** conceptualisation, funding acquisition, methodology, project administration, resources, supervision. **Margaret Grant:** conceptualisation, funding acquisition, methodology, project administration, resources, supervision. **Gemma Slinn:** conceptualisation, funding acquisition, methodology, project administration, resources, supervision. **Matthew Soden**: project administration, resources, supervision.

**Rebecca Amos‐Hirst**, **Hannah Bensoussane**, **Catherine Moakes:** data curation, formal analysis, resources, software, validation.

**Bhushan Chhajed**, **Paul Riley**, **Neil Winkles:** data curation, formal analysis, resources, software, validation.

**University of Birmingham: Lazaros Andronis**, **Christel McMullan:** conceptualisation, methodology, project administration.

**Data Monitoring Committee** (supervision)**: Martin Prince, Ann‐Marie Swart (Chair)**, **Hilary Watt**.

**Trial Steering Committee** (supervision)**: Varinder Athwal (Chair)**, **Harpreet Dhaliwal**, **Diarmaid Houlihan**, **Laura Mandefield**, **Nicholas Roslund**.

**Collaborator** (conceptualisation, funding acquisition, investigation, methodology, project administration)**:** Royal Infirmary of Edinburgh – *Peter Hayes.

**Collaborators** (investigation)**:** 1. Aberdeen Royal Infirmary – *Ashis Mukhopadhya, **Jennifer Cathcart, ***Pauline Dundas, ***Oluyemi Solanke, ***Siby Joseph. 2. University Hospital Aintree – *Cyril Sieberhagen, ***Melanie Harrison, ***Claire Burston. 3. Basildon – *Gavin Wright, ***Princess Gabiana, ***Aimee Williams, ***Bushena Miyesa. 4. Basingstoke & North Hampshire Hospital – *John Ramage, ***Hayley Cousins, ***Rachel Bryant. 5. Bedford Hospital – *Sim L Lim, *Janisha Patel, ***Mel Penacerrada, ***Rachel Lorusso. 6. Royal Victoria Hospital, Belfast – *Rory McCorry, ***Allison Lloyd, ***Heather Lawther. 7. Bournemouth Hospital – *Safa Al‐Shamma, ***Anna Maria Wilce. 8. Bradford Royal Infirmary – *Sulleman Moreea, ***Carol Firth. 9. Southmead Hospital, Bristol – *Robert Przemioslo, ***Charlotte Cranfield. 10. Addenbrookes Hospital, Cambridge – *Joanna Leithead, *Gwilym Webb, *Victoria Snowdon, **Aarani Mahalingam, ***Abigail Ford. 11. University Hospital of Wales, Cardiff – *Thomas Pembroke, ***Sarah Harrhy, ***Fiona Greaves. 12. Chelsea & Westminster Hospital – *Matthew Foxton, ***Carina Bautista. 13. University Hospitals Coventry and Warwickshire – *Esther Unitt, ***Susan Dale. 14. Royal Derby Hospital – *Andrew Austin, ***Catherine Addleton. 15. Russells Hall Hospital, Dudley – *Sharan Shetty, *Rizwan Mahmood, ***Clare Allcock, ***Karen Reid. 16. Ninewells Hospital, Dundee – *Michael Miller, ***Anne Elliott, ***Leanne Cosgrove, ***Shona Murray. 17. University Hospital of North Durham – *Francisco Porra Perez, ***Melanie Kent. 18. Royal Infirmary of Edinburgh – ***Marie Callaghan. 19. Queen Elizabeth Hospital, Gateshead – *Dina Mansour, ***Ann Wilson. 20. Glasgow Royal Infirmary – *Adrian Stanley, ***Alexis Duncan, ***Lauren Walker. 21. Gloucestershire Royal Hospital – *Coral Hollywood, ***Paula Hiltout. 22. Guy‘s & St Thomas Hospital – *Phil Berry, ***Jessica Cordle. 23. Birmingham Heartlands Hospital, University Hospitals Birmingham – *Andrew King, **Laurence Hopkins, ***Faye Moore, ***Mirriam Sangombe. 24. Hull Royal Infirmary – *Lynsey Corless, ***Tanis Nurun, ***Julie Wilcox, ***Bethia Featherstone. 25. Imperial College London – *Ameet Dhar, ***Maria Lanoria, ***Gary Hahn. 26. King‘s College Hospital, London – *Brian Hogan, *Mark McPhail, **Matthew M. Farrant, ***Nicola Griffiths, ***Ane Zamalloa. 27. Leeds General Infirmary – *Victoria Appleby, *Mark Aldersley, ***Rebecca Bishop, ***Louise Clark. 28. Leicester Royal Infirmary – *Farooq Khan, *Ka‐Ki Li, ***Olivia Watchorn. 29. Pinderfields Hospital – *John Hutchinson, ***Patricia Kane, ***Julie Burton, ***Stephanie Lupton, ***Jacqui Bartholomew, ***Emma‐Jane Stoner. 30. Freeman Hospital, Newcastle – *Steven Masson, ***Sarah Hogg. 31. Norfolk & Norwich University Hospital – *Simon Rushbrook, ***Vanessa Martin. 32. North Tyneside General Hospital – *Tom Lee, ***Jane Luke, ***Angela Green. 33. Queen‘s Medical Centre, Nottingham – *Stephen Ryder, ***Preethi Shibu. 34. John Radcliffe Hospital, Oxford – *Jeremy Cobbold, ***Loren Smith, ***Lou Holland, ***Eleni Rountenko. 35. Derriford Hospital, Plymouth – *Matthew Cramp, ***Alex Parkinson. 36. Queen Alexandra Hospital, Portsmouth – *Richard Aspinall, ***Emily Jones, ***Jincy Daniels, ***Anu Andrews. 37. Royal Cornwall Hospital – *Hyder Hussaini, *Lisa Trembath, ***Fiona Hammonds. 38. Royal Devon & Exeter Hospital – *Ben Hudson, ***Rob James. 39. Royal Free Hospital – *Raj Mookerjee, ***Christine Eastgate, ***Gloria Ferrariz, ***Abigail Brownlie. 40. Royal Glamorgan Hospital – *Dai Samuel, ***Bethan Deacon. 41. Royal Liverpool University Hospital – *Imran Patanwala, *Edward Britton, **Callum Wood, ***Martina Lofthouse, ***Giovanna Bretland. 42. Royal London Hospital – *Vikram Sharma, ***Louise Payaniandy, ***James Hand. 43. Salisbury Hospital – *Graham Branagan, *Aqueel Jamil, ***Alpha Anthony, ***Louise Bell. 44. Birmingham City Hospital and Sandwell General Hospital – *Ahmed Abu‐Shanab, ***Samia Hussain, ***Manjinder Gohel. 45. Scarborough General Hospital – *Charlie Millson, ***Kerry Elliott, ***Tania Nurun. 46. Sheffield Teaching Hospital – *Laura Harrison, *Barbara Hoeroldt, *Ihteshamul Haq, **Angela Liaros, ***Holly Caborn. 47. Royal Shrewsbury Hospital – *Ulrich Thalheimer, ***Kate Winstance. 48. South Tees – *Darren Craig, ***Julie Tregonning. 49. Southampton General Hospital – *Janisha Patel, **Helen Stone, ***Lijamol Joseph, ***Jisha Jacob. 50. South Tyneside District Hospital – *Joanne Topping, ***Peter Murphy, ***Jill Effard. 51. St George‘s Hospital – *Sarah Hughes, ***Criscel Jan Pelaez. 52. Sunderland Royal Hospital – *Rohit Sinha, ***Jenny Henderson. 53. Singleton Hospital, Swansea – *Jagdish Nagaraj, *Chin Lye Ch’Ng, ***Caradog Thomas, ***Elaine Brinkworth. 54. Great Western Hospital, Swindon – *Moby Joseph, *Andrew Claridge. 55. Torbay Hospital – *James Neale, ***Camille Walling, ***Maggie Ryder, ***Jacinta Ugoji. 56. Queen Elizabeth Hospital, Birmingham – *Neil Rajoriya, ***Emma Burke. 57. Walsall Manor Hospital – *Nishant Gautam, **Julie Fletcher. 58. Royal Albert Edward Infirmary, Wigan – *Richard Keld, ***Philip Paterson. 59. Newcross Hospital, Wolverhampton – *Christopher Corbett, ***Marie Green. 60. York Hospital – *Charlie Millson, ***Siobhan Sutton, ***Naomi Gott.

*Represents the Local Principal Investigator. **Represents the Local Associate Principal Investigator(s). ***Represents the Local Senior Research Nurse(s).

## Disclosure

Information Governance Statement: The University of Birmingham is committed to handling all personal information in line with the UK Data Protection Act (2018) and the General Data Protection Regulation (EU GDPR) 2016/679. Under the Data Protection legislation, the University of Birmingham is the Data Controller, and you can find out more about how we handle personal data, including how to exercise your individual rights and the contact details for our Data Protection Officer here (https://www.birmingham.ac.uk/research/bctu/data‐sharing‐and‐protection‐policy).

## Ethics Statement

The trial was approved by a National Health Service (NHS) Research Ethics Committee (REC, reference number 18/NE/0296) on 19 October 2018.

## Conflicts of Interest

Dhiraj Tripathi is the principal investigator of drug therapy trials in portal hypertension sponsored by Boehringer Ingelheim and AstraZeneca (payments to institution). Dhiraj Tripathi is the principal investigator of Beta‐blockers or placebo for primary prophylaxis of oesophageal varices (BOPPP study) funded by the National Institute for Health and Care Research (NIHR) HTA programme (payments to institution).

## Supporting information


Data S1:

## Data Availability

All data requests should be submitted to the corresponding author. Access to anonymised data may be granted following review. A Data Sharing agreement may be required before data can be released.
