# Supplementary material for: Clinical Trial: A Multicentre Randomised Controlled Trial of Carvedilol Versus Variceal Band Ligation in Primary Prevention of Variceal Bleeding in Liver Cirrhosis (CALIBRE Trial)
Source: Aliment Pharmacol Ther. 2025 Apr 16;61(11):1740–54. doi: 10.1111/apt.70080 (PMC12074564; doi:10.1111/apt.70080)
Supplement: Supplementary file 1 — Data S1: [file APT-61-1740-s001.zip › apt70080-sup-0004-CALIBRE HEAP_V2.pdf]

Multi-centre randomised trial to compare Carvedilol vs. variceal band ligation for the primary prevention of variceal bleeding in liver cirrhosis: The CALIBRE Trial

Health Economic Analysis Plan (HEAP)

Contents

Section 1: Administrative Information **4**

Section 2: Introduction **6**

2.1 Trial background and rational 6

2.2 Objectives of the trial 6

Section 3: Economic Approach **7**

3.2 Objectives of economic evaluation 7

3.3 Overview of Economic Analysis 7

3.4 Jurisdiction 8

3.5 Perspectives 8

3.6 Time horizon 8

Section 4: Trial based analysis - Economic Data Collection and Management **9**

4.1 Statistical software use for health economic analysis 9

4.2 Resource use and cost 9

4.3 Health outcomes 9

Section 5: Trial based analysis - Data Analysis **10**

5.1 Analysis population 11

5.2 Timing of analyses 11

5.3 Discount rates for costs and benefits 11

5.4 Cost-effectiveness threshold(s) 11

5.6 Analysis of resource use 11

5.7 Analysis of costs 11

5.8 Analysis of outcomes 12

5.9 Data cleaning for analysis 12

5.10 Missing data 12

5.11 Analysis of cost-effectiveness 12

5.12 Sampling uncertainty 12

5.13 Subgroup analyses/Analysis of heterogeneity 13

5.14 Sensitivity Analyses 13

Section 6: Model based health economic analysis **13**

6.1 Decision analytic modelling **14**

6.2 Model type **14**

6.3 Model structure ..... Error! Bookmark not defined.

6.4 Treatment effect beyond the end of the trial ..... Error! Bookmark not defined.

6.5 Key assumptions.. 14
6.6 Methods for identifying and estimating parameters 14
6.7. Model parameters .... Error! Bookmark not defined.
6.7.1 Probabilities .... **Error! Bookmark not defined.**
6.7.2 Resource use and costs .... **Error! Bookmark not defined.**
6.7.3 Effectiveness/utilities.. **Error! Bookmark not defined.**
6.8 Model uncertainty .... Error! Bookmark not defined.
6.9 Subgroup analyses/ Heterogeneity.. 15
Section 7: Reporting/Publishing 16
7.1 Reporting standards 16
7.2 Reporting deviations from the HEAP 16
Health economics suggested tables and figures. 17
Trial based analysis. .... Error! Bookmark not defined.
References:.. 23

Section 1: Administrative Information

Title	Multi-centre randomised trial to compare Carvedilol vs. variceal band ligation for the primary prevention of variceal bleeding in liver cirrhosis: The CALIBRE Trial
Trial registration number; registry	ISRCTN 73887615
Source of funding	The National Institute of Health Research (NIHR), Health Technology Assessment (HTA) Programme is funding this trial under reference 16/99/02.
Purpose of HEAP	The purpose of this HEAP is to describe the analysis and reporting procedure intended for the economic analyses to be undertaken. The plan is designed to ensure that there is no conflict with the protocol and associated statistical analysis plan (SAP) and highlight potential issues at an early stage to be discussed with the trial team. It is to be read in conjunction both the protocol and SAP. Any deviations from this plan will be justified in the final report/study. The health economic analysis plan is designed as a working document that will evolve throughout data collection, cleaning, and preliminary analysis.
Trial protocol version; date	This document has been written based on the information contained in the trial protocol Version 3.0, 7th September 2021.
Trial Statistical Analysis Plan (SAP) version, date	SAP version 1.0, Date: 23/10/2019
Trial HEAP version, date	HEAP Version: 2.0, Date: 02/02/2024
HEAP revisions	
Roles and responsibilities	This HEAP was prepared by Zainab Abdali (research fellow in health economics) and approved by Professor Sue Jowett. The trial health economist (Zainab Abdali) is responsible for conducting and reporting the economic evaluation analysis in accordance with the HEAP and Sue Jowett will supervise the analysis.

APPROVALS
The following people have reviewed the Health Economics Analysis Plan and are in agreement with the contents.

**Role**	**Name**	**Signature**	**Date**

Author	Zainab Abdali		2/02/2024
Lead Health Economist	Professor Sue Jowett		
Chief Investigator	Professor Dhiraj Tripathi		

Section 2: Introduction

2.1 Trial background and rational

In brief, liver disease is the 5th largest cause of death in the UK, with mortality predicted to double in 20 years. Patients with liver disease die younger with the average age of death of 59 years, compared with 82-84 years for heart and lung disease and stroke. In England, in total 30,000–60,000 patients are at risk or affected by liver cirrhosis. One of the major complications of cirrhosis is portal hypertension and variceal bleeding. In patients with cirrhosis, varices develop at a rate of 5% per year with 10-year cumulative incidence of 44%. At least 3,000 patients are admitted to hospital in England per year with variceal bleeding, with inpatient mortality of 15%- and one-year mortality of up to 40%.

At present there are two options for primary prevention of variceal bleeding, namely non-selective beta-blockers and variceal band ligation. There have been two important guidelines published in the UK in 2015-2016 from NICE and the British Society of Gastroenterology (BSG)1-2. NICE favours banding for primary prevention, whereas the British Society of Gastroenterology (BSG) suggests banding if intolerant of beta-blockers. A large randomised controlled trial would help clinicians decide which the better treatment for primary prevention is as the current evidence is based on underpowered and low-quality trials. Beta-blockers used for portal hypertension in the UK are propranolol and carvedilol. Carvedilol is better tolerated and therefore has been selected as the beta-blocker for this trial. If carvedilol is found to be superior to variceal band ligation, then it will become first line therapy in primary prevention.

2.2 Objectives of the trial

The primary objective of the trial is to compare carvedilol versus variceal band ligation (VBL) with respect to variceal bleeding within 1 year of randomisation in participants with cirrhosis and medium to large oesophageal varices that have never bled.

Section 3: Economic Approach

3.1 Objectives of economic evaluation

Evidence on cost effectiveness of carvedilol in the context of preventing the first variceal bleeding is available from one study.[1] The study found that using beta-blockers resulted in lower overall costs (including treatment, follow-up, and bleeding-related costs) compared to the use of variceal band ligation. NICE, updated cirrhosis guidelines included a 1-year decision model (developed by NICE) in the form of cost-utility analysis to evaluate the cost-effectiveness of endoscopic variceal band ligation (EVL) versus non-selective beta-blockers (NSBBs)[2]. The model showed that, compared to NSBBs, EVL was not cost-effective due to its high cost with little impact on quality of life. Due to insufficient evidence on adverse events, management costs of adverse events were not considered in the model.

This health economic analysis aims to assess the short- and long-term cost-effectiveness of carvedilol versus variceal band ligation (VBL) in patients with cirrhosis and medium to large oesophageal varices that have never bled.

3.2 Overview of economic analysis

Within-trial cost-effectiveness and cost-utility analyses will be conducted from a National Health Service and Personal Social Services (NHS/PSS) perspective, as per recommended guidelines[3] based on the outcomes of cost per variceal bleeding avoided within one year of randomisation and cost per Quality-adjusted Life-year (QALY). Prevention of variceal bleeding is the primary outcome of the clinical trial, and therefore it was important to evaluate the cost-effectiveness of carvedilol based on this outcome. As recommended by the National Institute for Health and Care Excellence (NICE)[3] EQ-5D-5L measure will be used to estimated QALYs to enable comparison of interventions across different health conditions.

An incremental cost-effectiveness ratio (ICER) will be calculated for each analysis:

$$ICER = \frac{\text{Cost (TREAT intervention)} - \text{Cost (Usual care)}}{\text{QALYs(TREAT intervention)} - \text{QALYs (Usual care)}}$$

The analyses will use patient-level data on resource use and effectiveness collected prospectively at different follow up points.

If a clinical difference between strategies is found within the trial, a decision model-based economic evaluation will be conducted to evaluate the long-term costs and outcomes beyond the trial period and proposed methods are reported in section 6.

3.3 Jurisdiction

The trial will be conducted in the UK which has a National Health Service (NHS), providing publicly funded healthcare, primarily free of charge at the point of use.

3.4 Perspectives

In line with NICE recommendations[3], the base-case analysis will adopt a health care system (payer's) perspective where costs incurred by the NHS.

3.5 Time horizon

For the trial-based economic evaluation, the cost-effectiveness and cost-utility of carvedilol and VBL will be compared over 12 months. A 5-year decision model is planned to evaluate the long-term cost-effectiveness of the interventions.

Section 4: Trial-based analysis - Economic Data Collection and Management

4.1 Statistical software use for health economic analysis

Stata SE version 17 will be used for the exploratory and statistical analyses. [4]

4.2 Resource use and cost

4.2.1 Identification of resources

Information on healthcare resource use required to deliver the interventions (carvedilol or variceal band ligation) will be collected within the trial. Individual-level hospital-based service use associated with the treatment of variceal bleeding and follow-up care will also be collected including inpatient hospital stay (admission and readmissions) in general ward settings or intensive care units, treatment of adverse events and other complications such as ascites, spontaneous bacterial peritonitis, renal dysfunction, hepatocellular carcinoma, and hepatic encephalopathy. Primary healthcare services related to GP appointments will also be obtained.

4.2.2 Measurement of resource use data

Data on health care resource use will be collected prospectively for each participant at 6 and 12 months follow-up visit using a case report form (CRF) so that a stochastic cost analysis can be undertaken. Where the same health resource is collected using different forms, data will be compared to avoid double counted activity.

4.2.3 Valuation of resource use data

The cost of variceal band ligation will be sourced from the NICE guidance[2], and the BNF[5] will be used for the cost of carvedilol or any other/alternative prescribed medications. Unit costs of health care and social services resource use will be obtained from national sources including NHS reference costs and PSSRU. [6,7]

4.3 Health outcomes

4.3.1 Identification of outcomes

The primary outcome of the cost-effectiveness analysis will be based on the primary outcome of the trial which is variceal bleeding within 12 months of randomisation. For the cost-utility analysis, the

outcome measure will be QALYs over a 12-month period. The outcome combines both length and quality of life where one QALY represents one year of life in perfect health.

4.3.2 Measurement of outcomes

Patients' health related quality of life will be measured using EQ-5D-5L, which is a generic measure of health related quality of life (HRQoL), recommended by NICE.[8] This measure is used to capture changes in 5 dimensions of health (mobility, self-care, usual activities, pain and discomfort, and anxiety and depression) and each dimension has 5 levels (1=no problems to 5=extreme problems/unable to perform). Participants will complete EQ-5D-5L questionnaire at baseline, 6 and 12 months.

4.3.3 Valuations of outcomes

Following NICE recommendation for health technology appraisal[8], EQ-5D-5L individual responses will be converted to utility scores using the mapping function developed by the Decision Support Unit (DSU)[9] . This method maps EQ-5D-5L responses onto the EQ-5D-3L tariff in order to generate utility scores. The trapezoidal method will be used to generate QALYs by combining EQ-5D-5L preference-based utility values with time. [10] The crosswalk method[11] will also be used to generate utility values, which will be presented as part of the sensitivity analysis.

.

Section 5: Trial-based analysis - Data Analysis

5.1 Analysis population

In line with the statistical analysis, the health economic analysis will be based on the intention-to-treat (ITT) principle. All randomised patients will be considered in the analysis according to the allocated intervention at randomisation.

5.2 Timing of analyses

12 months period

5.3 Discount rates for costs and benefits

Given that the last follow up point is 12 months, discounting is not required.

5.4 Cost-effectiveness threshold(s)

There is no defined cost-effectiveness threshold in the UK for the primary outcome (variceal bleeding). For the cost-utility analysis, NICE's upper and lower thresholds of £30,000 and £20,000 per QALY[13] will be used to determine if an intervention is deemed to cost-effective.

5.5 Analysis of resource use

The mean per participant, standard deviations (SD), maximum and minimum values of healthcare resource use at 12-months will be reported by intervention group.

5.6 Analysis of costs

Initially mean disaggregated costs by cost category (trial intervention, hospital stay and other hospital-based services and primary healthcare services) will be presented to provide indication of resource utilisation. Total aggregated mean NHS costs at 12 months will be presented for both treatment arms and used in the final analysis. Given that cost data are likely to be skewed as a result of few patients with high resource use costs, mean differences between interventions with 95%

confidence intervals (CIs) will be estimated using a non-parametric bootstrap approach and adjusted for baseline characteristics.

5.7 Analysis of outcomes

For the EQ-5D-5L measure, mean utility score values and bootstrapped mean differences (adjusted for baseline characteristics) between treatment arms will be reported at the different follow up points (baseline, 6 and 12 months). Mean (SD) QALYs at 12-months will also be reported with the bootstrapped adjusted mean difference between carvedilol and VBL.

5.8 Data cleaning for analysis

We will liaise with trial statisticians in identifying issues with data such as miscoding. Data cleaning will include face validity tests conducted on data (e.g., to identify misspelt text) which will be checked against the source documents. Any remaining uncertainties will be addressed by health economists and referred to clinical experts if necessary for adjudication. All corrections will be documented in the Stata do file.

5.9 Missing data

The level of missingness over the trial period will be explored to see how many values are missing at different follow up points. Depending on the type and level of missingness, missing data will be accounted for using and appropriate technique such as multiple imputation.[14]

5.10 Analysis of cost-effectiveness

Differences between treatment arms in total costs will be divided by differences in effectiveness (Variceal bleeding and QALYs) to calculate Incremental Cost Effectiveness Ratios (ICER), expressed as the additional cost per variceal bleeding avoided and cost per QALY gained.

5.11 Sampling uncertainty

Non-parametric bootstrapping will be used to account for the overall uncertainty that occurs because of variations in sampling, by jointly bootstrapping mean cost and outcome measure

differences. The technique will generate 5,000 paired values of incremental costs and variceal bleeding avoided, death avoided and QALYs. The values will be presented on a cost effectiveness plane as a scatterplot to aid interpretation.

5.12 Subgroup analyses/Analysis of heterogeneity

A planned subgroup analysis will take place to explore the relative cost-effectiveness for prespecified subgroups of interest.

5.13 Sensitivity Analyses

Several scenario analyses will be undertaken to explore the cost-effectiveness of the interventions using complete cost and effectiveness data, alternative assumptions and explore the broader issue of the generalisability of the results.

Section 6: Model based health economic analysis

6.1 Decision analytic modelling

If there is evidence, from the trial that differences between carvedilol and variceal band ligation exist, in terms of re-bleeding or mortality rates as well as other outcomes that may have significant cost or outcome implications beyond the trial period, a decision model-based economic evaluation will additionally be conducted. This will ensure that all important costs and benefits are taken into account in economic analysis.

Depending on data availability, the analysis will be conducted from the NHS/PSS perspective, using a 5-year time horizon and recommended discounting adjustments. Deterministic and probabilistic sensitivity analyses will be conducted to explore the robustness of the results.

6.2 Model type

The model is expected to be a Markov model.

6.3 Health states and model assumptions

To be confirmed.

6.4 Methods for identifying and estimating parameters

Good practice guidelines will be followed for identifying and estimating parameters.

The effectiveness of VBL intervention and carvedilol will be obtained from the trial. Adjustments will be made as determined in the statistical analysis plan. Other long-term parameters (effectiveness, outcomes, and costs) will be taken from the literature or assumptions if not collected in the study.

6.5 Model uncertainty

Extensive deterministic one-way sensitivity analyses are planned to explore which model parameters are likely to affect the cost-effectiveness. The results of these analysis will be presented in a tornado diagram. Methodological and structural uncertainty will be explored in scenario analyses to estimate the impact of model assumptions on the final findings.

A probabilistic sensitivity analysis (PSA) with 10,000 replications of mean ICERs will be conducted to explore the uncertainty of the model input data by varying all relevant parameters. Parameters will be incorporated into the model as distributions and have. Beta distribution will be applied to probabilities, while gamma distribution will be used to resource use and costs. The generated 10,000 simulations representing the model outputs will be presented as scatterplots on the cost-effectiveness plane. Cost-effectiveness acceptability curves will be used to reflect sampling variation and uncertainties in the appropriate threshold cost-effectiveness values.

6.6 Subgroup analyses/ Heterogeneity

To be confirmed.

Section 7: Reporting/Publishing

7.1 Reporting standards

CHEERS guidelines[15] will be followed when reporting the health economic evaluation, in a format that is appropriate to policymakers and stakeholders. The Phillips checklist will be followed when reporting the model-based economic evaluation for journal publication. [16]

7.2 Reporting deviations from the HEAP

Any deviation from HEAP will be described and justified in the final published report. Circumstances under which changes will be permitted are as follows:

- development of statistical methods that are deemed more appropriate for the analysis.
- preliminary data cleaning or analysis suggesting that planned analyses may require amendment

Health economics suggested tables and figures.

Table 1: Resource use unit cost – £ 2023 prices

	Unit cost	Description	Source
**Intervention**			
Carvedilol 12.5mg (per tablet)	£0.05	£1.32 Pack of 28 tablets<br>Once daily dose of 12.5mg	BNF 2022
Variceal band ligation	£2,243	4 sessions price 2019/2020<br>Price per session is £561	NICE guidelines /model source was an NHS hospital trust a member worked in
**Secondary care (hospital based) services¥**			
**Hospital stays (per day)**			
General ward			
ITU			
**Outpatient visit**			
Outpatient consultant visit	£184	Weighted average of consultant led outpatient attendance	
**Diagnostic procedure/imaging or test (per visit)**			
Endoscopy	540	FE22Z (general diagnostic Endoscopic Upper Gastrointestinal Tract Procedures, 19 years and over) – outpatient procedure - 5,407 procedures	NHS reference cost
Endoscopy	£597	FE22Z (general diagnostic Endoscopic Upper Gastrointestinal Tract Procedures, 19 years and over) – day case – 150,269 procedures	
Ultrasound	£69	Ultrasound Scan with duration of less than 20 minutes, without Contrast	
Fibroscan	£88	Ultrasound Elastography (RD48Z)	
ECG			

	Unit cost	Description	Source
**Primary care (community-based) services**			
GP visit	£41	9.22 minutes contact including direct care staff cost with qualification	PSSRU 2022

Table 2: Mean NHS services resource use and costs by intervention group

Resource use	VBL n=		Carvedilol n=		Mean adjusted bootstrapped difference in £ costs (95% CI)
	Mean unit (SD)	Mean Cost £ (SD)	Mean unit (SD)	Mean Cost £ (SD)	
**Trial interventions**					
**Secondary (hospital-based) healthcare services**					
Hospital stay (days)					
Outpatient visit					
Diagnostic imaging/tests					
Ultrasound					
Fibroscan					
ECG					
**Primary healthcare services**					
GP at practice					

Table 3: Mean EQ-5D-5L score per participant by intervention group at different follow up points

Follow up point	VBL<br>n=<br>Mean score (SD)	Carvedilol<br>n=<br>Mean score (SD)	Mean adjusted bootstrapped difference (95% CI)
Baseline			
n			
Mean (SD)			
2 weeks			
n			
Mean (SD)			
6 months			
n			
Mean (SD)			
12 months			
n			
Mean (SD)			

Table 4: Health economic evaluation base case results (ITT) for the trial-based and model-based analyses from the perspective of the UK-NHS.

Analysis	Mean total £cost	Incremental £cost	Mean outcome	Incremental outcome	ICER (£cost per outcome)
**Trial- based EE, 12 months after randomisation**					
**CEA - Variceal bleeding avoided**					
VEL					
Carvedilol					
**CUA - Quality Adjusted Life Year (QALY)**					
VEL					
Carvedilol					
**Model-based EE, 5 years**					
**CEA- Variceal Bleeding avoided**					
VEL					
Carvedilol					
**CUA - Quality Adjusted Life Year (QALY)**					
VEL					
Carvedilol					

Table 5: Model input parameters

	Description	Base case values	Distribution	Range	Source
Probabilities					
Bleeding					
Re-bleeding					
Develop Varices					
Death					
Utilities					
Costs					

References:

1. Norberto L, Polese L, Cillo U, et al. A randomized study comparing ligation with propranolol for primary prophylaxis of variceal bleeding in candidates for liver transplantation. *Liver Transplantation* 2007; **13**(9): 1272-8.
2. NICE. Cirrhosis in over 16s: assessment and management (update). 2023 https://www.nice.org.uk/guidance/ng50/evidence/a-clinical-and-costeffectiveness-of-nonselective-betablockers-and-endoscopic-variceal-band-ligation-for-the-primary-prevention-of-bleeding-in-people-with-oesophageal-varices-due-to-cirrhosis-pdf-13181683358.
3. National Institute for Health and Care Excellence (NICE). Guide to the Methods of Technology Appraisal 2013. Process and Methods [PMG9]. . www.nice.org.uk/article/pmg9/chapter/foreword (accessed 28 February 2023.
4. StataCorp L. Stata statistical software: release 17 College Station. *TX StataCorp LP* 2021.
5. British National Formulary (BNF). Available at: https://bnf.nice.org.uk/drugs
6. NHS National Schedule of Reference Costs 2019/20. URL: https://www.england.nhs.uk/publication/2019-20-national-cost-collection-data-publication/. (accessed 1 March 2023.
7. Jones KC, Weatherly H, Birch S, et al. Unit Costs of Health and Social Care 2022 Manual. 2022.
8. National Institute for Health Care Excellence. NICE health technology evaluations: the manual. 2022. www.nice.org.uk/process/pmg36
9. Hernández Alava M, Pudney S, Wailoo A. Estimating the relationship between EQ-5D-5L and EQ-5D-3L: results from a UK population study. *Pharmacoeconomics* 2023; **41**(2): 199-207.
10. Drummond MF, Sculpher MJ, Claxton K, Stoddart GL, Torrance GW. Methods for the economic evaluation of health care programmes: Oxford university press; 2015.
11. Van Hout B, Janssen M, Feng Y-S, et al. Interim scoring for the EQ-5D-5L: mapping the EQ-5D-5L to EQ-5D-3L value sets. *Value in health* 2012; **15**(5): 708-15.
12. National Institute for Health and Care excellence (NICE). Position statement on use of the EQ-5D-5L value set for England (updated October 2019). Available at: Position statement on use of the EQ-5D-5L value set for England (updated October 2019) | Technology appraisal guidance | NICE guidance | Our programmes | What we do | About | NICE. (accessed on February 3, 2021).
13. National Institute for Health and Care Excellence (NICE). Guide to the Methods of Technology Appraisal 2013. Process and Methods [PMG9]. Available at: www.nice.org.uk/article/pmg9/chapter/foreword (accessed 1st of February 2021).
14. Burton A, Billingham LJ, Bryan S. Cost-effectiveness in clinical trials: using multiple imputation to deal with incomplete cost data. *Clinical trials* 2007; **4**(2): 154-61.
15. Husereau D, Drummond M, Augustovski F, et al. Consolidated health economic evaluation reporting standards (CHEERS) 2022 explanation and elaboration: a report of the ISPOR CHEERS II good practices task force. *Value in health* 2022; **25**(1): 10-31.
16. Philips Z, Ginnelly L, Sculpher M, et al. Review of guidelines for good practice in decision-analytic modelling in health technology assessment. *Health technology assessment (Winchester, England)* 2004; **8**(36): iii-iv, ix.
