# Supplementary material for: Clinical Trial: A Multicentre Randomised Controlled Trial of Carvedilol Versus Variceal Band Ligation in Primary Prevention of Variceal Bleeding in Liver Cirrhosis (CALIBRE Trial)
Source: Aliment Pharmacol Ther. 2025 Apr 16;61(11):1740–54. doi: 10.1111/apt.70080 (PMC12074564; doi:10.1111/apt.70080)
Supplement: Supplementary file 1 — Data S1: [file APT-61-1740-s001.zip › apt70080-sup-0005-CALIBRE SAP v1.0.pdf]

UNIVERSITY OF BIRMINGHAM | BCTU Birmingham Clinical Trials Unit

# Multi-centre randomised trial to compare Carvedilol vs. variceal band ligation for the primary prevention of variceal bleeding in liver cirrhosis: The CALIBRE Trial

CALiBRE

Trial Registration: ISRCTN **73887615**

# Appendix 2: Statistical Analysis Plan

| SAP Version Number | Protocol Version Number |
|---|---|
| 1.0 | 1.0 |

| | | | | | |
|---|---|---|---|---|---|
| Author: | Hannah Bensoussane | Role: | Trial Statistician | Affiliation: | BCTU<br>University of Birmingham |
| Signature: | | Date: | | | |
| Chief Investigator: | Dhiraj Tripathi | Role: | Chief Investigator | Affiliation: | University of Birmingham |
| Signature: | | Date: | | | |
| **This Statistical Analysis Plan has been approved by:** | | | | | |
| Approver: | Kelly Handley | Role: | Senior Statistician | Affiliation: | BCTU<br>University of Birmingham |
| Signature: | | Date: | | | |

## Statistical Analysis Plan Amendments

| SAP version number | Date Approved | Protocol version number† | Section number changed | Description of and reason for change | Timing of change with respect to interim/final analysis | Blind Reviewer | |
|---|---|---|---|---|---|---|---|
| | | | | | | Name: | |
| | | | | | | Signature: | |
| | | | | | | Date: | |
| | | | | | | Name: | |
| | | | | | | Signature: | |
| | | | | | | Date: | |
| | | | | | | Name: | |
| | | | | | | Signature: | |
| | | | | | | Date: | |

† This SAP was written based on information contained in the trial protocol version as listed here.

| Abbreviations & Definitions | |
|---|---|
| **Abbreviation / Acronym** | **Meaning** |
| BCTU | Birmingham Clinical Trials Unit |
| BMI | Body Mass Index |
| BSG | British Society of Gastroenterology |
| CONSORT | Consolidated Standards of Reporting Trials |
| CRF | Case Report Form |
| CTCAE | Common Terminology Criteria for Adverse Events |
| DMC | Data Monitoring Committee |
| ICA-AKI | International Club of Ascites - Acute Kidney Injury |
| ISRCTN | International Standard Randomised Controlled Trial Number |
| ITT | Intention to Treat |
| QALY | Quality-adjusted Life Year |
| SAE | Serious Adverse Event |
| SAP | Statistical Analysis Plan |
| SUSAR | Suspected Unexpected Serious Adverse Reaction |
| TSC | Trial Steering Committee |
| **Term** | **Definition** |
| International Standard Randomised Controlled Trial Number | A clinical trial registry |
| Protocol | Document that details the rationale, objectives, design, methodology and statistical considerations of the study |
| Randomisation | The process of assigning trial participants to intervention or control groups using an element of chance to determine the assignments in order to reduce bias. |
| Statistical Analysis Plan | Pre-specified statistical methodology documented for the trial, either in the protocol or in a separate document. |

# TABLE OF CONTENTS


1. Introduction ... 7
2. Background and rationale ... 7
3. Trial objectives ... 8
4. Trial methods ... 8
4.1. Trial design ... 8
4.2. Trial interventions ... 8
4.3. Primary outcome measure ... 9
4.4. Secondary outcome measures ... 9
4.5. Timing of outcome assessments ... 9
4.6. Randomisation ... 10
4.7. Sample size ... 10
4.8. Framework ... 10
4.9. Interim analyses and stopping guidance ... 9
4.10. Internal Pilot Progression Rules ... 10
4.11. Timing of final analysis ... 10
4.12. Timing of other analyses ... 10
4.13. Trial comparisons ... 10
5. Statistical Principles ... 11
5.1. Confidence intervals and p-values ... 11
5.2. Adjustments for multiplicity ... 11
5.3. Analysis populations ... 11
5.4. Definition of adherence ... 11
5.5. Handing protocol deviations and violations ... 12
5.6. Unblinding ... 12
6. Trial population ... 12
6.1. Recruitment ... 12
6.2. Baseline characteristics ... 12
7. Intervention(s) ... 12
7.1. Description of the intervention(s) ... 12
7.2. Adherence to allocated intervention ... 13
8. Protocol deviations and violations ... 13
9. Analysis methods ... 13
9.1. Covariate adjustment ... 13
9.2. Distributional assumptions and outlying responses ... 13
9.3. Handling missing data ... 14
9.4. Data manipulations ... 14
9.5. Analysis methods – primary outcome(s) ... 18
9.6. Analysis methods – secondary outcomes ... 19
9.7. Analysis methods – exploratory outcomes and analyses ... 19
9.8. Safety data ... 20
9.9. Planned subgroup analyses ... 20
9.10. Sensitivity analyses ... 20
10. Analysis of sub-randomisations ... 21
11. Health economic analysis ... 21
12. Statistical software ... 21
13. References ... 21
Appendix A: Deviations from SAP ... 23
Appendix B: Trial schema ... 24
Appendix C: Schedule of assessments ... 25

**Appendix D: Template report** ........................................................................................................ 26

## 1. Introduction

This document is the Statistical Analysis Plan (SAP) for the CALIBRE trial, and should be read in conjunction with the current trial protocol. This SAP details the proposed analyses and presentation of the data for the main paper(s) reporting the results for the CALIBRE trial.

The results reported in these papers will follow the strategy set out here. Subsequent analyses of a more exploratory nature will not be bound by this strategy, though they are expected to follow the broad principles laid down here. The principles are not intended to curtail exploratory analysis (e.g. to decide cut-points for categorisation of continuous variables), nor to prohibit accepted practices (e.g. transformation of data prior to analysis), but they are intended to establish rules that will be followed, as closely as possible, when analysing and reporting data.

Any deviations from this SAP will be described and justified in the final report or publication of the trial (using a table as shown in Appendix A). The analysis will be carried out by an appropriately qualified statistician, who should ensure integrity of the data during their data cleaning processes.

## 2. Background and rationale

The background and rationale for the trial are outlined in detail in the protocol. In brief, liver disease is the 5th largest cause of death in the UK, with mortality predicted to double in 20 years. Patients with liver disease die younger with the average age of death of 59 years, compared with 82-84 years for heart and lung disease and stroke. In England, in total 30,000–60,000 patients are at risk or affected by liver cirrhosis. One of the major complications of cirrhosis is portal hypertension and variceal bleeding. In patients with cirrhosis, varices develop at a rate of 5% per year with 10 year cumulative incidence of 44%[1]. At least 3,000 patients are admitted to hospital in England per year with variceal bleeding, with inpatient mortality of 15% and one year mortality of up to 40%.

At present there are two options for primary prevention of variceal bleeding, namely non-selective beta-blockers and variceal band ligation. There have been two important guidelines published in the UK in 2015-2016 from NICE and the British Society of Gastroenterology (BSG)[1-2]. NICE favours banding for primary prevention, whereas the British Society of Gastroenterology (BSG) suggests banding if intolerant of beta-blockers. A large randomised controlled trial would help clinicians decide which the better treatment for primary prevention is as the current evidence is based on underpowered and low quality trials. Beta-blockers used for portal hypertension in the UK are propranolol and carvedilol. Carvedilol is better tolerated and therefore has been selected as the beta-blocker for this trial. If carvedilol is found to be superior to variceal band ligation then it will become first line therapy in primary prevention.

# 3. Trial objectives

The primary objective is to compare carvedilol versus variceal band ligation with respect to variceal bleeding within 1 year of randomisation in participants with cirrhosis and medium to large oesophageal varices that have never bled.

Secondary objectives are as follows:

- To investigate the effect of carvedilol and variceal band ligation on survival, development of other complications of cirrhosis and adverse events.
- To assess cost-effectiveness, participant preference and use of alternative or cross over therapies.

# 4. Trial methods

## 4.1. Trial design

CALIBRE is a multicentre, pragmatic, randomised controlled, open-label, two arm parallel group trial with an internal pilot. Approximately 66 Acute NHS Trust/Health Boards in the UK will be open to recruitment. Participants will be recruited from secondary care in one of three ways; in routine variceal surveillance endoscopy; referral from an outpatient clinic following a diagnostic endoscopy; and identification from inpatient referrals. See Appendix B for trial schema.

Due to the very different nature of the two interventions under study (drug versus endoscopic variceal band ligation), it is not feasible to have a blinded design. There are treatment implications for the participants following their allocated procedure and therefore the research staff need to be aware of the intervention received.

## 4.2. Trial interventions

*Carvedilol*

Participants will be prescribed 12.5mg once daily (od) for up to 12 months. They will be seen in a follow up clinic at four weeks to assess for any short term adverse events such as symptomatic hypotension, gastrointestinal side effects like nausea, and shortness of breath[1]. These participants will not be offered routine endoscopic surveillance, as per standard of care[1].

*Variceal Band Ligation*

The procedure will be performed as per the British Society of Gastroenterology guidelines[1]. Varices are banded at regular intervals (usually two to four weekly) until they are eradicated when they are normally replaced by scar tissue or by varices of much smaller size. Participants usually require on average 2-3 banding procedures to achieve eradication. After successful eradication of the varices, participants have a repeat endoscopy at approximately three

months, then at approximately six monthly thereafter. Any recurrent varices (i.e. medium to large varices) are treated with further variceal band ligation until eradication and then offered repeat endoscopy at approximately six monthly intervals.

## 4.3. Primary outcome measure

The primary outcome is any variceal bleeding within 1 year of randomisation.

## 4.4. Secondary outcome measures

The secondary outcomes are as follows:

- Time to first variceal bleed in days (from randomisation).
- Mortality at one year (from randomisation):
    - All-cause mortality
    - Liver related mortality
    - Cardiovascular mortality.
- Transplant free survival at one year (from randomisation).
- Adverse events related to treatment (up to 12 months after randomisation):
    - Dysphagia requiring discontinuation of treatment
    - Symptomatic hypotension requiring change in treatment
    - Dyspnoea
    - Gastrointestinal upset.
- Other complications of cirrhosis:
    - New onset ascites confirmed clinically or on imaging and graded as per ICA recommendations[3].
    - New onset encephalopathy defined using West Haven Criteria[4].
    - Spontaneous bacterial peritonitis
    - Hepatocellular carcinoma
    - Any renal dysfunction as per International Club of Ascites – Acute Kidney Injury (ICA-AKI) definitions[5].
- Health-related quality of life (EQ-5D-5L) from randomisation to six and 12 months.
- Use of healthcare resources, costs and cost-effectiveness based on the outcomes of cost per variceal bleeding avoided within one year of randomisation, cost per Quality-adjusted Life-year (QALY) estimated using the EQ-5D-5L, and cost per death avoided at 1 year.
- Participant preference (via qualitative interviews in the pilot study). This qualitative data will complement quantitative outcome assessment.
- Use of alternative therapies.
- Crossover therapies.

## 4.5. Timing of outcome assessments

The schedule of trial procedures and outcome assessments are given in protocol section 8.3.

## 4.6. Randomisation

Participants will be randomised in a 1:1 ratio to either carvedilol or variceal band ligation.

Randomisation will be performed centrally at the Birmingham Clinical Trials Unit (BCTU) using a minimisation algorithm incorporating the following factors:

- Presence or absence of hepatic decompensation (ascites or encephalopathy)
- Size of the largest varix (Grade II, or Grade III)
- Age of the participant at randomisation (18-50, 51-70, >70)
- Presence or absence of alcohol related liver disease

A 'random element' will be included in the minimisation algorithm, so that each participant has a probability (unspecified here), of being randomised to the opposite intervention that they would have otherwise received. Full details of the algorithm used will be stored in a confidential document at BCTU.

## 4.7. Sample size

The sample size calculation has been based on published data from both a Cochrane meta-analysis of variceal banding versus beta-blockers[6] and data from the first UK randomised trial of carvedilol published in this disease area[7]. The Cochrane meta-analysis reported an overall 1-year variceal bleeding rate of 12% in the variceal banding ligation group. The 1-year bleeding rate was chosen for the primary outcome as Kaplan-Meier curves suggest that the majority of variceal bleeding occurs in the first year after treatment[7]. In order to detect a 33% proportional difference in variceal bleeding rates (i.e. from 12% to 8%, a 4% absolute difference) between groups using a 2-sided test for comparison of proportions with a 1:1 allocation ratio, 90% power and a type I error rate of 5% (i.e. $\alpha=0.05$), requires 2362 participants (1181 per group). Assuming and adjusting for a 10% attrition/loss to follow-up rate (based on the similar patient population studied[7]), which is thus a conservative estimate due to our shorter duration of follow-up), increases the required sample size to 2630 participants in total (1315 per group).

## 4.8. Framework

The objective of the trial is to test the superiority of one intervention to another.

The null hypothesis is that there is no difference in the 1-year variceal bleeding rate between the intervention groups. The alternative hypothesis is that there is a difference between the groups.

## 4.9. Interim analyses and stopping guidance

A separate Data Monitoring Committee (DMC) reporting template will be drafted and agreed by the DMC. Interim analyses of major outcomes and safety data will be conducted and provided in strict confidence to the DMC. The statistical methods stated in this SAP will be followed for the outcomes included in the DMC report, where possible.

The DMC will operate in accordance with a trial specific charter based upon the template created by the Damocles Group. The DMC will meet at the end of the 12 months internal pilot trial to assess the safety data, as part of the planned interim analysis and give advice on continuation to the main trial (see Section 4.10). Since this is an internal pilot trial, and these safety data will be included in the main analysis of the CALIBRE trial, these data will remain confidential, except to members of the DMC and the trial statistician(s) performing the analysis.

During the main trial, the DMC will meet at least annually, or as per a timetable agreed between the DMC prior to trial commencement. Data analyses will be supplied in confidence to the DMC, which will be asked to give advice on whether the accumulated data from the trial, together with the results from other relevant research, justifies the continuing recruitment of further participants. The DMC will advise the chair of the TSC if, in their view, any of the randomised comparisons in the trial have provided both (a) "proof beyond reasonable doubt" that for all, or for some, types of participant one particular treatment is definitely indicated or definitely contraindicated in terms of a net difference in the major outcomes, and (b) evidence that might reasonably be expected to influence the patient management of many clinicians who are already aware of other main results. The TSC can then decide whether to close or modify any part of the trial.

## 4.10. Internal Pilot Progression Rules

At the end of the 12-month pilot phase, the following targets should be met to justify progression to the main trial:

- Minimum of 250 participants recruited across the 20 sites with 2 sites opening per month and an average of 1.6 participants per month per open site.
- 90% of participants complete data collection at their six months follow-up visit.
- Trial Steering Committee and Data Monitoring Committee report no safety concerns which would prohibit continuation to the main trial.

## 4.11. Timing of final analysis

The final analysis for the trial will occur once all participants have completed the 1-year assessment and the corresponding outcome data has been entered onto the trial database and validated as being ready for analysis. This is provided that the trial has not been stopped early for any reason (e.g. TSC advice or funding body request).

## 4.12. Timing of other analyses

Not applicable.

## 4.13. Trial comparisons

All references in this document to 'group' refer to carvedilol or band ligation.

# 5. Statistical Principles

## 5.1. Confidence intervals and p-values

All estimates of differences between groups will be presented with two-sided 95% confidence intervals, unless otherwise stated. P-values will be reported from two-sided tests at the 5% significance level.

## 5.2. Adjustments for multiplicity

No correction for multiple testing will be made.

## 5.3. Analysis populations

All primary analyses (primary and secondary outcomes including safety outcomes) will be by intention-to-treat (ITT). Participants will be analysed in the intervention group to which they were randomised, and all participants shall be included whether or not they received the allocated intervention. The analysis population for the four week safety form will comprise only those patients randomised to carvedilol. This is to avoid any potential bias in the analysis.

## 5.4. Definition of adherence

Adherence to allocated intervention will be monitored via a participant's medical notes. In particular, in the carvedilol arm, participants will be asked about adherence with their trial medication at each follow-up visit and their response documented in the medical notes and subsequently transcribed onto the Follow-Up CRFs. Adherence to variceal band ligation will be documented on the Follow-Up CRFs using information available in the participant's medical notes.

We will define adherence as follows:

*Carvedilol Arm*

A participant randomised to the carvedilol arm is considered adherent if they report taking their dose of trial medication at least 75% of the time until the end of the trial or until they stop/switch treatment due to medical reasons.

*Variceal Band Ligation Arm*

A participant randomised to variceal band ligation is considered adherent if they attend all scheduled banding appointments. Note that a rescheduled appointment is not considered missed and that varices may not be eradicated if a person stops/switches treatment due to medical reasons.

In the case that withdrawal, death or loss to follow-up lead to early exit from the trial, adherence is based on adherence information collected up the point of exit.

### 5.5. Handling protocol deviations and violations

A protocol deviation/violation is defined as a failure to adhere to the protocol such as errors in applying the inclusion/exclusion criteria, the incorrect intervention being given, incorrect data being collected or measured, follow-up visits outside the visit window or missed follow-up visits. We will apply a strict definition of the ITT principle and will include all participants as per the ITT population described in section 5.3 in the analysis in some form regardless of deviation from the protocol.[8] This includes participants who were randomised but later found to violate the inclusion or exclusion criteria. This does not include those participants who have specifically withdrawn consent for the use of their data in the first instance; however these outcomes will be explored as per other missing responses.

### 5.6. Unblinding

This is an open label study and the allocated intervention is recorded on the case report forms. Interim analyses will be produced by trial arm (i.e. Carvedilol vs. variceal band ligation) Unblinding of the Trial Statistician to the allocated intervention code will take place prior to each of the interim analyses (with enough time to produce the report) and after the database is locked for final analysis.

## 6. Trial population

### 6.1. Recruitment

A flow diagram (as recommended by CONSORT[9]) will be produced to describe the participant flow through each stage of the trial. This will include information on the number (with reasons) of losses to follow-up (drop-outs and withdrawals) over the course of the trial. A template for reporting this is given in the final report template.

### 6.2. Baseline characteristics

The trial population will be tabulated as per the table in the final report template. Categorical data will be summarised by number of participants, counts and percentages. Continuous data will be summarised by the number of participants, mean and standard deviation if deemed to be normally distributed or number of participants, median and interquartile range if data appear skewed, and ranges if appropriate. Tests of statistical significance will not be undertaken, nor confidence intervals presented.[10]

## 7. Intervention(s)

### 7.1. Description of the intervention(s)

A template for reporting information on the intervention(s) is given in the final report template. As variceal band ligation is a surgical intervention this includes information such as the number of banding procedures needed to eradicate the varices.

## 7.2. Adherence to allocated intervention

A cross-tabulation of allocated intervention by the adherence categories stated in section 5.4 will be produced (proportions and percentages). A template for reporting adherence is given in the final report template. This includes tables for reporting information such as whether the additional 4-week follow-up visit was attended by those in the carvedilol group.

# 8. Protocol deviations and violations

Frequencies and percentages by group will be tabulated for the protocol deviations and violations as per the final report template.

# 9. Analysis methods

## 9.1. Covariate adjustment

In the first instance, intervention effects between groups for all outcomes will be adjusted for the minimisation parameters listed in section 4.6. Categorised continuous variables (e.g. age) will be treated as continuous variables in this adjustment. Other covariate adjustment will be baseline values for parameters where available (e.g. EQ-5D-5L will be adjusted for baseline score).

If covariate adjustment is not possible (e.g. the model does not converge), unadjusted estimates will be produced, and it will be made clear in the final report why this occurred (e.g. not possible due to low event rate/lack of model convergence).

If the log-binomial model fails to converge, a Poisson regression model with robust standard errors will be used to estimate the same parameters.[11] If this also fails to converge, unadjusted estimates will be produced from the log-binomial model. It will be made clear in the final report why this occurred (e.g. not possible due to low event rate/lack of model convergence).

## 9.2. Distributional assumptions and outlying responses

Distributional assumptions (e.g. normality of regression residuals for continuous outcomes) will be assessed visually prior to analysis; although in the first instance the proposed primary method of estimation in this analysis plan will be followed. If responses are considered to be particularly skewed and/or distributional assumptions violated, the impact of this will be examined through sensitivity analysis; this will consist of transformation of responses prior to analysis (e.g. log transformation) in the first instance. If extreme values are apparent and considered to be affecting the integrity of the analysis, a sensitivity analysis consisting of removing the outlying response(s) and repeating the analysis will be performed. Output from these analyses, if performed, will be described and presented alongside the original analysis (or included, e.g. in appendices) with the excluded values clearly labelled. See section 9.10 for further details.

## 9.3. Handling missing data

In the first instance, analysis will be completed on received data only with every effort made to follow-up participants even after protocol violation to minimise any potential for bias. For the primary outcome, a participant will be assumed to not have experienced variceal bleeding unless there is evidence to suggest otherwise. Therefore, there will be no missing data in this outcome for the primary analysis. However, sensitivity analysis will be performed on the primary outcome measure.[12] See section 9.10 for further details.

## 9.4. Data manipulations

The Trial Statistician will derive all responses from the raw data recorded in the database.

**Primary outcome measure**

The primary outcome measure for the comparison of carvedilol vs. variceal band ligation is variceal bleeding within 1 year of randomisation. We will define 1 year to be 365 days.

Variceal bleeding events are identified on the 6 month and 12 month follow-up forms. If a participant has at least one episode of variceal bleeding (including banding-related bleeding) indicated on either of these follow-up forms then they have "experienced variceal bleeding". Participants which do not fulfil this criteria for experiencing variceal bleeding will be termed "free from variceal bleeding".

For the primary outcome analysis, if a participant has "experienced variceal bleeding" and the date of onset of the first episode of bleeding as indicated on the follow-up form is within 365 days of the date of randomisation then the question "Has variceal bleeding occurred within 1 year of randomisation?" will be answered "yes". Otherwise, it will be answered "no". This will form the primary binary outcome.

**Secondary outcome measures**

*Time to first variceal bleed*

Variceal bleeding events are identified as adverse events on the 6 month and 12 month follow-up forms. If a participant has at least one episode of variceal bleeding (including banding-related bleeding) indicated on either of these follow-up forms then they have "experienced variceal bleeding". Participants which do not fulfil this criteria for experiencing variceal bleeding will be termed "free from variceal bleeding".

If a participant is termed as having "experienced variceal bleeding" then their time until bleeding will be defined as the number of days from the date of randomisation till the date of onset for the first variceal bleed occurring.

Participants who are termed as "free from variceal bleeding" and who were last known to be

bleeding-free prior to the 12-month follow-up time-point will censored in the analysis. The time to censoring will be the number of days from the date of randomisation to the date last known to be free from variceal bleeding. This will be determined by the completion date of the most recent trial form received e.g. date of completion on the 12-month follow-up form. If the last known date in the trial is equal to the date of randomisation then the participant will be censored at day 1.

*All-cause mortality at one year*

Deaths are identified on the SAE form. If a participant has death indicated on an SAE form via the 'death' variable then they will be termed "dead". Participants which do not fulfil this criteria will be termed "alive".

If a participant is "dead" then their time till death will be defined as the number of days from the date of randomisation till the date death. If the time till death is ≤ 365 days then the question "All-cause mortality occurred by one year?" is termed "yes". If the time till death is > 365 days then the question "All-cause mortality occurred by one year?" is termed "no".

If a participant is "alive" then their time till censoring will be the number of days from the date of randomisation to the date last known to be alive. This will be determined by the completion date of the most recent trial form received. If the censored time is > 365 then the question "All-cause mortality occurred by one year?" is answered "no". If the censored time ≤ 365 days but the follow-up CRF has been completed within the window of assessment (no earlier than 351 days from randomisation) then "All-cause mortality occurred by one year?" is answered "no". If the censored time is ≤ 365 and the 12-month follow-up CRF has not been returned within the scheduled window of assessment then there is not complete information regarding mortality within 1 year for this participant. Therefore, the answer to "All-cause mortality occurred by one year?" is considered to be missing.

Liver-related mortality and cardiovascular mortality will be derived in a similar fashion. Deaths of these types will be classified and indicated on the database following review of the SAE information provided by the chief investigator.

*Transplant free survival at one year*

Liver transplants are identified on the SAE form. If a participant has a liver transplant indicated via the CTCAE category on the SAE form then the participant is termed as having "had a liver transplant". Participants which do not fulfil this criteria will be termed as having "not had a liver transplant".

If a participant has "had a liver transplant" then their time till transplant will be defined as the number of days from the date of randomisation till the date of transplant (indicated by the date of onset on the SAE form). If the time till transplant is ≤ 365 days the question "Transplant free survival at 1 year?" is answered "no". If the time till transplant is > 365 the question

"Transplant free survival at 1 year?" is answered "yes".

If a participant is "transplant free" then their time till transplant will be the number of days from the date of randomisation to the date last known to be transplant free. This will be determined by the completion date of the most recent trial form received. If the censored time is > 365 then the question "Transplant free survival at 1 year?" is answered "yes". If the censored time ≤ 365 days but the follow-up CRF has been completed within the window of assessment (no earlier than 351 days from randomisation) then "Transplant free survival at 1 year?" is still answered "yes". If the censored time is ≤ 365 and the 12-month follow-up CRF has not been returned then there is not complete information regarding transplant free survival within 1 year for this participant. Therefore, the answer to "Transplant free survival at 1 year?" is considered to be missing.

*EQ-5D-5L*
EQ-5D-5L is a 6-item questionnaire consisting of five multiple-choice questions and one visual analogue scale. The five multiple-choice questions concern different aspects of health (mobility, self-care, usual activities, pain/discomfort and anxiety/depression) and each require the participant to select one of five ordered responses to indicate their level of problems with each aspect. The permitted responses are chosen from one of the following: no problems=1, slight problems=2, moderate problem=3, severe problems=4, extreme problems/unable to perform activity=5. The numbers assigned to each state are then converted using the official scoring method to obtain a score. The numbers 1-5 assigned to each state have no arithmetic properties and will not be used as cardinal scores themselves. The visual analogue scale is numbered from 0 to 100, where 0 indicates the worst health a participant can imagine and 100 indicates the best health a participant can imagine. Higher scores are better for both the multiple-choice final score and the visual analogue scale. Due to the subjective nature of a questionnaire, EQ-5D responses outside of the scheduled window of assessment (+/-2 months) will be removed in a sensitivity analysis.

**Other Measures**

*Age at randomisation*
Calculated as the number of years from the year of birth to the year of randomisation, with a subtraction of 1 if the birthday has not been yet reached in the randomisation year.

*Body Mass Index (BMI)*
Calculated using the weight and height of a participant obtained from clinical records via the following formula; BMI = Weight (kg)/Height (m)$^2$

*MELD Score*
The MELD score is calculated via a formula involving serum creatinine (mg/dL), serum bilirubin (mg/dL) and INR which are all components of standard care blood test results. Where INR is

not available, it will be calculated using prothrombin time: INR = PTpatient/PTcontrol. The data will be collected on the baseline and follow-up CRFs. Serum creatinine and serum bilirubin are recorded in µmol/L on the CRFs. The following conversion is used to obtain values for these measures in mg/dl:

Serum creatinine (mg/dL) = 0.01131222 x Serum creatinine (µmol/L)
Serum bilirubin (mg/dL) = 0.01131222 x Serum bilirubin (µmol/L)

These values can then be used directly in the MELD score formula as follows:

$$\text{MELD Score} = 10 \times [0.957 \times \ln(\max(1, \min(\text{serum creatinine (mg/dL)}, 4))) + 0.378 \times \ln(\max(1, \text{serum bilirubin (mg/dL)})) + 1.120 \times \ln(\max(1, \text{INR})) + 0.643]$$

Where the ln() function takes the natural logarithm, the max(x, y) function takes the larger of the values x and y and the min(x, y) function takes the smaller of the values x and y. The MELD score should then be rounded to the nearest integer.

*Child-Pugh Score*

The Child-Pugh score is calculated from blood test results, presence of ascites and presence of encephalopathy all of which are recorded on the baseline and follow-up CRFs. The blood test results required are serum albumin (mg/dL), INR and serum bilirubin (mg/dL). Where INR is not available, it will be calculated using prothrombin time: INR = PTpatient/PTcontrol. Serum albumin is recorded in g/L on the CRFs and serum bilirubin is recorded in µmol/L on the CRFs. The following conversions are performed before use in the Child-Pugh score formula:

Serum albumin (mg/dL) = 100 x Serum albumin (µmol/L)
Serum bilirubin (mg/dL) = 0.01131222 x Serum bilirubin (µmol/L)

The five constituent variables are each separately categorised into one of three levels (1, 2, 3) in the following way:

| | Score | | |
|---|---|---|---|
| | 1 | 2 | 3 |
| | | | |
| Serum albumin (mg/dL) | >3.5 | 2.8-3.5 | <2.8 |
| INR | <1.7 | 1.7-2.3 | >2.3 |
| Serum bilirubin (mg/dL) | <2 | 2-3 | >3 |
| Ascites | Absent | Slight | Moderate/Severe |
| Encephalopathy | Absent | Moderate(Stage I-II) | Severe(Stage III-IV) |

The Child-Pugh score is then calculated for each participant by adding the five scored

components:

Child-Pugh Score = Serum albumin score + INR score + Serum bilirubin score
+ Ascites score + Encephalopathy score.

The minimum possible score is 5 and the maximum possible score is 15.
The Child-Pugh score is then converted into the Child-Pugh grade as follows:

| Child-Pugh Score | Child-Pugh Grade |
|---|---|
| 5 to 6 | A |
| 7 to 9 | B |
| 10 to 15 | C |

*Time to eradication of varices (VBL participants only)*
The date that varices are eradicated is captured on the follow-up CRF. The time till eradication is calculated by taking the difference between date of randomisation and date of eradication.

*Alcohol Consumption*
If the answer to "Does the participant drink alcohol?' is "no" then the response to "On average, how many units of alcohol has the participant consumed per week over the last 12 weeks?" will be by default missing. In this case, the missing value is replaced with 0.

*Coffee Consumption*
If the answer to "Does the participant drink coffee?' is "no" then the response to "On average, how many cups of coffee does the participant drink per day?" will be by default missing. In this case, the missing value is replaced with 0.

## 9.5. Analysis methods – primary outcome(s)

The primary outcome measure of the study is variceal bleeding within the first year after randomisation. This outcome is a binary outcome (i.e. yes/no) and all recruited participants will feature in the primary analysis population. The number and percentage of participants experiencing variceal bleeding within 1 year of randomisation will be reported by treatment group. An adjusted risk ratio and 95% confidence interval will be estimated from a log-binomial model. Risk difference will also be reported. See section 9.1 for covariate adjustment and model convergence. A template for reporting the primary outcome is given in the final report template.

## 9.6. Analysis methods – secondary outcomes

The secondary outcomes for the trial include continuous, categorical and time-to-event data

items. The analysis population for these outcomes will be restricted to those participants who have reached and provided data for the time point of interest.

*Time To First Variceal Bleed*
Time to first variceal bleed will be compared between treatment groups using standard survival analysis methods. Kaplan-Meier survival curves will be constructed for visual presentation of time-to-event comparisons. Cox proportional hazard models will be fitted to obtain adjusted treatment effects which will be expressed as hazard ratios with 95% confidence intervals.

*Mortality at one year*
For all-cause mortality, the number and percentage of participants who have died within one year of randomisation will be reported by treatment group. An adjusted risk ratio and 95% confidence interval will be estimated from a log-binomial regression model. Risk difference will also be reported. Liver-related mortality and cardiovascular mortality will be analysed in the same way.

*Transplant free survival at one year*
This will be analysed and reported in the same way as all-cause mortality.

*Adverse events relating to treatment*
These events (as listed in section 4.4) will be analysed and reported in the same way as all-cause mortality.

*Other complications of cirrhosis*
These events (as listed in section 4.4) will be analysed and reported in the same way as all-cause mortality.

*EQ-5D-5L*
Both the EQ-5D-5L score and the health status score will be reported using means and standard deviations. The scores will be compared separately between treatment groups with adjusted mean differences and 95% confidence intervals estimated using linear regression models. Changes in both scores from baseline will also be separately modelled.

A template for reporting the secondary outcomes is given in the final report template.

## 9.7. Analysis methods – exploratory outcomes and analyses

Any data that do not form a pre-specified outcome will be presented using simple summary statistics by intervention group (i.e. numbers and percentages for binary data and means (or medians) and standard deviations (or inter-quartile ranges) for continuous normal (or non-normal) data).

Outcomes related to treatment will be presented for the subset of participants that crossed over during the trial. The results will be compared alongside outcomes for participants that did not cross over.

## 9.8. Safety data

The number and percentage of participants experiencing any adverse events, serious adverse events (SAEs) and suspected unexpected serious adverse reactions (SUSARs) will be presented by intervention group. A table for listing further details on the SAEs is provided in the final template report. Where applicable, this includes details on the dosage participants were on when the SAE occurred for those participants receiving carvedilol.

## 9.9. Planned subgroup analyses

Interpretation of subgroup analysis will be treated with caution (output will be treated as exploratory rather than definitive[13]). Analysis will be limited to the primary outcome only and the subgroups will be formed around the same variables used in the minimisation algorithm. Tests for statistical heterogeneity will be performed prior to any examination of effect estimates within subgroups. The effects of these subgroups will be examined by including a treatment group by subgroups interaction parameter in the final regression model. P-values for the interactions will be presented for these and interpreted with appropriate caution due to the reduced power. Analysis of primary outcome will be performed and reported within each subgroup in the same fashion as the primary analysis as described in section 9.5. A template for reporting this analyses is given in the final report template.

## 9.10. Sensitivity analyses

Sensitivity analyses will be limited to the primary outcome and will consist of:

- An analysis is performed the same way as the primary analysis described in section 9.5 but now limited to the population that adhered to the allocated intervention as described in section 5.4.
- An analysis is performed to assess the effect of missing responses, in particular, the risk of bias they present. In the primary analysis described in section 9.5, all those recruited who have not reported variceal bleeding within 12 months are assumed to not have experienced the event. Here it is assumed that those who did not report variceal bleeding but have withdrawn, are lost to follow-up or have died within the 12-month follow-up period are missing the primary outcome response. Note that participants who are still actively in the trial at 12-months (are not withdrawn or lost to follow-up) but do not return the 12-month follow-up form will not be assumed missing.

  Making these assumptions, firstly, an analysis is performed restricted to the population which has non-missing responses. Secondly, it is assumed that those with a missing response did not have variceal bleeding - the rational being that the trial team would be

aware if bleeding had occurred due to the seriousness of the event and subsequent completion of an SAE form. Thirdly, the missing responses are assumed to be missing at random and are imputed via multiple imputation. In these three scenarios, the subsequent analysis performed is conducted in the same fashion as the primary analysis described in section 9.5.

- An analysis is performed on the primary analysis population in the same way as described in section 9.5 but with no covariate adjustment.

## 10. Analysis of sub-randomisations

Not applicable.

## 11. Health economic analysis

As indicated in the protocol (section 3.5) there will also be an economic analysis. The details of this analysis are documented separately.

## 12. Statistical software

Statistical analysis will be undertaken in the following statistical software packages: SAS Version 9.4 and/or Stata Version 15.

## 13. References


1. Tripathi D, Stanley AJ, Hayes PC, et al. UK guidelines on the management of variceal haemorrhage in cirrhotic patients. Gut 2015;64:1680-704.
2. NICE. Cirrhosis in over 16s: Assessment and management. London: NICE; 2016.
3. Moore KP, Wong F, Gines P, et al. The management of ascites in cirrhosis: report on the consensus conference of the International Ascites Club. Hepatology 2003;38:258-66.
4. Vilstrup H, Amodio P, Bajaj J, et al. Hepatic encephalopathy in chronic liver disease: 2014 Practice Guideline by the American Association for the Study of Liver Diseases and the European Association for the Study of the Liver. Hepatology 2014;60:715-35.
5. Angeli P, Gines P, Wong F, et al. Diagnosis and management of acute kidney injury in patients with cirrhosis: revised consensus recommendations of the International Club of Ascites. Gut 2015;64:531-7.
6. Gluud LL, Krag A. Banding ligation versus beta-blockers for primary prevention in oesophageal varices in adults. CochraneDatabaseSystRev 2012;8:CD004544.
7. Tripathi D, Ferguson JW, Kochar N, et al. Randomized controlled trial of carvedilol versus variceal band ligation for the prevention of the first variceal bleed. Hepatology

2009;50:825-33.
8. Gupta SK. Intention-to-treat concept: A review. Perspect Clin Res. 2011;2(3):109-112.
9. Schulz KF, Altman DG, Moher D, for the CONSORT Group. CONSORT 2010 Statement: updated guidelines for reporting parallel group randomised trials. BMJ. 2010;340:c332.
10. Altman DG, Dore CJ. Randomisation and baseline comparisons in clinical trials. Lancet. 1990;335:149–53.
11. A modified Poisson regression approach to prospective studies with binary data. Am J Epidemiol. 2004;159(7):702-6.
12. White IR, Horton NJ, Carpenter J, Pocock SJ. Strategy for intention to treat analysis in randomised trials with missing outcome data. BMJ. 2011;342:d40.
13. Wand R, Lagakos SW, Ware JH, Hunter DJ, Drazen JM. Reporting of subgroups analyses in clinical trials. NEJM. 2007;357:2189-94.

## Appendix A: Deviations from SAP

This report below follows the statistical analysis plan dated <insert effective date of latest SAP> apart from following:

| Section of report not following SAP | Reason |
|---|---|
| <insert section > | <insert, e.g. exploratory analyses request by TMG> |

# Appendix B: Trial schema

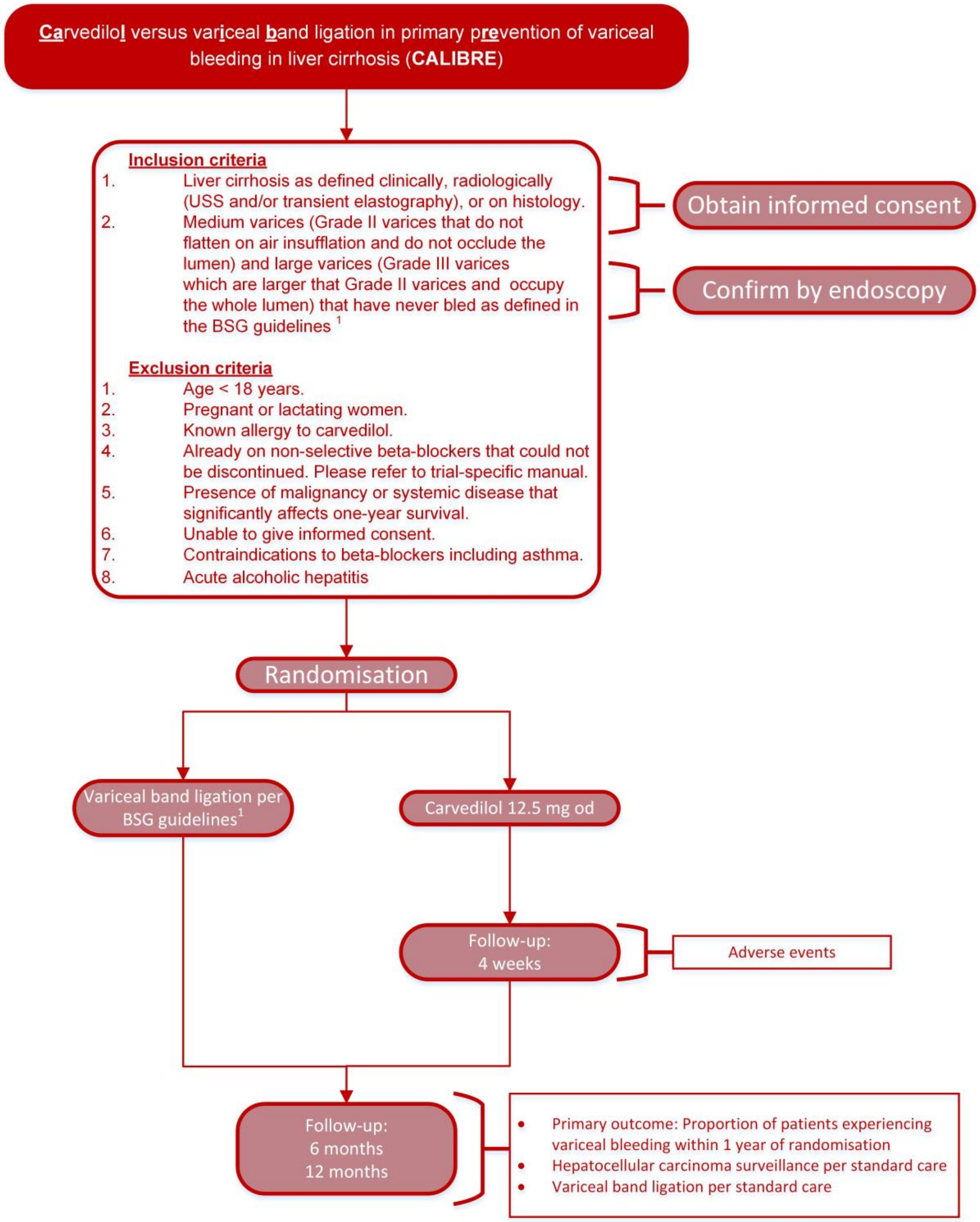


[1]Tripathi D, Stanley AJ, Hayes PC, Patch D, Millson C, Mehrzad H, Austin A, Ferguson JW, Olliff SP, Hudson M, Christie JM; Clinical Services and Standards Committee of the British Society of Gastroenterology. U.K. guidelines on the management of variceal haemorrhage in cirrhotic patients. Gut. 2015 Nov;64(11):1680-704.

## Appendix C: Schedule of assessments

**Table of Assessments**

| | Randomisation and Baseline | 4 weeks[¥] ± 1 week FU visit | 6 months ± 2 months FU visit | 12 months ± 2 months FU visit |
|---|---|---|---|---|
| Confirm eligibility | ✓ | | | |
| Seek informed consent | ✓ | | | |
| Randomisation | ✓ | | | |
| Medical history[#] | ✓ | | | |
| Medication review | ✓ | ✓ | ✓ | ✓ |
| Physical examination | ✓* | | ✓* | ✓* |
| Office blood pressure | ✓ | ✓ | ✓ | ✓ |
| Pulse | ✓ | ✓ | ✓ | ✓ |
| Standard care blood tests | ✓* | | ✓* | ✓* |
| Height | ✓* | | | |
| Weight | ✓* | ✓* | ✓* | ✓* |
| Administer EQ 5D-5L | ✓ | | ✓ | ✓ |
| Resource use (Follow-Up CRFs) | | | ✓ | ✓ |
| Dispense trial medication[¥ ∞] | ✓ | | | |
| Adverse event review and evaluation | | ✓ | ✓ | ✓ |
| Adherence | | | ✓ | ✓ |
| Qualitative interviews[§] | | ✓ | | ✓ |

#Including aetiology of liver disease and past medical history (diabetes, ischaemic heart disease, pulmonary disease).
*Taken from clinical records.
[¥]Carvedilol arm only.
∞Medication may initially be dispensed by site but can subsequently be dispensed by the participant's community pharmacy.
[§]Pilot phase only

## Appendix D: Template report

A template report for the final analyses will be provided in a separate document.
