# Supplementary material for: Clinical Trial: A Multicentre Randomised Controlled Trial of Carvedilol Versus Variceal Band Ligation in Primary Prevention of Variceal Bleeding in Liver Cirrhosis (CALIBRE Trial)
Source: Aliment Pharmacol Ther. 2025 Apr 16;61(11):1740–54. doi: 10.1111/apt.70080 (PMC12074564; doi:10.1111/apt.70080)
Supplement: Supplementary file 1 — Data S1: [file APT-61-1740-s001.zip › apt70080-sup-0003-CALIBRE _Appendix_April2025_R2_Clean.docx]

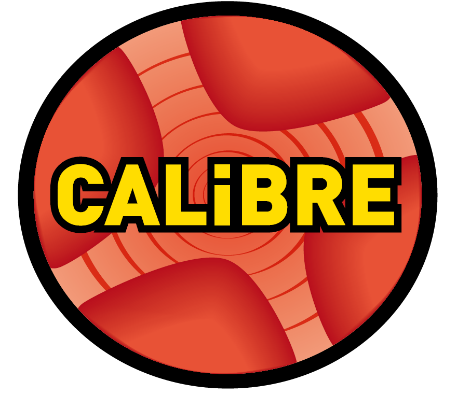

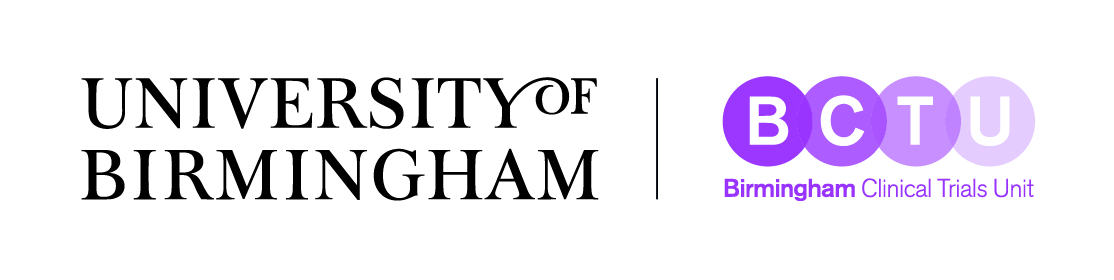


**The CALIBRE Trial:**

**Carvedilol versus variceal band ligation in primary prevention of variceal bleeding in liver cirrhosis**

**Appendix 1**

Contents

[1. CONSORT 5](#_Toc194962861)

[2. Data Completeness 7](#_Toc194962862)

[2.1. Data Completeness for the Primary Outcome 7](#_Toc194962863)

[3. Protocol Deviations 8](#_Toc194962864)

[4. Participant characteristics at randomisation 9](#_Toc194962865)

[5. Adherence to treatment allocation 14](#_Toc194962866)

[6. Alcohol consumption during follow up 15](#_Toc194962867)

[1.1 Data for those who answered “no” to minimisation variable for alcohol-related liver disease 15](#_Toc194962868)

[1.2 Data for those who answered “yes” to minimisation variable for 17](#_Toc194962869)

[alcohol-related liver disease 17](#_Toc194962870)

[7. Analysis of secondary outcome measures 19](#_Toc194962871)

[7.1. Mortality within 1 Year of Randomisation 21](#_Toc194962872)

[7.2. Transplant-Free Survival 22](#_Toc194962873)

[7.3. Other Complications of Cirrhosis (within 1 year of randomisation) 23](#_Toc194962874)

[New Onset Ascites 24](#_Toc194962875)

[Spontaneous Bacterial Peritonitis 25](#_Toc194962876)

[Hepatocellular Carcinoma 26](#_Toc194962877)

[Hepatic Encephalopathy 27](#_Toc194962878)

[8. Analysis of exploratory outcome measures 28](#_Toc194962879)

[8.1. Hepatic Decompensation 28](#_Toc194962880)

[Hepatic Decompensation – definition 1 29](#_Toc194962881)

[Hepatic Decompensation – definition 2 31](#_Toc194962882)

[9. Safety – Serious Adverse Events 33](#_Toc194962883)

[10. Economic evaluation 34](#_Toc194962884)

[Methods 34](#_Toc194962885)

[Results 36](#_Toc194962886)

[**Figure S1:** CONSORT flow diagram 5](#_Toc186702893)

[**Figure S2**: Kaplan Meier: overall mortality 21](#_Toc186702894)

[**Figure S3**: Kaplan Meier: transplant free survival 22](#_Toc186702895)

[**Figure S4**: Kaplan Meier: New Onset Ascites 24](#_Toc186702896)

[**Figure S5**: Kaplan Meier: Spontaneous Bacterial Peritonitis 25](#_Toc186702897)

[**Figure S6**: Kaplan Meier: Hepatocellular Carcinoma 26](#_Toc186702898)

[**Figure S7**: Kaplan Meier: Hepatic Encephalopathy 27](#_Toc186702899)

[**Figure S8**: Cost-Effectiveness plane for Carvedilol versus VBL – Complete case analysis 48](#_Toc186702900)

[**Figure S9**: Cost-effectiveness acceptability curve. Indicates the probability of carvedilol being cost-effective across different willingness-to-pay thresholds per additional QALY 49](#_Toc186702901)

[Table S1: Reasons for ineligibility/declining participation 6](#_Toc186702902)

[Table S2: Form return rate by group 7](#_Toc186702903)

[Table S3: Data completeness for the primary outcome 7](#_Toc186702904)

[Table S4: Protocol deviations by treatment group 8](#_Toc186702905)

[**Table S5:** Baseline characteristics split by treatment group and overall 9](#_Toc186702906)

[**Table S6:** Baseline concomitant medications split by treatment group and overall 12](#_Toc186702907)

[**Table S7**: Treatment adherence by group 14](#_Toc186702908)

[**Table S8**: Baseline alcohol consumption – “no” to minimisation variable for alcohol-related liver disease 15](#_Toc186702909)

[**Table S9**: Six-month alcohol consumption – “no” to minimisation variable for alcohol-related liver disease 15](#_Toc186702910)

[**Table S10**: 12-month alcohol consumption – “no” to minimisation variable for alcohol-related liver disease 16](#_Toc186702911)

[**Table S11**: Baseline alcohol consumption – “yes” to minimisation variable for alcohol-related liver disease 17](#_Toc186702912)

[**Table S12**: Six month alcohol consumption – “yes” to minimisation variable for alcohol-related liver disease 17](#_Toc186702913)

[**Table S13**: 12-month alcohol consumption – “yes” to minimisation variable for alcohol-related liver disease 18](#_Toc186702914)

[**Table S14**: Analysis of hepatic decompensation within 1 year of randomisation – definition 1 (entire population) 29](#_Toc186702915)

[**Table S15**: Analysis of hepatic decompensation within 1 year of randomisation – definition 1 (compensated disease at randomisation) 30](#_Toc186702916)

[**Table S16**: Analysis of hepatic decompensation within 1 year of randomisation – definition 2 (entire population) 31](#_Toc186702917)

[**Table S17**: Analysis of hepatic decompensation within 1 year of randomisation – definition 2 (compensated disease at randomisation) 32](#_Toc186702918)

[**Table S18:** Details of SAEs by group 33](#_Toc186702919)

[**Table S20:** Resource Use Prices and description (price year 2023) 37](#_Toc186702920)

[**Table S21:** Mean healthcare resource use per patient over 12 months - complete follow up data 39](#_Toc186702921)

[**Table S22:** Hospital Services Consumption over 12 month period - Number of participants from complete resource use data 40](#_Toc186702922)

[**Table S23:** Mean aggregated cost over 12 months period for all cost categories (2023 prices- GBP) – complete resource use data 41](#_Toc186702923)

[**Table S24:** Number and percentage of participants with complete and missing EQ-5D-5L Data 43](#_Toc186702924)

[**Table S25:** EQ-5D-5L index score and calculated Quality Adjusted Life Years - 12 months post randomisation (complete case data). 44](#_Toc186702925)

[**Table S26:** EQ-5D-5L VAS scores per participant over 12 months 45](#_Toc186702926)

[**Table S27:** Results of the economic evaluation analyses - base case 46](#_Toc186702927)

[**Table S28:** Results of the scenario analyses 47](#_Toc186702928)

# CONSORT

**Figure S1:** CONSORT flow diagram

**5405 screened/assessed for eligibility**

**Excluded (n=5140)**

- Ineligible for trial (n=4182)
- Patient choice (n=348)
- Other reasons (n=610)

**See Table S1 for details**

**265 randomised**

Allocation

**Allocated to VBL (n=132)**

**Allocated to carvedilol (n=133)**

**Withdrawn (n=2)**

**Died (n=0)**

**Withdrawn (n=0)**

**Died (n=0)**

Safety Visit

4-week Follow-Up

4-week Follow-Up

Safety visit data not collected in VBL arm

**Reached safety visit time-point (n=133)**

**Provided safety visit data (n=127)**

**Missing safety visit data (n=6)**

**Withdrawn (n=3)**

**Died (n=3)**

**Withdrawn (n=3)**

**Died (n=1)**

6-month Follow-Up

**Reached 6m visit time-point (n=124)**

**Provided 6m follow-up data (n=117)**

**Missing 6m follow-up data (n=7)**

**Reached 6m visit time-point (n=129)**

**Provided 6m follow-up data (n=118)**

**Missing 6m follow-up data (n=11)**

**Withdrawn (n=2)**

**Died (n=6)**

**Withdrawn (n=1)**

**Died (n=10)**

12-month Follow-Up

**Reached 12m visit time-point (n=121)**

**Provided 12m follow-up data (n=114)**

**Missing 12m follow-up data (n=7)**

**=====**

**Complete primary outcome data available (n=113)**

**Reached 12m visit time-point (n=113)**

**Provided 12m follow-up data (n=105)**

**Missing 12m follow-up data (n=8)**

**=====**

**Complete primary outcome data available (n=105)**

Table S1: Reasons for ineligibility/declining participation

|  | **Details** | **n** |
| --- | --- | --- |
| **Exclusion criteria = 4,182** | | |
| Liver cirrhosis | No liver disease | 188 |
|  | Awaiting confirmation of cirrhosis | 31 |
| Medium/large varices that have never bled | No varices | 1320 |
|  | Small varices | 695 |
|  | Previous bleed | 218 |
|  | Other varices ineligibility | 3 |
|  | Grade II varices not occluding lumen | 2 |
| Age <18 | Age <18 | 2 |
| Pregnant/lactating | Pregnant/lactating | 0 |
| Contraindications / intolerance to beta-blockers | Asthma | 154 |
|  | Intolerance to beta-blockers | 54 |
|  | Contra-indications to beta-blockers | 10 |
| Contraindications / intolerance to banding | Intolerance to banding | 10 |
|  | Contra-indications to banding | 0 |
| Current or past history of beta-blocker use | Current beta-blockers use | 583 |
|  | Past beta-blockers use | 39 |
| Current or past history of banding | Past VBL | 577 |
|  | Current / planned VBL | 30 |
| Presence of malignancy or systemic disease affecting 1-year survival | Cancer diagnosis | 62 |
|  | Presence of disease affecting 1-year survival | 19 |
| Unable to give informed consent | Unable to give informed consent | 10 |
| Alcoholic hepatitis | Alcoholic hepatitis | 30 |
| TIPSS / shunts | TIPSS | 12 |
| Organ transplant | Post-transplant | 26 |
|  | Awaiting transplant | 3 |
| Co-enrolment | Enrolled in another trial | 6 |
| Ineligible no reason provided | - | 28 |
| Ill at present | - | 32 |
| Other illness not specified | - | 31 |
| Red spots | - | 7 |
| **Declined to participate = 348** | | |
| Patient declined | - | 347 |
| Patient withdrew all consent for trial including for data already collected |  | 1 |
| **Other = 610** | | |
| Endoscopy issues |  | 153 |
| Awaiting OGD date |  | 113 |
| Unable to access records |  | 105 |
| Patient did not attend |  | 102 |
| Patient missed by site staff |  | 21 |
| Research team not available |  | 21 |
| Unable to contact patient |  | 20 |
| Patient died |  | 8 |
| COVID19 |  | 3 |
| Other |  | 64 |

# Data Completeness

Table S2: Form return rate by group

|  | **Carvedilol**  **(n=133)** | | | **VBL**  **(n=132)** | | |
| --- | --- | --- | --- | --- | --- | --- |
| **CRF name** | **Expected** | **Not returned** | **Returned** | **Expected** | **Not returned** | **Returned** |
| Randomisation Notepad | 133 | 0 | 133 (100%) | 132 | 0 | 132 (100%) |
| Baseline | 133 | 0 | 133 (100%) | 132 | 1 | 131 (99%) |
| Safety CRF | 133 | 5 | 127  (95%) | - | - | - |
| 6 month CRF | 129 | 11 | 118  (91%) | 124 | 7 | 117  (94%) |
| 12 month CRF | 121 | 6 | 114  (94%) | 113 | 8 | 105  (93%) |

## Data Completeness for the Primary Outcome

Table 5 shows the amount of data available for the variceal bleeding primary outcome. As per the assumptions noted in the SAP, all participants will be included in the primary outcome analysis.

Table S3: Data completeness for the primary outcome

| **Variceal bleeding within 1 year of randomisation** | **length of follow-up available (days)** | **Number in category**  **(n=265)** |
| --- | --- | --- |
| ✓ | ✓ | 15 (5.7%) |
| X | complete 365 | 149 (56.2%) |
| X | 305-364 | 54 (20.4%) |
| X | 180-304 | 18 (6.8%) |
| X | 150-179 | 4 (1.5%) |
| X | 120-149 | 1 (0.4%) |
| X | 0 | 24 (9.0%) |

# Protocol Deviations

Table S4: Protocol deviations by treatment group

|  | **Carvedilol** | **VBL** |
| --- | --- | --- |
| **Consent** |  |  |
| Clinician not delegated to take consent | 1 |  |
| Clinician signed ICF days before patient | 2 |  |
| Clinician taking consent not submitted CV, GCP, training log to BCTU | 1 |  |
| Incorrect PIS version given to patient | 1 | 2 |
| Incorrect version of ICF used | 1 | 2 |
| Patient ticked boxes instead of initialling them | 3 |  |
| Nurse not delegated to take consent |  | 1 |
| **Randomisation** |  |  |
| Clinician not delegated to randomise | 1 | 2 |
| Nurse not delegated to complete randomisation CRF | 1 |  |
| Randomisation CRF not completed at time of randomisation | 6 | 6 |
| Randomised but subsequently found to be ineligible | 6 | 4 |
| Patient randomised twice |  | 1 |
| **Carvedilol** |  |  |
| Started on split dose instead of full dose | 1 |  |
| **Vital signs** |  |  |
| No vital signs completed | 4 |  |
| **CRFs** |  |  |
| Clinician not delegated to complete CRFs | 1 |  |
| **Visit not carried out** |  |  |
| Safety visit | 2 |  |
| 6 month FU visit | 6 | 6 |
| 12 month FU visit | 2 | 2 |
| **Visit Outside window** |  |  |
| Safety visit | 6 |  |
| 6 month FU visit | 16 | 19 |
| 12 month FU visit | 10 | 12 |
| **EQ-5D not completed** |  |  |
| Baseline | 5 | 5 |
| 6 month | 20 | 22 |
| 12 month | 11 | 12 |
| **SAE** |  |  |
| Nurse not delegated to report SAEs | 3 | 2 |
| SAE not reported within 24 hours | 4 | 6 |
| Clinician not delegated to assess causality |  | 2 |

Additionally, 5 more deviations were reported that were not linked to a particular patient.

- Nurse not delegated to complete DCFs (n=3)
- Nurse incorrectly delegated permission to confirm eligibility (n=1)
- General notification that visits would be outside window during Covid-19 (n=1)

# Participant characteristics at randomisation

**Table S5:** Baseline characteristics split by treatment group and overall

|  |  | **Carvedilol**  **(n=133)** | **VBL**  **(n=132)** | **Overall**  **(n=265)** |
| --- | --- | --- | --- | --- |
| **Ethnicity** |  |  |  |  |
| (note: where n<randomised then data are missing) |  | **(n=133)** | **(n=131)** | **(n=264)** |
| Ethnic Group, n (%) | White – British | 109 (81.9%) | 116 (88.6%) | 225 (85.2%) |
|  | White – Irish | 1 (0.8%) | 2 (1.5%) | 3 (1.1%) |
|  | White – Other | 6 (4.5%) | 2 (1.5%) | 8 (3.0%) |
|  | Asian – Indian | 5 (3.7%) | 3 (2.3%) | 8 (3.0%) |
|  | Asian - Pakistani | 2 (1.5%) | 2 (1.5%) | 4 (1.5%) |
|  | Asian – Chinese | 1 (0.8%) | 0 | 1 (0.4%) |
|  | Asian – Other | 1 (0.8%) | 0 | 1 (0.4%) |
|  | Black – African | 0 | 2 (1.5%) | 2 (0.8%) |
|  | Arab | 0 | 1 (0.8%) | 1 (0.4%) |
|  | Mixed - White/Black | 1 (0.8%) | 0 | 1 (0.4%) |
|  | Other | 2 (1.5%) | 0 | 2 (0.8%) |
|  | Not provided | 5 (3.7%) | 3 (2.3%) | 8 (3.0%) |
| **Medical history (more than one can be selected)** |  |  |  |  |
| (note: where n<randomised then data are missing) |  | **(n=133)** | **(n=131)** | **(n=264)** |
| Hypertension, n (%) |  | 51 (38.3%) | 47 (35.9%) | 98 (37.1%) |
| Peripheral vascular disease, n (%) |  | 2 (1.5%) | 0 | 2 (0.8%) |
| Transient ischaemic attack, n (%) |  | 4 (3.0%) | 7 (5.3%) | 11 (4.2%) |
| Stroke, n (%) |  | 0 | 1 (0.8%) | 1 (0.4%) |
| Type 1 diabetes, n (%) |  | 2 (1.5%) | 1 (0.8%) | 3 (1.1%) |
| Type 2 diabetes, n (%) |  | 50 (37.6%) | 48 (36.6%) | 98 (37.1%) |
| Hypercholesterolaemia, n (%) |  | 26 (19.5%) | 15 (11.5%) | 41 (15.5%) |
| Myocardial infarction, n (%) |  | 3 (2.3%) | 3 (2.3%) | 6 (2.3%) |

Continued over page ->

|  |  | **Carvedilol**  **(n=133)** | **VBL**  **(n=132)** | **Overall**  **(n=265)** |
| --- | --- | --- | --- | --- |
| **Medical history (continued)** | | | | |
| (note: where n<randomised then data are missing) |  | **(n=133)** | **(n=131)** | **(n=264)** |
| Ascites, n (%) | Yes | 32 (24.1%) | 33 (25.2%) | 65 (24.6%) |
| *Grade:* | *Grade 1* | 15 | 12 | 27 |
|  | *Grade 2* | 8 | 13 | 21 |
|  | *Grade 3* | 7 | 7 | 14 |
|  | *Grade missing* | 2 | 1 | 3 |
| Spontaneous bacterial peritonitis, n (%) |  | 3 (2.3%) | 2 (1.5%) | 5 (1.9%) |
| Hepatorenal syndrome, n (%) |  | 2 (1.5%) | 1 (0.8%) | 3 (1.1%) |
| Hepatocellular carcinoma, n (%) |  | 1 (0.8%) | 1 (0.8%) | 2 (0.8%) |
| *BCLC stage:* | *Stage 0* | 1 |  | 1 |
|  | *Stage A* |  |  |  |
|  | *Stage B* |  | 1 | 1 |
| Hepatic encephalopathy, n (%) |  | 12 (9.0%) | 2 (1.5%) | 14 (5.3%) |
| *Grade:* | *Grade 1* | 7 | 1 | 8 |
|  | *Grade 2* | 1 |  | 1 |
|  | *Grade 3* | 1 |  | 1 |
|  | *Grade missing* | 3 | 1 | 4 |
| **Alcohol** | | | | |
| (note: where n<randomised then data are missing) |  | **(n=133)** | **(n=131)** | **(n=264)** |
| Alcohol consumed in the past 12 months, n (%) | Yes | 57 (42.9%) | 66 (50.4%) | 123 (46.6%) |
| If alcohol consumed, average units per week | N | 53 | 64 | 117 |
| (note: where N<”yes” then data are missing) | Median [IQR] | 10 [3, 40] | 20 [4, 65] | 12 [3, 50] |
|  | Range | 1, 200 | 1, 280 | 1, 280 |
| If alcohol consumed, AUDIT-C undertaken, n (%) | Yes | 39 / 57 (68.4%) | 50 / 66 (75.8%) | 89 / 123 (72.4%) |
| AUDIT-C score | N | 39 | 50 | 89 |
|  | Median [IQR] | 4 [2, 11] | 5.5 [3, 11] | 5 [3, 11] |
|  | Range | 0, 12 | 0, 12 | 0, 12 |
| Where AUDIT-C undertaken, score ≥ 5, n (%) | Yes | 19 / 39 (48.7%) | 29 / 50 (58.0%) | 48 / 89 (53.9%) |
| Total AUDIT score | N | 19 | 29 | 48 |
|  | Median [IQR] | 20 [12, 25] | 15 [10, 21] | 17 [10.5, 22] |
|  | Range | 0, 36 | 0, 32 | 0, 36 |
| **Smoking** |  |  |  |  |
| (note: where n<randomised then data are missing) |  | **(n=133)** | **(n=131)** | **(n=264)** |
| Smoking status, n (%) | Never smoked | 53 (39.8%) | 55 (42.0%) | 108 (40.9%) |
|  | Ex-smoker | 48 (36.1%) | 52 (39.7%) | 100 (37.9%) |
|  | Current smoker | 32 (24.1%) | 24 (18.3%) | 56 (21.2%) |
| If smoked in past 12 months, how many cigarettes smoked daily | N | 35 | 22 | 57 |
|  | Median [IQR] | 10 [7, 20] | 10 [4, 20] | 10 [6, 20] |
|  | Range | 4, 50 | 3, 28 | 3, 50 |

Continued over page ->

|  |  | **Carvedilol**  **(n=133)** | **VBL**  **(n=132)** | **Overall**  **(n=265)** |
| --- | --- | --- | --- | --- |
| **Coffee** |  |  |  |  |
|  |  | **(n=127)** | **(n=125)** | **(n=252)** |
| Coffee consumption daily, n (%) |  | 73 (57.5%) | 61 (48.8%) | 134 (53.2%) |
| If coffee consumed daily, cups per day | N | 72 | 60 | 132 |
|  | Median [IQR] | 2 [1, 4] | 2 [1, 3] | 2 [1, 3] |
|  | Range | 1, 20 | 1, 8 | 1, 20 |
| **Ultrasound** |  |  |  |  |
|  |  | **(n=133)** | **(n=131)** | **(n=264)** |
| Abdominal ultrasound done, n (%) |  | 116 (87.2%) | 115 (87.8%) | 231 (87.5%) |
| Hepatomegaly, n (%) |  | 16 / 100 (16%) | 18 / 88 (20.5%) | 34 / 188 (18.1%) |
| Splenomegaly, n (%) |  | 69 / 116 (59.5%) | 66 / 115 (57.4%) | 135 / 231 (58.4%) |
| *Size (cm):* | *N* | *69* | *64* | *133* |
|  | *Median [IQR]* | *15 [14, 17]* | *15 [14, 17]* | *15 [14, 17]* |
|  | *Range* | *12, 23* | *9, 22* | *9, 23* |
| Hepatic vein patent, n (%) |  | 47 / 50 (94.0%) | 44 / 44 (100%) | 91 / 94 (96.8%) |
| Portal vein patent, n (%) |  | 100 /103 (97.1%) | 98 / 100 (98.0%) | 198 / 203 (97.5%) |
| **Diagnostic endoscopy** |  |  |  |  |
|  |  | **(n=133)** | **(n=131)** | **(n=264)** |
| Oesophageal varices present, n | Grade I | 40 | 29 | 69 |
| (Note: can select more than one grade) | Grade II | 124 | 124 | 248 |
|  | Grade III | 12 | 10 | 22 |
| Oesophageal red signs present, n (%) |  | 14 / 133 (10.5%) | 15 / 130 (11.5%) | 29 / 263 (11.0%) |
| Gastric varices present, n (%) |  | 8 / 133 (6.0%) | 11 / 131 (8.4%) | 19 / 264 (7.2%) |
| GOV1, n | Small | 1 | 2 | 3 |
|  | Medium | 3 | 3 | 6 |
|  | Not present | 3 | 6 | 9 |
| GOV2, n | Small |  |  |  |
|  | Medium |  | 4 | 4 |
|  | Not present | 7 | 7 | 14 |
| IGV1, n | Small | 2 | 1 | 3 |
|  | Medium |  | 1 | 1 |
|  | Large | 1 |  | 1 |
|  | Not present | 4 | 9 | 13 |
| IGV2, n | Small |  |  |  |
|  | Medium |  | 2 | 2 |
|  | Large |  | 1 | 1 |
|  | Not present | 7 | 8 | 15 |
| Gastric red signs present, n |  | 1 |  | 1 |
| **ECG** |  |  |  |  |
|  |  | **(n=133)** | **(n=131)** | **(n=264)** |
| ECG performed, n (%) |  | 11 (8.3%) | 14 (10.7%) | 25 (9.5%) |
| ECG results, n (%) | Within normal limits | 9 | 7 | 16 |
|  | Abnormal, not significant | 2 | 5 | 7 |
|  | Abnormal, significant |  | 2 | 2 |
| If significant, abnormality type | Rhythm |  | 2 | 2 |
|  | Conduction |  |  |  |
|  |  |  |  |  |

**Table S6:** Baseline concomitant medications split by treatment group and overall

|  |  | **Carvedilol**  **(n=133)** | **VBL**  **(n=132)** | **Overall**  **(n=265)** |
| --- | --- | --- | --- | --- |
| **Concomitant medications** | | | | |
| Anticoagulants, n (%) |  | 4 / 133 (3.0%) | 2 / 131 (1.5%) | 6 / 264 (2.3%) |
| Rivaroxaban |  | 2 | 2 | 4 |
| Warfarin |  | 1 |  | 1 |
| Enoxaparin |  | 1 |  | 1 |
| Antiplatelets, n (%) |  | 14 / 133 (10.5%) | 12 / 131 (9.2%) | 26 / 264 (9.8%) |
| Aspirin |  | 9 | 7 | 16 |
| Clopidogrel |  | 5 | 4 | 9 |
| Multiple agents |  |  | 1 | 1 |
| ACE inhibitors, n (%) |  | 28 / 133 (21.1%) | 25 / 131 (19.1%) | 53 / 264 (20.1%) |
| Ramipril |  | 17 | 15 | 32 |
| Lisinopril |  | 7 | 6 | 13 |
| Perindopril |  | 4 | 4 | 8 |
| Angiotensin II receptor blockers, n (%) |  | 8 / 133 (6.0%) | 6 / 131 (4.6%) | 14 / 264 (5.3%) |
| Losartan |  | 3 | 2 | 5 |
| Candesartan |  | 4 | 4 | 8 |
| Valsartan |  | 1 |  | 1 |
| Calcium channel blockers, n (%) |  | 14 / 133 (10.5%) | 12 / 131 (9.2%) | 26 / 264 (9.8%) |
| Amlodipine |  | 10 | 11 | 21 |
| Lacidipine |  | 2 |  | 2 |
| Felodipine |  | 2 |  | 2 |
| Diltiazem |  |  | 1 | 1 |
| Statins, n (%) |  | 41 / 133 (30.8%) | 30 / 131 (22.9%) | 71 / 264 (26.9%) |
| Simvastatin |  | 16 | 11 | 27 |
| Atorvastatin |  | 21 | 15 | 36 |
| Pravastatin |  | 1 | 4 | 5 |
| Rosuvastatin |  | 3 |  | 3 |
| Alpha blockers, n (%) |  | 6 / 133 (4.5%) | 5 / 131 (3.8%) | 11 / 264 (4.2%) |
| Tamsulosin |  | 5 | 5 | 10 |
| Doxazosin |  | 1 |  | 1 |
| Beta blockers, n (%) |  | 9 / 133 (6.8%) | 8 / 131 (6.1%) | 17 / 264 (6.4%) |
| Atenolol |  | 2 | 3 | 5 |
| Bisoprolol |  | 5 | 3 | 8 |
| Propranolol |  | 2 | 2 | 4 |
| Nitrates, n (%) |  | 1 / 133 (0.8%) | 5 / 131 (3.8%) | 6 / 264 (2.3%) |
| GTN |  | 1 | 4 | 5 |
| Isosorbide mononitrate |  |  | 1 | 1 |
| Thiazide / loop diuretics, n (%) |  | 19 / 133 (14.3%) | 20 / 131 (15.3%) | 39 / 264 (14.8%) |
| Furosemide |  | 17 | 13 | 30 |
| Bendroflumethiazide |  |  | 6 | 6 |
| Bumetanide |  | 1 | 1 | 2 |
| Indapamide |  | 1 |  | 1 |
| Potassium-sparing diuretics, n (%) |  | 30 / 133 (22.6%) | 35 / 131 (26.7%) | 65 / 264 (24.6%) |
| Spironolactone |  | 29 | 33 | 62 |
| Amiloride |  | 1 | 1 | 2 |
| Multiple agents |  |  | 1 | 1 |
| Sildenafil, n (%) |  | 3 / 133 (2.3%) | 0 | 3 / 264 (1.1%) |
| Antibiotics, n (%) |  | 10 / 133 (7.5%) | 6 / 131 (4.6%) | 16 / 264 (6.1%) |
| Rifaximin |  | 6 | 2 | 8 |
| Co-trimoxazole |  |  | 2 | 2 |
| Ciprofloxacin |  | 1 | 1 | 2 |
| Lymecycline |  | 1 |  | 1 |
| Multiple agents |  | 2 | 1 | 3 |

Continued over page ->

|  |  | **Carvedilol**  **(n=133)** | **VBL**  **(n=132)** | **Overall**  **(n=265)** |
| --- | --- | --- | --- | --- |
| **Concomitant medications (continued)** | | | | |
| Antivirals, n (%)* |  | 2 / 116 (1.7%) | 1 / 119 (0.8%) | 3 / 235 (1.3%) |
| Tenofovir |  |  | 1 | 1 |
| Acyclovir |  | 1 |  | 1 |
| Odefsey |  | 1 |  | 1 |
| Ursodeoxycholic acid, n (%)* |  | 6 / 116 (5.2%) | 7 / 119 (5.9%) | 13 / 235 (5.5%) |
| Obeticholic acid, n (%)* |  | 2 / 116 (1.7%) | 0 | 2 / 235 (0.9%) |
| Fibrates, n (%)* |  | 2 / 116 (1.7%) | 0 | 2 / 235 (0.9%) |
| Bezafibrate |  | 1 |  | 1 |
| Gemfibrozil |  | 1 |  | 1 |
| Other, n (%) |  | 88 / 133 (66.2%) | 82 / 131 (62.6%) | 170 / 264 (64.4%) |

Notes:

* - added on version 2 of baseline form

# Adherence to treatment allocation

Adherence to treatment allocation is recorded on the safety visit form and also the 6 and 12 month follow-up forms.

**Table S7**: Treatment adherence by group

|  |  | **Allocated intervention** | |
| --- | --- | --- | --- |
|  |  | **Carvedilol**  **(n=133)** | **VBL**  **(n=132)** |
| **CARVEDILOL** | | | |
| Adherent | >=75%, stayed on carvedilol | 75 |  |
|  | >=75%, stopped for medical reasons | 10 |  |
|  | *Alternate treatment: VBL*  *none stated* | *5*  *5* |  |
| Non-adherent | <75%, stayed on carvedilol | 5 |  |
|  | *Additional treatment: VBL*  *none stated* | *1*  *4* |  |
|  | <75%, stopped for medical reasons | 20 |  |
|  | *Alternate treatment: VBL*  *propranolol*  *none stated* | *14*  *1*  *5* |  |
|  | Percentage adherence to carvedilol missing | 1 |  |
|  | *Additional treatment: VBL* | *1* |  |
|  | >=75%, not stopped for medical reasons, had VBL | 2 |  |
| Adherence unknown | Insufficient data available to determine carvedilol adherence | 20 |  |
| **VBL** | | | |
| Adherent | Had VBL, continued with VBL |  | 52 |
|  | Had VBL, stopped for medical reasons |  | 9 |
|  | *Alternate treatment: carvedilol*  *none stated* |  | *8*  *1* |
| Non-adherent | Had no VBL |  | 24 |
|  | *Alternate treatment: carvedilol*  *none stated* |  | *9*  *15* |
|  | Had VBL, not attended all sessions |  | 11 |
| Adherence unknown | Insufficient data available to determine VBL adherence |  | 36 |
| **OVERALL** | | | |
| **Total adherent population** | **Out of whole trial population** | **85 / 133**  **(64%)** | **61 / 132**  **(46%)** |
|  | **Out of trial population with known adherence status** | **85 / 113**  **(75%)** | **61 / 96**  **(64%)** |

# Alcohol consumption during follow up

## Data for those who answered “no” to minimisation variable for alcohol-related liver disease

**Table S8**: Baseline alcohol consumption – “no” to minimisation variable for alcohol-related liver disease

| **Baseline Carvedilol VBL Overall** | | | | |
| --- | --- | --- | --- | --- |
| (note: where n<randomised then data are missing) |  | **(n=71)** | **(n=70)** | **(n=141)** |
| Alcohol consumed in the past 12 months, n (%) | Yes | 24 (33.8%) | 24 (34.3%) | 48 (34.0%) |
| If alcohol consumed, average units per week | N | 24 | 24 | 48 |
| (note: where N<”yes” then data are missing) | Median [IQR] | 3.5 [1.5, 8] | 3.0 [2, 8] | 3 [2, 8] |
|  | Range | 1, 20 | 1, 30 | 1, 30 |
| If alcohol consumed, AUDIT-C undertaken, n (%) | Yes | 17 / 24 (70.8%) | 17 / 24 (70.8%) | 34 / 48 (70.8%) |
| AUDIT-C score | N | 17 | 17 | 34 |
|  | Median [IQR] | 3 [1, 4] | 2 [1, 4] | 2.5 [1, 4] |
|  | Range | 0, 9 | 0, 10 | 0, 10 |
| Where AUDIT-C undertaken,  score ≥ 5, n (%) | Yes | 3 / 17 (17.7%) | 4 / 17 (23.5%) | 7 / 34 (20.6%) |
| Total AUDIT score | N | 3 | 4 | 7 |
|  | Median [IQR] | 9 [0, 10] | 5.5 [2.5, 8] | 6 [0, 10] |
|  | Range | 0, 10 | 0, 10 | 0, 10 |

**Table S9**: Six-month alcohol consumption – “no” to minimisation variable for alcohol-related liver disease

| **6 months**  **Carvedilol VBL Overall** | | | | |
| --- | --- | --- | --- | --- |
| (note: where n<randomised then data are missing) |  | **(n=65)** | **(n=65)** | **(n=130)** |
| Alcohol consumed in the past 12 months, n (%) | Yes | 24 (36.9%) | 19 (29.2%) | 43 (33.1%) |
| If alcohol consumed, average units per week | N | 23 | 18 | 41 |
| (note: where N<”yes” then data are missing) | Median [IQR] | 4 [1, 9] | 3.5 [1, 10] | 4 [1, 9] |
|  | Range | 1, 14 | 0, 196 | 1, 196 |
| If alcohol consumed, AUDIT-C undertaken, n (%) | Yes | 12 / 24 (50.0%) | 7 / 19 (36.8%) | 19 / 43 (44.2%) |
| AUDIT-C score | N | 12 | 7 | 19 |
|  | Median [IQR] | 2.5 [1, 5] | 2 [1, 6] | 2 [1, 6] |
|  | Range | 0, 9 | 1, 12 | 0, 12 |
| Where AUDIT-C undertaken,  score ≥ 5, n (%) | Yes | 3 / 12 (25.0%) | 2 / 7 (28.6%) | 5 / 19 (26.3%) |
| Where AUDIT-C undertaken,  score ≥ 11, n (%) | Yes | 0 / 12 (0%) | 1 / 7 (14.3%) | 1 / 19 (5.3%) |
| Total AUDIT score | N | 3 | 2 | 5 |
|  | Median [IQR] | 7 [0, 9] | 14 [6, 22] | 7 [6, 9] |
|  | Range | 0, 9 | 6, 22 | 0, 22 |
| Where total AUDIT-C undertaken,  score ≥ 20, n (%) | Yes | 0 / 3 (0%) | 1 / 2 (50%) | 1 / 5 (20.0%) |

**Table S10**: 12-month alcohol consumption – “no” to minimisation variable for alcohol-related liver disease

| **12 months Carvedilol VBL Overall** | | | | |
| --- | --- | --- | --- | --- |
| (note: where n<randomised, then data are missing) |  | **(n=64)** | **(n=58)** | **(n=122)** |
| Alcohol consumed in the past 12 months, n (%) | Yes | 19 (29.7%) | 13 (22.4%) | 32 (26.2%) |
| If alcohol consumed, average units per week | N | 18 | 12 | 30 |
| (note: where N<”yes” then data are missing) | Median [IQR] | 4 [2, 10] | 3.5 [1, 10] | 4 [2, 10] |
|  | Range | 1, 24 | 0, 22 | 0, 24 |
| If alcohol consumed, AUDIT-C undertaken, n (%) | Yes | 8 / 19 (42.1%) | 3 / 13 (23.1%) | 11 / 32 (34.4%) |
| AUDIT-C score | N | 8 | 3 | 11 |
|  | Median [IQR] | 3 [2, 4] | 1 [1, 1] | 2 [1, 4] |
|  | Range | 1, 4 | 1, 1 | 1, 4 |
| Where AUDIT-C undertaken,  score ≥ 5, n (%) | Yes | 0 / 8 (0%) | 0 / 3 (0%) | 0 / 11 (0%) |
| Where AUDIT-C undertaken,  score ≥ 11, n (%) | Yes | 0 / 8 (0%) | 0 / 3 (0%) | 0 / 11 (0%) |
| Total AUDIT score | N | - | - | - |
|  | Median [IQR] | - | - | - |
|  | Range | - | - | - |
| Where total AUDIT-C undertaken,  score ≥ 20, n (%) | Yes | - | - | - |

## Data for those who answered “yes” to minimisation variable for

## alcohol-related liver disease

 **Table S11**: Baseline alcohol consumption – “yes” to minimisation variable for alcohol-related liver disease

| **Baseline Carvedilol VBL Overall** | | | | |
| --- | --- | --- | --- | --- |
| (note: where n<randomised then data are missing) |  | **(n=62)** | **(n=61)** | **(n=123)** |
| Alcohol consumed in the past 12 months, n (%) | Yes | 33 (53.2%) | 42 (68.9%) | 75 (61.00%) |
| If alcohol consumed, average units per week | N | 29 | 40 | 69 |
| (note: where N<”yes” then data are missing) | Median [IQR] | 30 [10, 60] | 49 [17, 85] | 40 [14, 80] |
|  | Range | 1, 200 | 2, 280 | 1, 280 |
| If alcohol consumed, AUDIT-C undertaken, n (%) | Yes | 22 / 33 (66.7%) | 33 / 42 (78.6%) | 55 / 123 (73.3%) |
| AUDIT-C score | N | 22 | 33 | 55 |
|  | Median [IQR] | 9 [4, 12] | 10 [5, 12] | 10 [4, 12] |
|  | Range | 0, 12 | 3, 12 | 0, 12 |
| Where AUDIT-C undertaken,  score ≥ 5, n (%) | Yes | 16 / 22 (72.7%) | 25 / 33 (75.8%) | 41 / 55 (74.6%) |
| Total AUDIT score | N | 16 | 25 | 41 |
|  | Median [IQR] | 21 [16, 25] | 17 [12, 21] | 19 [13, 23] |
|  | Range | 7, 36 | 8, 32 | 7, 36 |

**Table S12**: Six month alcohol consumption – “yes” to minimisation variable for alcohol-related liver disease

| **6 months Carvedilol VBL Overall** | | | | |
| --- | --- | --- | --- | --- |
| (note: where n<randomised then data are missing) |  | **(n=52)** | **(n=51)** | **(n=103)** |
| Alcohol consumed in the past 12 months, n (%) | Yes | 18 (34.6%) | 28 (54.9%) | 46 (44.7%) |
| If alcohol consumed, average units per week | N | 16 | 24 | 40 |
| (note: where N<”yes” then data are missing) | Median [IQR] | 9 [3, 60] | 15 [5, 33] | 14.5 [4.5, 43] |
|  | Range | 0, 210 | 1, 269 | 0, 269 |
| If alcohol consumed, AUDIT-C undertaken, n (%) | Yes | 9 / 18 (50.0%) | 17 / 28 (60.7%) | 26 / 46 (56.5%) |
| AUDIT-C score | N | 9 | 17 | 26 |
|  | Median [IQR] | 8 [4, 12] | 5 [3, 9] | 6.5 [3, 9] |
|  | Range | 0, 12 | 0, 12 | 0, 12 |
| Where AUDIT-C undertaken,  score ≥ 5, n (%) | Yes | 6 / 9 (66.7%) | 9 / 17 (52.9%) | 15 / 26 (57.7%) |
| Where AUDIT-C undertaken,  score ≥ 11, n (%) | Yes | 3 / 9 (33.3%) | 2 / 17 (11.8%) | 5 / 26 (19.2%) |
| Total AUDIT score | N | 6 | 9 | 15 |
|  | Median [IQR] | 17.5 [14, 18] | 12 [9, 14] | 14 [12, 18] |
|  | Range | 13, 30 | 7, 29 | 7, 30 |
| Where total AUDIT-C undertaken,  score ≥ 20, n (%) | Yes | 1 / 6 (16.7%) | 2 / 9 (22.2%) | 3 / 15 (20.0%) |

**Table S13**: 12-month alcohol consumption – “yes” to minimisation variable for alcohol-related liver disease

| **12 months Carvedilol VBL Overall** | | | | |
| --- | --- | --- | --- | --- |
| (note: where n<randomised then data are missing) |  | **(n=48)** | **(n=47)** | **(n=95)** |
| Alcohol consumed in the past 12 months, n (%) | Yes | 15 (31.3%) | 21 (44.7%) | 36 (37.9%) |
| If alcohol consumed, average units per week | N | 13 | 20 | 33 |
| (note: where N<”yes” then data are missing) | Median [IQR] | 8 [2, 30] | 14.5 [4, 32.5] | 14 [3, 30] |
|  | Range | 1, 140 | 1, 280 | 1, 280 |
| If alcohol consumed, AUDIT-C undertaken, n (%) | Yes | 8 / 15 (53.3%) | 15 / 21 (71.4%) | 23 / 36 (63.9%) |
| AUDIT-C score | N | 8 | 15 | 23 |
|  | Median [IQR] | 4.5 [1.5, 9.5] | 6 [4, 8] | 6 [2, 8] |
|  | Range | 0, 12 | 1, 12 | 0, 12 |
| Where AUDIT-C undertaken,  score ≥ 5, n (%) | Yes | 4 / 8 (50.0%) | 9 / 15 (60.0%) | 13 / 23 (56.5%) |
| Where AUDIT-C undertaken,  score ≥ 11, n (%) | Yes | 2 / 8 (25.0%) | 2 / 15 (13.3%) | 4 / 23 (17.4%) |
| Total AUDIT score | N | 4 | 9 | 13 |
|  | Median [IQR] | 15.5 [14, 20] | 14 [12, 23] | 14 [13, 23] |
|  | Range | 14, 23 | 5, 24 | 5, 24 |
| Where total AUDIT-C undertaken,  score ≥ 20, n (%) | Yes | 1 / 4 (25.0%) | 4 / 9 (44.4%) | 5 / 13 (38.5%) |

# Analysis of secondary outcome measures

Events forming the secondary outcomes in this section are normally identified on the 6 month and 12 month follow-up forms. If a participant has at least one event for the relevant outcome (e.g. at least one episode of variceal bleeding for time to first variceal bleeding) indicated on either of these follow-up forms then they have experienced the event in question. Participants which do not fulfil this criteria for will be termed “event-free”.

If a participant is termed as having experienced the event then their time until event will be defined as the number of days from the date of randomisation till the date of the first event.

Participants who are termed as event-free and who were last known to be event-free prior to the 12-month follow-up time-point will censored in the analysis. The time to censoring will be the number of days from the date of randomisation to the date last known to be event-free. This will be determined by the visit date of the most recent trial form received e.g. date of completion on the 12-month follow-up form. If the last known date in the trial is equal to the date of randomisation then the participant will be censored at day 1.

**Risk Ratio / Risk Difference Models**

The risk ratio / risk difference models for each outcome only include participants for whom complete 12-month data is available ^α^. By definition, participants with an event provide complete data. If the censored time 305≤ x ≤ 365 days then the participant is deemed to have complete data. If the censored time is ≤ 305 then there is not complete information within 1 year for this participant. Therefore, they are not able to be included in the risk ratio / risk difference models.

**Cox Models**

The Cox models for each outcome include all randomised participants. Participants who have not reported an event are censored at the date of their last follow-up form (or at day 1 as described above).

^α^ : *note that, in the SAP, there is an error with regards the window of completion for the 12-month time-point. The SAP implies a 2-week window (365 -351 days) instead of a 2-month window, as per the current and all previous versions the protocol. Assuming a month length of 30 days this equates to 60 days and therefore follow-up forms with visit dates at least 365 – 60 = 305 days after randomisation will be considered as complete 1-year data for the risk ratio and risk difference models.*

**Planned analysis in SAP:**

Time to event outcomes will be compared between treatment groups using standard survival analysis methods. Kaplan-Meier survival curves will be constructed for visual presentation of time-to-event comparisons. Cox proportional hazard models will be fitted to obtain adjusted treatment effects which will be expressed as hazard ratios with 95% confidence intervals.

**Deviations from the planned analysis in SAP:**

1) As previously mentioned, the planned analysis models for the primary outcome incorporating adjustment for minimisation variables failed to converge due to the small number of events. In light of this, all primary and secondary outcomes will be presented using unadjusted models instead of adjusting for minimisation variables.

2) Due to the problems caused by the pandemic, assessments may not have been completed at exactly the correct time-point. The SAP described the perfect-world situation where data is considered missing if the 12-month follow-up CRF was not returned during the window of assessment. However, in the real-world it is possible that a late 6-month visit, for example, may give sufficient information at the 305 day time-point. To best use all of the collected data, the time-to-assessment is used to determine the censoring date in all cases, instead of first selecting on whether a 12-month form has been returned.

## Mortality within 1 Year of Randomisation

**Figure S2**: Kaplan Meier: overall mortality





Note: the percentages at the end of a Kaplan-Meier plot are commonly a little different to the percentages obtained from the raw numbers. This is due to the way the method accounts for the censored data when participants do not provide complete information.

## Transplant-Free Survival

**Figure S3**: Kaplan Meier: transplant free survival





Note: the percentages at the end of a Kaplan-Meier plot are commonly a little different to the percentages obtained from the raw numbers. This is due to the way the method accounts for the censored data when participants do not provide complete information.

## Other Complications of Cirrhosis (within 1 year of randomisation)

Data regarding complications of cirrhosis are collected on the 6 and 12 month follow-up forms. The time to event and time to censoring are calculated as per the descriptions for the previous outcomes.

**Planned analysis in SAP:**

These outcomes will be analysed and reported in the same way as all-cause mortality. The number and percentage of participants who have developed the complication within one year of randomisation will be reported by treatment group. An adjusted risk ratio and 95% confidence interval will be estimated from a log-binomial regression model. Risk difference will also be reported.

**Additional deviations from the planned analysis in SAP:**

1) An analysis using a Cox proportional hazards model is also carried out using the same methods as described for time to first variceal bleed. This is to better use the small amount of data available.

### New Onset Ascites

**Figure S4**: Kaplan Meier: New Onset Ascites





Note: the percentages at the end of a Kaplan-Meier plot are commonly a little different to the percentages obtained from the raw numbers. This is due to the way the method accounts for the censored data when participants do not provide complete information.

### Spontaneous Bacterial Peritonitis

**Figure S5**: Kaplan Meier: Spontaneous Bacterial Peritonitis

**

**

Note: the percentages at the end of a Kaplan-Meier plot are commonly a little different to the percentages obtained from the raw numbers. This is due to the way the method accounts for the censored data when participants do not provide complete information.

### Hepatocellular Carcinoma

**Figure S6**: Kaplan Meier: Hepatocellular Carcinoma

**

**

Note: the percentages at the end of a Kaplan-Meier plot are commonly a little different to the percentages obtained from the raw numbers. This is due to the way the method accounts for the censored data when participants do not provide complete information.

### Hepatic Encephalopathy

**Figure S7**: Kaplan Meier: Hepatic Encephalopathy

**

**

Note: the percentages at the end of a Kaplan-Meier plot are commonly a little different to the percentages obtained from the raw numbers. This is due to the way the method accounts for the censored data when participants do not provide complete information.

# Analysis of exploratory outcome measures

## Hepatic Decompensation

A variety of the outcomes analysed in Section 8.3 (other complications of cirrhosis) can be combined into a composite variable termed “Hepatic Decompensation”. There are two competing schools of thought as to which outcomes should be contained in the composite:

Definition 1 - Variceal bleeding, new onset ascites, encephalopathy

Definition 2 - Variceal bleeding, new onset ascites, encephalopathy, bacterial peritonitis

The composite outcome will be obtained using the same methods described for the individual analyses in Section 8.3.

A participant will be deemed to have experienced hepatic decompensation if they experience one or more of the contributing individual outcomes. The first of such events will be deemed the date of hepatic decompensation. Participants not experiencing hepatic decompensation will be censored at the date of last follow-up (or at 1 day is no follow-up is available). Participants will be deemed to have complete follow-up if they have more than 305 days of follow-up, as per the original analyses.

There are two populations of interest for both definitions:

- Whole population
- Those participants who were not already decompensated at the point of randomisation.

Note that, as per the SAP, the results of exploratory analyses will be descriptive in nature and no hypothesis testing will be carried out.

### Hepatic Decompensation – definition 1

Contributing events: variceal bleeding, new onset ascites, encephalopathy

**Table S14**: Analysis of hepatic decompensation within 1 year of randomisation – definition 1 (entire population)

|  | **Carvedilol**  **(n=133)** | **VBL**  **(n=132)** |
| --- | --- | --- |
| Data available: |  |  |
| Known decompensation before 1 year | 15 | 21 |
| *Bleeding* | *2* | *3* |
| *Ascites* | *4* | *6* |
| *HE* | *5* | *4* |
| *Bleeding and ascites* |  | *4* |
| *Bleeding and HE* | *1* | *3* |
| *Ascites and HE* | *1* | *1* |
| *Bleeding, ascites and HE* | *2* |  |
|  |  |  |
| No decompensation with complete FU data to 1 year | 70 | 69 |
| No decompensation and between 305-364 days of FU | 29 | 19 |
| No decompensation and between 180-304 days of FU | 6 | 9 |
| No decompensation and between 150-179 days of FU | 2 | 0 |
| No decompensation and between 100-149 days of FU | 0 | 1 |
| No FU | 11 | 13 |
|  |  |  |
| Decompensation within 1 year of randomisation | 15 / 114  (13.2%) | 21 / 109  (19.3%) |
|  | 15 / 133  (11.3%) | 21 / 132  (15.9%) |

Note: the green rows in the table indicate participants who have “complete” follow-up by virtue of being within the 2-month window of the 1-yr time-point. The red rows indicate participants who do not have “complete” follow-up.

**Table S15**: Analysis of hepatic decompensation within 1 year of randomisation – definition 1 (compensated disease at randomisation)

|  | **Carvedilol**  **(n=133)** | **VBL**  **(n=132)** |
| --- | --- | --- |
| Decompensated at randomisation |  |  |
| No | 101 (75.9%) | 100 (75.8%) |
| Yes | 32 (24.1%) | 32 (24.2%) |
|  |  |  |
|  | **Carvedilol**  **(n=101)** | **VBL**  **(n=100)** |
| Data available: |  |  |
| Known decompensation before 1 year | 12 | 9 |
| *Bleeding* | *1* |  |
| *Ascites* | *3* | *2* |
| *HE* | *5* | *2* |
| *Bleeding and ascites* |  | *3* |
| *Bleeding and HE* |  | *1* |
| *Ascites and HE* | *1* | *1* |
| *Bleeding, ascites and HE* | *2* |  |
|  |  |  |
| No decompensation with complete FU data to 1 year | 52 | 58 |
| No decompensation and between 305-364 days of FU | 25 | 18 |
| No decompensation and between 180-304 days of FU | 5 | 6 |
| No decompensation and between 150-179 days of FU | 1 | 0 |
| No FU | 6 | 9 |
|  |  |  |
| Decompensation within 1 year of randomisation | 12 / 89  (13.5%) | 9 / 85  (10.6%) |
|  | 12 / 101  (11.9%) | 9 / 100  (9.0%) |

Note: the green rows in the table indicate participants who have “complete” follow-up by virtue of being within the 2-month window of the 1-yr time-point. The red rows indicate participants who do not have “complete” follow-up.

### Hepatic Decompensation – definition 2

Contributing events: variceal bleeding, new onset ascites, encephalopathy, bacterial peritonitis

**Table S16**: Analysis of hepatic decompensation within 1 year of randomisation – definition 2 (entire population)

|  | **Carvedilol**  **(n=133)** | **VBL**  **(n=132)** |
| --- | --- | --- |
| Data available: |  |  |
| Known decompensation before 1 year | 16 | 22 |
| *Bleeding* | *2* | *3* |
| *Ascites* | *4* | *5* |
| *HE* | *4* | *3* |
| *SBP* | *1* | *1* |
| *Bleeding and ascites* |  | *4* |
| *Bleeding and HE* | *1* | *3* |
| *Bleeding and SBP* |  |  |
| *Ascites and HE* | *1* | *1* |
| *Ascites and SBP* |  | *1* |
| *HE and SBP* | *1* | *1* |
| *Bleeding, ascites and HE* | *2* |  |
| *Bleeding, ascites, and SBP* |  |  |
| *Bleeding, HE and SBP* |  |  |
| *Ascites, HE and SBP* |  |  |
| *Bleeding, ascites, HE and SBP* |  |  |
|  |  |  |
| No decompensation with complete FU data to 1 year | 70 | 68 |
| No decompensation and between 305-364 days of FU | 29 | 19 |
| No decompensation and between 180-304 days of FU | 5 | 9 |
| No decompensation and between 150-179 days of FU | 2 | 0 |
| No decompensation and between 100-149 days of FU | 0 | 1 |
| No FU | 11 | 13 |
|  |  |  |
| Decompensation within 1 year of randomisation | 16 / 115  (13.9%) | 22 / 109  (20.2%) |
|  | 16 / 133  (12.0%) | 22 / 132  (16.7%) |

Note: the green rows in the table indicate participants who have “complete” follow-up by virtue of being within the 2-month window of the 1-yr time-point. The red rows indicate participants who do not have “complete” follow-up.

**Table S17**: Analysis of hepatic decompensation within 1 year of randomisation – definition 2 (compensated disease at randomisation)

|  | **Carvedilol**  **(n=133)** | **VBL**  **(n=132)** |
| --- | --- | --- |
| Decompensated at randomisation |  |  |
| No | 101 (75.9%) | 100 (75.8%) |
| Yes | 32 (24.1%) | 32 (24.2%) |
|  |  |  |
|  | **Carvedilol**  **(n=101)** | **VBL**  **(n=100)** |
| Data available: |  |  |
| Known decompensation before 1 year | 13 | 9 |
| *Bleeding* | *1* |  |
| *Ascites* | *3* | *1* |
| *HE* | *4* | *1* |
| *SBP* | *1* |  |
| *Bleeding and ascites* |  | *3* |
| *Bleeding and HE* |  | *1* |
| *Bleeding and SBP* |  |  |
| *Ascites and HE* | *1* | *1* |
| *Ascites and SBP* |  | *1* |
| *HE and SBP* | *1* | *1* |
| *Bleeding, ascites and HE* | *2* |  |
| *Bleeding, ascites, and SBP* |  |  |
| *Bleeding, HE and SBP* |  |  |
| *Ascites, HE and SBP* |  |  |
| *Bleeding, ascites, HE and SBP* |  |  |
|  |  |  |
| No decompensation with complete FU data to 1 year | 52 | 58 |
| No decompensation and between 305-364 days of FU | 25 | 18 |
| No decompensation and between 180-304 days of FU | 4 | 6 |
| No decompensation and between 150-179 days of FU | 1 | 0 |
| No FU | 6 | 9 |
|  |  |  |
| Decompensation within 1 year of randomisation | 13 / 90  (14.4%) | 9 / 85  (10.6%) |
|  | 13 / 101  (12.9%) | 9 / 100  (9.0%) |

Note: the green rows in the table indicate participants who have “complete” follow-up by virtue of being within the 2-month window of the 1-yr time-point. The red rows indicate participants who do not have “complete” follow-up.

# Safety – Serious Adverse Events

There were a total of 2 SAEs in 2 participants which were deemed to be definitely or probably related to treatment. Details on these SAEs can be found in Table S18.

**Table S18:** Details of SAEs by group

|  | **CTCAE body system** | **Description** | **Site causality assessment** | **Expectedness** |
| --- | --- | --- | --- | --- |
| **Carvedilol** | | | |  |
| 1 | Vascular disorders | Postural hypotension secondary to medications for varices | Definitely related | Expected |
| **VBL** | | | |  |
| 2 | Gastrointestinal disorders | Pain and vomiting post-banding | Definitely related | Expected |

# Economic evaluation

Methods

Within-trial cost-utility and cost-effectiveness analyses were conducted from the perspective of the UK National Health Service (NHS) over a 12 month period. Patient-level data on healthcare resource use required for carvedilol and VBL interventions were collected at 6 months and 12-months. This included trial interventions, surveillance endoscopy, hospital admissions and outpatient appointments for treating liver complications, primary care contacts and investigations and diagnostic imaging. The cost of VBL was estimated for each patient in the VBL arm from the data collected on the number of endoscopies with band ligation. Where patients in the carvedilol arm had band ligation, this cost was considered as an additional management cost. Unit cost values were obtained from national sources, including the costs of non-selective beta-blockers (NSBBs) from BNF^1^ while the costs of VBL sessions were derived from NICE guidance^2^. Hospital costs were primarily sourced from NHS Reference costs^3^, and primary healthcare costs were obtained from the Personal Social Services Research Unit (PSSRU) Unit Costs of Health and Social Care^4^. All costs were valued at 2023 prices (**Table S14**).

The clinical effectiveness outcome was variceal bleeding avoided, derived from the clinical primary outcome of the trial. For the cost-utility analysis (CUA) quality-adjusted life years (QALYs) were calculated for each participant using the EQ-5D-5L. Following NICE recommendations, patient specific responses to the EQ-5D-5L questionnaire at baseline, 6 months, and 12 months were converted to EQ-5D-3L UK index scores via a mapping algorithm developed by Decision Support Unit and using the 'EEPRU dataset'^5^. QALYs were generated using the area under the curve method^6^.

Total costs and benefits, including variceal bleeding avoided and Quality-Adjusted Life Years (QALYs), associated with each intervention were compared to generate incremental cost-effectiveness ratios (ICERs). According to NICE guidelines, an intervention is considered cost-effective if the ICER is less than £20,000–30,000 per QALY gained^7^. The outcomes of the cost-effectiveness analyses (CEAs) were expressed as the cost per variceal bleeding avoided within one year of randomization and for the CUA, the economic outcome was cost per QALY.

Although the protocol and analysis plan specified that multiple imputation would be used to replace missing data, due to high levels of missing EQ-5D-5L data, a complete case analysis was undertaken, using complete health economic data on resource use, EQ-5D-5L and the primary outcome.

Adjustments for baseline differences were made where imbalances in baseline utility (EQ-5D-5L) scores and other covariates between the study arms were controlled for with a multiple linear regression approach.^6, 8^ A bootstrapping approach with replacement was employed, and mean incremental costs and outcomes were reported as the mean differences along with their 95% confidence intervals (2.5th and 97.5th percentiles) around the mean. An ICER was not calculated because the intervention (Carvedilol) was dominant, and a negative ICER is not considered relevant for decision-making. To account for overall uncertainty, bootstrapping was used to generate 5,000 paired estimates of mean differential costs and QALYs. The bootstrapped values of costs and effects were graphically presented on a cost-effectiveness plane,^9, 10^ and cost-effectiveness acceptability curves (CEACs) were constructed to show the probability of cost-effectiveness of interventions at different willingness-to-pay thresholds^11^. The analyses were conducted using Stata 18 software^12^.

Scenario analysis was conducted to investigate whether the cost-effectiveness changed if a follow up GP appointment was implemented as part of carvedilol treatment routine and if the crosswalk EQ-5D-5L mapping method was used to generate QALYs^13^.

Results

Carvedilol was less costly primarily due to lower intervention costs, reduced need for surveillance endoscopies, and slightly lower costs associated with managing liver complications (ascites, variceal bleeding, and spontaneous bacterial peritonitis). For the complete case analysis for resource use and costs, carvedilol was cost saving, with the difference in costs -£1,608 (-£,3426 to £104). Detailed information on resource use and costs is provided in (Table S15,Table S16,Table S17)

EQ-5D-5L scores were higher for the carvedilol at baseline and the difference increased at 6 months to 0.016 (-00.060 to 0.090) (Table S18a-c). After adjustment for baseline covariates, carvedilol was slightly more effective (Table S18b) with 0.010 (95%CI, -0.038 to 0.056) additional QALYs. The 95% confidence interval crossed zero, indicating that the difference was not significant. The adjusted QALY difference using the crosswalk approach again favoured carvedilol and was similar at 0.013(-0.037 to 0.060) QALYs.

The base case results of the cost utility analysis (Table S19), where patients with complete resource use and EQ-5D-5L data were considered, suggested that carvedilol was likely to be cost-saving (mean, -£1,053; 95% CI, -£2,098 to £14.80) with a slight improvement in QALYs. Therefore, carvedilol dominated VBL. The cost-effectiveness plane in **Figure S8** had points in all four quadrants, and the CEAC in **Figure S9** shows carvedilol had a 92% probability of being cost-effective compared to VBL at the £20,000 per QALY threshold. The cost-effectiveness analysis, using complete resource use and clinical outcome data, showed that carvedilol avoided slightly more bleeds and resulted in a cost saving of -£1,608 (95% CI, -£3,426 to £104). The findings remained consistent across different scenarios, with carvedilol continuing to be a cost-saving option (Table S20).

**Table S20:** Resource Use Prices and description (price year 2023)

|  | **Unit cost** | **Description** | **Source** |
| --- | --- | --- | --- |
| **Intervention** | | | |
| Carvedilol 12.5mg (per tablet) | £0.05 | £1.32 Pack of 28 tablets  Once daily dose of 12.5mg | BNF 2023 |
| Variceal band ligation session | £680 | Original source was an NHS hospital trust where a member of the NICE committee worked (original value from 2020) | NICE guidance^2^ |
| **Secondary care (hospital-based) services** | | | |
| **Hospital stay related to adverse events (per episode otherwise stated)** | | | |
| Variceal bleeding episode | £4,820 |  | Mattock et al. (2021)^14^ |
| Ascites | £1,198 | Ascites managed in an outpatient setting | Mattock et al. (2021)^14^ |
|  | £385 | Cost per day if treated in an inpatient setting/ KC05G – KC05N (Fluid or Electrolyte Disorders) | Mattock et al. (2021)^14^ |
| Spontaneous Bacterial Peritonitis | £1,633 |  | Mattock et al. (2021)^14^ |
| Renal dysfunction | £3,197 | Weighted average - General Renal Disorders with/without Interventions | NHS Reference Costs 21/22^3^ |
| Hepatocellular carcinoma | £5,558 |  | Cullen et al. (2023)^15^ |
| Hepatic Encephalopathy | £1,719 |  | Mattock et al. (2021)^14^ |
| **Therapeutic procedures** |  |  |  |
| Liver transplant | £28,560 | Liver transplant GA15A, Elective admission | NHS Reference Costs 21/22^3^ |
| **Outpatient care** |  |  |  |
| Outpatient appointment | £200.45 | Weighted average of consultant and non-consultant led outpatient care – hepatology service (WF01A), non-admitted Face-to-Face attendance, follow-up | NHS Reference Costs 21/22^3^ |
| **Diagnostic procedure/imaging or test (per visit)** |  |  |  |
| Endoscopy | £634 | FE22Z (Diagnostic Endoscopic Upper Gastrointestinal Tract Procedures, 19 years and over) – day case | NHS Reference Costs 21/22^3^ |
| Ultrasound | £73 | Ultrasound Scan with a duration of less than 20 minutes, without Contrast | NHS Reference Costs 21/22^3^ |
| Fibroscan | £94 | Ultrasound Elastography (RD48Z) | NHS Reference Costs 21/22^3^ |
| ECG | £284 | FY51Z (Electrocardiogram Monitoring or Stress Testing/Gastroenterology Service) - outpatient procedure | NHS Reference Costs 21/22^3^ |
| **Primary care (community-based) services** | | | |
| GP visit | £56 | 10 minutes contact including direct care staff cost with qualification | PSSRU 2023^4^ |
| ECG, electrocardiogram; GP, General Practitioner; PSSRU, Personal Social Services Research Unit  *Prices from previous years were adjusted to 2023 | | | |

**Table S21:** Mean healthcare resource use per patient over 12 months - complete follow up data

|  |  |  | **Carvedilol**  **Mean Unit (SD)**  **n=109** |  |  | **VBL**  **Mean Unit (SD)**  **n=105** |
| --- | --- | --- | --- | --- | --- | --- |
| **Primary healthcare services** |  |  |  |  |  |  |
| GP visits |  |  | 0.34 (1.14) |  |  | 0.30 (1.07) |
| **Secondary healthcare (hospital based) services** | | | | | | |
| **Intervention** |  |  |  |  |  |  |
| Band ligation (number of sessions) |  |  | - |  |  | 1.85 (0.92) |
| **Investigations/treatments** |  |  |  |  |  |  |
| Additional endoscopy with banding for carvedilol patients (number of sessions) |  |  | 0.20 (0.59) |  |  | - |
| Surveillance endoscopy (number of sessions) |  |  | 0.17 (0.49) |  |  | 1.00 (0.94) |
| Ultrasound |  |  | 1.18 (0.68) |  |  | 1.16 (0.76) |
| Fibroscan |  |  | 0.02 (0.13) |  |  | 0.03 (0.17) |
| ECG |  |  | 0.07 (0.33) |  |  | 0.08 (0.30) |
| Therapeutic procedures |  |  |  |  |  |  |
| Liver transplant |  |  | 0.05 (0.21) |  |  | 0.04 (0.19) |

ECG, electrocardiogram; GP, General Practitioner; n, number of participants, SD, Standard

Deviation; VBL, Variceal Band Ligation

**Table S22:** Hospital Services Consumption over 12 month period - Number of participants from complete resource use data

| **Events** |  | **Carvedilol**  **n=109** |  | **VBL**  **n=105** |
| --- | --- | --- | --- | --- |
| Additional surveillance endoscopy |  |  |  |  |
| *With banding* |  | 15 (14%) |  | 0 (0%) |
| *Without banding* |  | 14 (13%) |  | 105* (100%) |
| Liver transplant |  | 5 (5%) |  | 4 (4%) |
| Liver complications |  |  |  |  |
| *Ascites new or worsening* |  | 12 (11%) |  | 18 (17%) |
| *Spontaneous Bacterial Peritonitis* |  | 1 (1%) |  | 3 (3%) |
| *Hepatorenal Syndrome* |  | 1 (1%) |  | 0 (0%) |
| *Hepatocellular Carcinoma* |  | 2 (2%) |  | 2 (2%) |
| *Hepatic Encephalopathy* |  | 10 (9%) |  | 7 (7%) |

VBL, Variceal Band Ligation

*an average of 2 sessions was used for patients randomised to VBL where the number of VBL sessions was not reported.

**Table S23:** Mean aggregated cost over 12 months period for all cost categories (2023 prices- GBP) – complete resource use data

| **Cost categories** |  |  | **Carvedilol**  **n=107** |  | **VBL**  **n=101** |  | **Bootstrapped difference ^a^ (95% CI)** |
| --- | --- | --- | --- | --- | --- | --- | --- |
|  |  |  | **Mean (£) cost (SD)** |  | **Mean (£) cost (SD)** |  |  |
| Intervention |  |  | 18.00 |  | 1,256.38 (623.95) |  | -1,236.53 (-1,366.53 to -1,128.31) |
| Additional endoscopy with banding for carvedilol patients |  |  | 137.25 (401.01) |  | 0 |  | 137.71 (73.15 to 225.63) |
| Surveillance endoscopy without banding |  |  | 110.51 (309.20) |  | 634.00 (596.30) |  | -522.06 (-652.77 to -400.25) |
| Liver transplants |  |  | 1,393.17 (6,383.16) |  | 1,156.99 (5,841.69) |  | 211.89 (-1,358.69 to 1,814.65) |
| Primary healthcare services |  |  | 19.01 (63.86) |  | 17.07 (59.71) |  | 1.85 (-15.44 to 17.24) |
| Investigations |  |  | 108.96 (109.64) |  | 109.14 (106.05) |  | -0.27 (-30.04 to 28.11) |
| Managing liver complications |  |  |  |  |  |  |  |
| *Ascites new or worsening* |  |  | *148.32 (915.57)* |  | *498.59 (1,754.47)* |  | *-354.15 (-788.07 to -14.03)* |
| *Spontaneous Bacterial Peritonitis* |  |  | *14.98 (156.41)* |  | *77.76 (525.31)* |  | *-62.94 (-200.00 to 15.59)* |
| *Hepatorenal Syndrome* |  |  | *117.32 (1,224.87)* |  | *0* |  | *118.67 (0 to 429.28)* |
| *Hepatocellular Carcinoma* |  |  | *101.98 (749.38)* |  | - 1. *(604.09)* |  | *-25.16 (-150.94 to 224.00)* |
| *Hepatic Encephalopathy* |  |  | *236.56 (825.99)* |  | *163.71 (653.87)* |  | *72.63 (-124.69 to 282.57)* |
| Sub-total (managing liver complications) |  |  | 619.17 (2,011.68) |  | 819.47 (2,248.43) |  | -200.63 (-808.61 to 349.10) |
| **Total NHS costs** |  |  | **2,406.06 (6,828.82)** |  | **3,993.05 (6,753.67)** |  | **-1,608.04 (-3,426.23 to 103.53)** |

CI, Confidence Interval; n, number of participants; SD, Standard Deviation; VBL, Variceal Bleeding Ligation.

^a^Adjusted for baseline age, the presence or absence of hepatic decompensation, size of the largest varix, presence or absence of alcohol related liver disease. Values <0 favour carvedilol in comparison to VBL

|  | **Carvedilol**  **n=133** |  | **VBL**  **n=132** |
| --- | --- | --- | --- |
| Baseline |  |  |  |
| Complete, n (%) | 123 (92%) |  | 122 (92%) |
| Missing, n (%) | 10 (8%) |  | 10 (8%) |
| 6 months |  |  |  |
| Complete, n (%) | 88 (66%) |  | 86 (65%) |
| Missing, n (%) | 45 (34%) |  | 46 (35%) |
| 12 months |  |  |  |
| Complete, n (%) | 86 (65%) |  | 84 (64%) |
| Missing, n (%) | 47 (35%) |  | 48 (36%) |
| **All follow up points** |  |  |  |
| **Complete, n (%)** | **73 (55%)** |  | **68 (52%)** |
| **Missing, n (%)** | **60 (45%)** |  | **64 (48%)** |

**Table S24:** Number and percentage of participants with complete and missing EQ-5D-5L Data

EQ-5D-5L, EuroQol 5-domain instrument 5-level; n, number of participants; VBL, Vein Bypass Ligation.

Note: EQ-5D-5L data was considered missing if any or all the questionnaire domains were incomplete.

**Table S25:** EQ-5D-5L index score and calculated Quality Adjusted Life Years - 12 months post randomisation (complete case data).

|  | **Carvedilol**  **n=72** | **VBL**  **n=68** | **Bootstrapped difference ^a^** |
| --- | --- | --- | --- |
|  | **Mean (£) cost (SD)** | **Mean (£) cost (SD)** |  |
| **EQ-5D-5L index score – base case - DSU approach** | | | |
| Baseline | 0.750 (0.239) | 0.744 (0.246) | 0.010 (-0.074 to 0.088) |
| 6 months | 0.709 (0.284) | 0.677 (0.292) | 0.016 (-0.060 to 0.090) |
| 12 months | 0.690 (0.270) | 0.673 (0.282) | 0.009 (-0.061 to 0.079) |
| **Adjusted QALYs** | **0.714 (0.238)** | **0.693 (0.248)** | **0.010 (-0.038 to 0.056)** |
| Unadjusted QALYs |  |  | 0.021 (-0.058 to 0.102) |
| **EQ-5D-5L index score – crosswalk approach** | | | |
| Baseline | 0.749 (0.246) | 0.754 (0.232) | -0.004 (-0.087 to 0.072) |
| 6 months | 0.711 (0.281) | 0.683 (0.286) | 0.020 (-0.056 to 0.039) |
| 12 months | 0.693 (0.272) | 0.682 (0.269) | 0.012 (-0.058 to 0.087) |
| **Adjusted QALYs** | **0.716 (0.240)** | **0.701 (0.235)** | **0.013 (-0.037 to 0.060)** |
| Unadjusted QALYs |  |  | 0.015 (-0.061 to 0.094) |

DSU, Decision Support Unit; QALY, Quality Adjusted Life Year; n, number of participants; VBL, Variceal Bleeding Ligation.

^a^Adjusted for baseline EQ-5D-5L score, age, the presence or absence of hepatic decompensation, size of the largest varix, presence or absence of alcohol related liver disease. Values >0 favour carvedilol in comparison to VBL

Note: missing values for those who died due to liver disease were replaced with zero.

**Table S26:** EQ-5D-5L VAS scores per participant over 12 months

| Health utility | Carvedilol | VBL | Mean bootstrapped difference^a^ (95% CI) |
| --- | --- | --- | --- |
| **EQ-5D-5L Baseline** | | | |
| n | 125 | 124 |  |
| Mean VAS score (SD) | 67.53 (21.13) | 69.33 (19.08) | - 1.84^b^ (-6.86 to 3.00) |
| **EQ-5D-5L 6-months** | | | |
| n | 88 | 86 |  |
| Mean VAS score (SD) | 71.67 (20.67) | 70.20 (20.46) | 1.13 (-3.85 to 6.34) |
| **EQ-5D-5L 12-months** | | | |
| n | 89 | 85 |  |
| Mean VAS score (SD) | 69.03 (19.07) | 68.61 (21.68) | - 0.57 (-5.92 to 5.41) |
| CI, Confidence Interval; EQ-5D-5L, EuroQol 5-domain instrument 5-level; n, number of participants; SD, standard deviation; VAS, Visual Analogue Scale; VBL, Vein Bypass Ligation.  ^a^ Adjusted for baseline VAS score, age, the presence or absence of hepatic decompensation, size of the largest varix, presence or absence of alcohol related liver disease. Values >0 favour carvedilol in comparison to VBL.  ^b^ Adjusted for age, the presence or absence of hepatic decompensation, size of the largest varix, presence or absence of alcohol related liver disease. Values>0 favour carvedilol in comparison to VBL. | | | |

**Table S27:** Results of the economic evaluation analyses - base case

| **Analysis** | **Mean total (£) cost** | **Incremental (£) cost^a^** | **Mean outcome** | **Incremental outcome^b^** | **ICER**  **cost (£) per outcome** |
| --- | --- | --- | --- | --- | --- |
| **Cost-utility analysis (QALY)** | |  |  |  |  |
| Carvedilol n=72 | 1,628.07 (5,458.00) |  | 0.714(0.238) |  |  |
| VBL n=68 | 2,671.66 (2,052.67) | -1,052.94  (-2,097.50 to 14.80) | 0.693 (0.248) | 0.010  (-0.038 to 0.056) | Dominant^c^ |
| **Cost-effectiveness analysis (variceal bleeding avoided)^d^** | | | | | |
| Carvedilol n=109 | 2,406.06 (6,828.82) |  | 0.954 (0.210) |  |  |
| VBL n=105 | 3,993.05 (6,753.67) | -1,608.04  (-3,426.23 to 103.53) | 0.914 (0.281) | 0.041  (-0.020 to 0.108) | Dominant^c^ |

^a^Adjusted for age, the presence or absence of hepatic decompensation, size of the largest varix, presence or absence of alcohol related liver disease. Values <0 favour carvedilol in comparison to VBL

^b^Adjusted for baseline EQ-5D-5L score, age, the presence or absence of hepatic decompensation, size of the largest varix, presence or absence of alcohol related liver disease. Values >0 favour carvedilol in comparison to VBL

^c^Dominant = Intervention (carvedilol) is less costly and more effective that the comparator (VBL).

^d^ outcome represents the percentage of patients without bleeding within 12 months. The difference suggests that additional 4 patients in the carvedilol group will not have bleeding per 100 patients compared to those in the VBL group.

**Table S28:** Results of the scenario analyses

| **Analysis** | **Mean total (£) cost** | **Incremental (£) cost^a^** | **Mean outcome** | **Incremental outcome^b^** | **ICER**  **cost (£) per outcome** |
| --- | --- | --- | --- | --- | --- |
| **Cost of Carvedilol with no follow up visit at 2 weeks** | | | | | |
| Carvedilol n=72 | 1,684.07 (5,458.00) |  | 0.714(0.238) |  |  |
| VBL n=68 | 2,671.66 (2,052.67) | -996.94  (-2,015.34 to 460.03) | 0.693 (0.248) | 0.010  (-0.038 to 0.056) | Dominant^c^ |
| **Crosswalk mapping method** | | | | | |
| Carvedilol n=72 | 1,628.07 (5,458.00) |  | 0.716 (0.240) |  |  |
| VBL n=68 | 2,671.66 (2,052.67) | -1,052.94  (-2,097.50 to 14.80) | 0.701 (0.235) | 0.013  (-0.037 to 0.060) | Dominant^c^ |

^a^Adjusted for age, the presence or absence of hepatic decompensation, size of the largest varix, presence or absence of alcohol related liver disease. Values <0 favour carvedilol in comparison to VBL

^b^Adjusted for baseline EQ-5D-5L score, age, the presence or absence of hepatic decompensation, size of the largest varix, presence or absence of alcohol related liver disease. Values >0 favour carvedilol in comparison to VBL

^c^Dominant = Intervention (carvedilol) is less costly and more effective that the comparator (VBL).

^d^ outcome represents the percentage of patients without bleeding within 12 months. The difference suggests that additional 4 patients in the carvedilol group will not have bleeding per 100 patients compared to those in the VBL group.

**Figure S8**: Cost-Effectiveness plane for Carvedilol versus VBL – Complete case analysis

**Figure S9**: Cost-effectiveness acceptability curve. Indicates the probability of carvedilol being cost-effective across different willingness-to-pay thresholds per additional QALY

References:

1. British National Formulary (BNF). Available at: <https://bnf.nice.org.uk/drugs>

2. *NICE. Cirrhosis in over 16s: assessment and management (update). 2023* [*https://www.nice.org.uk/guidance/ng50/evidence/a-clinical-and-costeffectiveness-of-nonselective-betablockers-and-endoscopic-variceal-band-ligation-for-the-primary-prevention-of-bleeding-in-people-with-oesophageal-varices-due-to-cirrhosis-pdf-13181683358*](https://www.nice.org.uk/guidance/ng50/evidence/a-clinical-and-costeffectiveness-of-nonselective-betablockers-and-endoscopic-variceal-band-ligation-for-the-primary-prevention-of-bleeding-in-people-with-oesophageal-varices-due-to-cirrhosis-pdf-13181683358).).

3. *NHS National Schedule of Reference Costs 2021/22. URL:* [*https://www.england.nhs.uk/publication/2021-22-national-cost-collection-data-publication/*](https://www.england.nhs.uk/publication/2021-22-national-cost-collection-data-publication/). 1 March 2024).

4. Jones KCW, Helen; Birch, Sarah; Castelli, Adriana; Chalkley, Martin; Dargan, Alan; Forder, Julien E.; Gao, Minyue; Hinde, Seb; Markham, Sarah; Premji, Shainur; Findlay, D.; Teo, H. Unit costs of health and social care 2023 manual. 2024.

5. Hernández Alava M, Pudney S, Wailoo A. Estimating the relationship between EQ-5D-5L and EQ-5D-3L: results from a UK population study. *Pharmacoeconomics* 2023;**41**:199-207.

6. Drummond MF, Sculpher MJ, Claxton K, Stoddart GL, Torrance GW. *Methods for the economic evaluation of health care programmes*: Oxford university press; 2015.

7. *National Institute for Health Care Excellence. NICE health technology evaluations: the manual* [*www.nice.org.uk/process/pmg36*](https://bham-my.sharepoint.com/personal/d_tripathi_bham_ac_uk/Documents/Safestick/Clinical%20Trials/CALIBRE/CALIBRE%20publications/Final%20Paper/CALIBRE%20Paper%20Documents/AP&T%20Submission/www.nice.org.uk/process/pmg36) 2022).

8. Manca A, Hawkins N, Sculpher MJ. Estimating mean QALYs in trial‐based cost‐effectiveness analysis: the importance of controlling for baseline utility. *Health economics* 2005;**14**:487-96.

9. Black WC. The CE plane: a graphic representation of cost-effectiveness. *Medical decision making* 1990;**10**:212-4.

10. Glick HA, Briggs AH, Polsky D. Quantifying stochastic uncertainty and presenting results of cost-effectiveness analyses. *Expert review of pharmacoeconomics & outcomes research* 2001;**1**:25-36.

11. Van Hout BA, Al MJ, Gordon GS, Rutten FF. Costs, effects and C/E‐ratios alongside a clinical trial. *Health economics* 1994;**3**:309-19.

12. StataCorp. 2021. Stata Statistical Software: Release 18. College Station, TX: StataCorp LLC.

13. Van Hout B, Janssen M, Feng Y-S, Kohlmann T, Busschbach J, Golicki D*, et al.* Interim scoring for the EQ-5D-5L: mapping the EQ-5D-5L to EQ-5D-3L value sets. *Value in health* 2012;**15**:708-15.

14. Mattock R, Tripathi D, O'Neill F, Craig J, Tanner J, Patch D*, et al.* Economic evaluation of covered stents for transjugular intrahepatic portosystemic stent shunt in patients with variceal bleeding and refractory ascites secondary to cirrhosis. *BMJ open gastroenterology* 2021;**8**:e000641.

15. Cullen K, Jones M, Pockett RD, Burton A, Cross TJ, Rowe IA*, et al.* Cost of hepatocellular carcinoma to the national health service in England: a registry-based analysis. *BMJ Open Gastroenterology* 2023;**10**:e000998.
